# Selection for long and short sleep duration in *Drosophila melanogaster* reveals the complex genetic network underlying natural variation in sleep

**DOI:** 10.1371/journal.pgen.1007098

**Published:** 2017-12-14

**Authors:** Susan T. Harbison, Yazmin L. Serrano Negron, Nancy F. Hansen, Amanda S. Lobell

**Affiliations:** 1 Laboratory of Systems Genetics, National Heart Lung and Blood Institute, National Institutes of Health, Bethesda, MD, United States of America; 2 Comparative Genomics Analysis Unit, National Human Genome Research Institute, National Institutes of Health, Rockville, MD, United States of America; Stanford University School of Medicine, UNITED STATES

## Abstract

Why do some individuals need more sleep than others? Forward mutagenesis screens in flies using engineered mutations have established a clear genetic component to sleep duration, revealing mutants that convey very long or short sleep. Whether such extreme long or short sleep could exist in natural populations was unknown. We applied artificial selection for high and low night sleep duration to an outbred population of *Drosophila melanogaster* for 13 generations. At the end of the selection procedure, night sleep duration diverged by 9.97 hours in the long and short sleeper populations, and 24-hour sleep was reduced to 3.3 hours in the short sleepers. Neither long nor short sleeper lifespan differed appreciably from controls, suggesting little physiological consequences to being an extreme long or short sleeper. Whole genome sequence data from seven generations of selection revealed several hundred thousand changes in allele frequencies at polymorphic loci across the genome. Combining the data from long and short sleeper populations across generations in a logistic regression implicated 126 polymorphisms in 80 candidate genes, and we confirmed three of these genes and a larger genomic region with mutant and chromosomal deficiency tests, respectively. Many of these genes could be connected in a single network based on previously known physical and genetic interactions. Candidate genes have known roles in several classic, highly conserved developmental and signaling pathways—EGFR, Wnt, Hippo, and MAPK. The involvement of highly pleiotropic pathway genes suggests that sleep duration in natural populations can be influenced by a wide variety of biological processes, which may be why the purpose of sleep has been so elusive.

## Author summary

One of the biggest mysteries in biology is the need to sleep. Sleep duration has an underlying genetic basis, suggesting that very long and short sleep times could be bred for experimentally. How far can sleep duration be driven up or down? Here we achieved extremely long and short night sleep duration by subjecting a wild-derived population of *Drosophila melanogaster* to an experimental breeding program. At the end of the breeding program, long sleepers averaged 9.97 hours more nightly sleep than short sleepers. We analyzed whole-genome sequences from seven generations of the experimental breeding to identify allele frequencies that diverged between long and short sleepers, and verified genes and genomic regions with mutation and deficiency testing. These alleles map to classic developmental and signaling pathways, implicating many diverse processes that potentially affect sleep duration.

## Introduction

Sleep remains a classic enigma in biology. Intense study has only begun to reveal the physiological needs that sleep might satisfy [1, 2]. One potential function of sleep is to conserve resources [3, 4]. Sleep may increase protein synthesis [5] or downscale wake-active synapses [6]. Physical remodeling in the brain during sleep may alter brain plasticity and enable the consolidation of memories [6, 7]. Metabolic wastes that accumulate during waking may also be eliminated during sleep [8]. If some or all of these activities are accomplished during sleep, it would ensure the balance of proper energetic resources and avoid the accumulation of waste products.

Another possibility is that sleep is crucial for proper development. Babies and young infants spend far more time asleep than their adult counterparts [9], and interfering with sleep in young animals disrupts critical fitness and cognitive behaviors in adults [10, 11]. These observations suggest that sleep might be a fundamental property of local neuronal physiology [12], and the need to sleep is established during development with the formation of neuronal structures.

Sleep-like behaviors are widely conserved among species [1, 2, 13–15], but the type and amount of sleep differs across species [14–16]. Sleep duration is also variable within a species [17, 18], with related individuals having more similar sleep than unrelated individuals [19–27]. This indicates that sleep duration is under at least partial genetic control [19–28]. Yet sleep parameters can also vary considerably within an individual at different times [29–31]. This variation may be driven by ecological demands. For example, during periods of migration over the ocean, frigate birds sleep less often and less intensely [32]. Male pectoral sandpipers sleep little during the competitive mating season [33], and those that sleep less sleep more intensely [34]. Cetacean mothers and their newborn calves may also have reduced sleep with no apparent effects of sleep loss [35, 36], though these findings are controversial due to the challenges of observing sleep accurately in these mammals [37, 38]. In addition, night sleep and sunrise anticipation vary with latitude in flies, suggesting that they are modified by the environment [39]. Taken together, these observations suggest that sleep exhibits environmental plasticity [40]; that is, sleep can be modified when ecological demands require it. Yet environmental plasticity has an underlying genetic component [41]; thus, genotype influences the amount of adaptation that is possible. Here we wanted to determine how far night sleep duration could be driven up or down in constant environmental conditions using a combination of naturally occurring alleles, and to identify allelic variants responsible for the changes.

Previous work has demonstrated the value and utility of *D*. *melanogaster* as a model for mammalian sleep [42, 43]. The use of flies as a model has made several large-scale mutagenesis and gene knockdown screens [44–51], genome-wide association mapping [18], quantitative trait transcript mapping [52], and gene expression profiling [53–55] possible, identifying unprecedented numbers of novel candidate genes putatively involved in sleep. The potential to perturb sleep duration to high or low extremes may be greater in *D*. *melanogaster*, which lacks the genomic redundancy seen in mammals. Indeed, extremely low sleep duration has been observed in flies with single mutations in *Shaker* (*Sh*), *sleepless* (*sss*), *insomniac* (*inc*), and *nicotinic Acetylcholine Receptor α4* (*nAChRα4*) [44, 45, 50, 51].

The short generation time of flies makes it possible to combine artificial selection or laboratory evolution with whole-genome sequencing to understand the genetic basis of complex traits. Such a strategy has been used to explore adaptation to different environments [56–60], male courtship song [61], body size variation [62], accelerated development [63], viral adaptation [64], and food consumption [65]. Artificial selection for a combination of reduced sleep duration, increased sleep latency, and increased activity produced flies with behavioral phenotypes mimicking insomnia [66]. We have applied this strategy to drive night sleep duration to long and short extremes and to examine the underlying genomic changes. In the final generation of selection, there was a pronounced difference in night sleep duration, 9.97 hours on average, between the longest-sleeping and shortest-sleeping populations. There was some indication that flies selected for short sleep had perturbed circadian rhythms as well, while flies selected for long sleep remained diurnal. Selection for long or short night sleep altered several other sleep traits such as day sleep duration and night average bout length. Both long- and short-sleepers did not respond to a mild sleep deprivation stimulus with increased recovery sleep, suggesting that either the sleep homeostat was altered, or that increased sleep intensity compensated for the loss of sleep. Long and short night sleepers had normal lifespan and egg-to-adult viability, suggesting that there is little physiological consequence to being a long or short sleeper. Yet fewer animals from each population (including the control populations) survived sleep monitoring with each successive generation of selection, indicating that either inbreeding depression or stress sensitivity might limit how far up or down sleep can be driven. DNA sequence from each selection population over seven generations of selection revealed genome-wide changes the underlying allele frequencies, with thousands of polymorphisms significantly changing in frequency between any two generations of selection. However, regressing allele frequency changes across generations and accounting for potential effects of random genetic drift reduced the number of candidate polymorphisms to 126. These polymorphisms are located within ± 1 kb of 80 candidate genes, two of which overlapped with candidate genes for sleep duration in a previous genome-wide study of sleep [18]. Candidate genes mapped to classic developmental and signaling pathways, and we verified candidate genes and regions by testing mutations and chromosomal deficiencies. Connecting these genes to known genetic interactions suggest that they may impact a larger network of genes controlling sleep duration.

## Results

### Phenotypic response to selection for long or short night sleep duration

**Fig 1:**
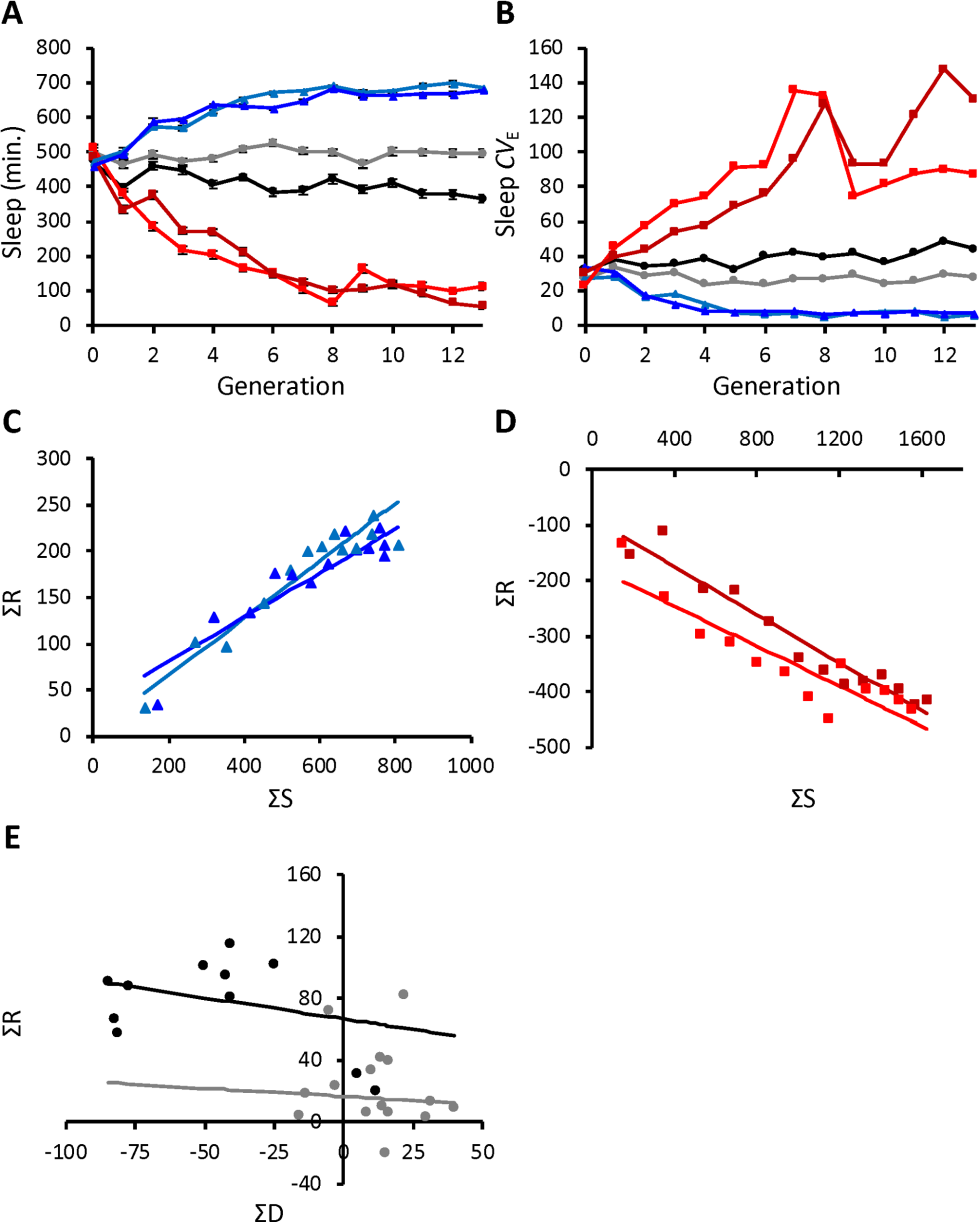
Phenotypic response to artificial selection for night sleep duration. (A), combined-sex average night sleep duration ± SE is plotted for each generation of selection; (B), combined-sex night sleep coefficient of environmental variation (*CV*_E_) is plotted for each generation of selection; (C) and (D), combined-sex cumulative selection differential (ΣS) versus combined-sex cumulative response (ΣR) for (C) long sleep and (D) short sleep populations; (E), combined-sex cumulative differential (ΣD) versus combined-sex cumulative response (ΣR) for the control populations. Light blue and dark blue triangles indicate Replicate 1 and Replicate 2 populations selected for long sleep; Light red and dark red squares indicate Replicate 1 and Replicate 2 populations selected for short sleep; and light gray and black circles indicate Replicate 1 and Replicate 2 control populations.

To determine how far night sleep duration can be driven up or down, we applied an artificial selection protocol to flies from the Sleep Advanced Intercross Panel (SAIP) constructed from the most extreme long- and short-sleeping lines of the Drosophila Genetic Reference Panel (DGRP). Sleep characteristics were uniform in the outbred population (S1 Fig). We selected two replicate populations for long night sleep, two for short night sleep, and two populations were maintained as unselected controls. Sleep was measured in 100 virgin males and 100 virgin females of each population each generation. The 25% most extreme long (short) sleepers were chosen as parents for the next generation of the long (short) sleeping populations. Control populations were maintained by choosing 25% of the males and females at random to be parents for the next generation. Night sleep duration, defined as sleep during the lights-off period, ranges from 0 to 12 hours (720 minutes). Flies responded rapidly and dramatically to 13 generations of artificial selection (Fig 1A; *P* = 0.0002; S1 and S2 Tables). Night sleep in the short-sleeping populations was reduced to 111.9 ± 10.74 minutes (replicate 1) and 54.8 ± 5.66 minutes (replicate 2) by generation 13. In contrast, night sleep in the long-sleeping populations was increased to 685.0 ± 3.35 (replicate 1) and 678.5 ± 3.46 minutes (replicate 2) in the same generation. Night sleep differed by 598.4 minutes (9.97 hours) on average between long sleepers and short sleepers. The phenotypic response was moderately asymmetrical in the direction of decreased night sleep (*P* = 0.0344; Fig 1A). Unselected control populations averaged 495.9 ± 11.71 (replicate 1) and 364.9 ± 11.99 (replicate 2) minutes of night sleep at generation 13 and were not significantly different from night sleep in the outbred population prior to selection (Fig 1A; S3 Table). The artificial selection procedure was equally effective in both males and females across all generations (S1 Table), though some sex-specific differences were observed for separate generations (S2 Table). Females were more responsive than males to selection in generations 1, 3, 4, and 6, increasing the differential between long and short night sleep by as much as 72 minutes more than males. We modeled the change in variability in sleep among individuals over time as the coefficient of environmental variation (*CV*_E_) because artificial selection, which uses only a subset of each population as parents for the next generation, tends to reduce phenotypic variance [67]. Interestingly, night sleep *CV*_E_ had a significant correlated response to selection for night sleep (Fig 1B, *P* <0.0001), increasing in flies selected for short night sleep and decreasing in flies selected for long night sleep. The estimated realized heritabilities *h*^2^, which indicate the degree to which the animals responded to the selection procedure, were relatively high for long-sleepers [65, 68–70]; *h*^2^ = 0.310 ± 0.022 and *h*^2^ = 0.238 ± 0.032 (all *P* <0.0001) for replicates 1 and 2, respectively (Fig 1C). For short sleepers, the realized heritabilities were *h*^2^ = 0.179 ± 0.026 and *h*^2^ = 0.215 ± 0.017 (all *P* <0.0001) (Fig 1D). In addition, the regression of the control after thirteen generations of breeding with random parents was not significant, {-0.108 ± 0.312 (*P* = 0.7368) and -0.271 ± 0.206 (*P* = 0.2161) for replicates 1 and 2 (Fig 1E)}, suggesting that inbreeding depression did not impact these populations [67]. Thus, the outbred population, which was derived from DGRP lines with the largest mean differences in night sleep duration responded rapidly to artificial selection for long or short night sleep. This heritable response indicates that these populations will be informative for identifying genes and pathways involved in night sleep duration.

### Correlated response of other sleep parameters to selection for long or short night sleep duration

**Fig 2:**
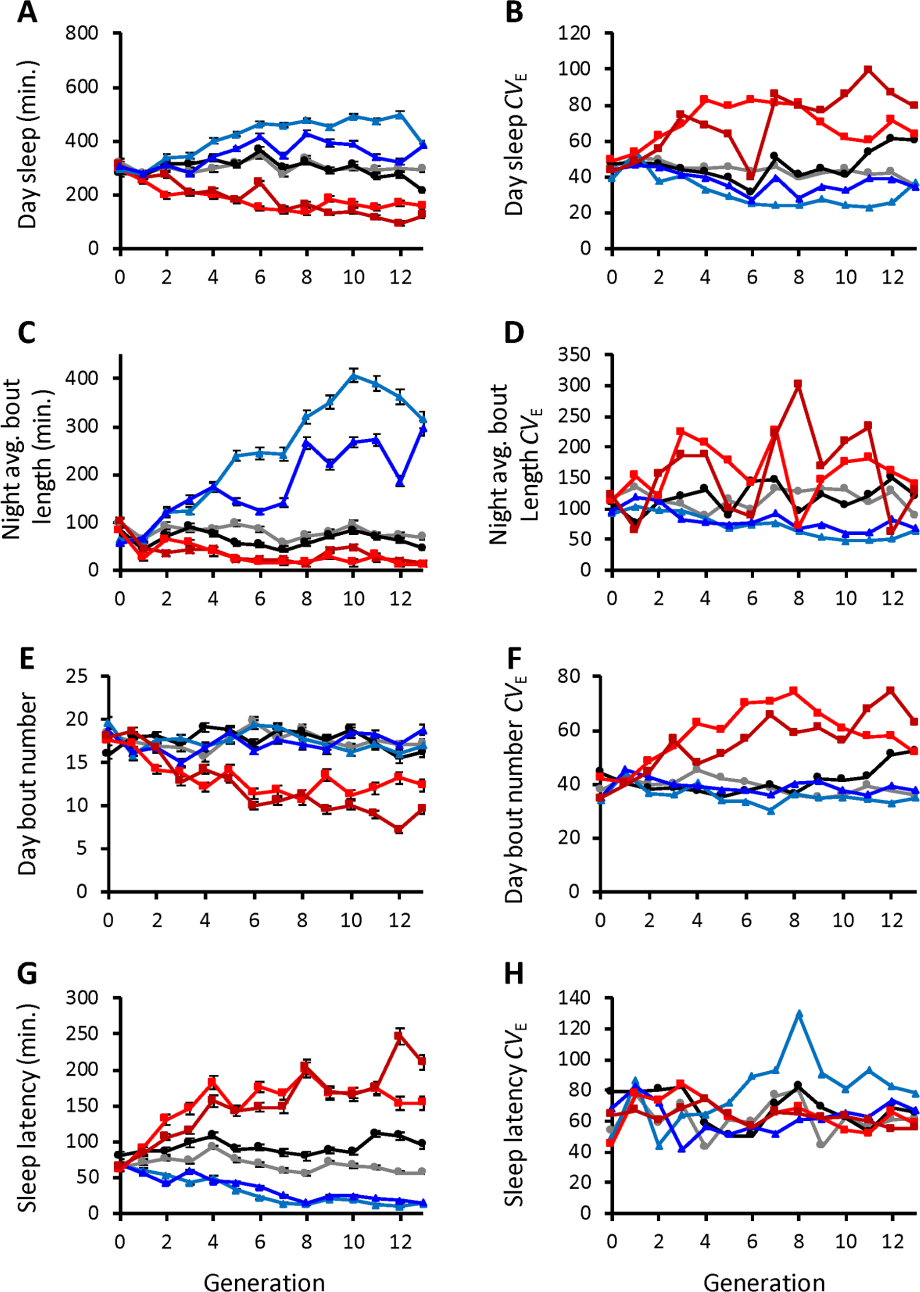
Sleep traits with a significant correlated response to artificial selection for long or short night sleep duration. (A, C, E, G), the combined-sex mean sleep trait ± SE is plotted for each generation of selection; (A), day sleep duration; (C), night average bout length; (E), day bout number, and (G) sleep latency. (B, D, F, H), the combined-sex sleep trait coefficient of environmental variation (*CV*_E_) is plotted for each generation of selection; (B), day sleep *CV*_E_; (D), night average bout length *CV*_E_; (F), day bout number *CV*_E_; (H), sleep latency *CV*_E_. Colors and symbols are the same as Fig 1.

Many different characteristics of sleep are at least partially genetically correlated with night sleep duration [18, 52, 71], suggesting that they share some of the same genetic architecture. If selection acts on genes common to these traits, then these traits might also react to artificial selection for long or short night sleep, which is known as a correlated response to selection [67]. We found that day sleep duration (*P* = 0.0248), night average bout length (*P* = 0.0358), day bout number (*P* = 0.0121), and sleep latency (*P* = 0.0005) responded to selection for long or short night sleep, even though we did not select for these changes (Fig 2A, 2C, 2E and 2G; S1 Table). Day sleep, night average bout length, and day bout number responded in the same direction as selection for night sleep: for instance, selection for longer night sleep resulted in longer day sleep, longer night average bout length, and greater numbers of day bouts. Sleep latency, the amount of time to the first sleep bout after lights are turned off, responded in the opposite direction to selection. Specifically, selection for short night sleep resulted in very long sleep latencies, which might be anticipated if a shift in circadian behavior was also present (see below). Night bout number, day average bout length, and waking activity did not exhibit a significant correlated response to selection for night sleep, suggesting that the genes underlying these traits were unaffected by selection (S2 Fig). These trends were generally true for each generation of selection when considered separately, though we observed transient and sometimes sex-specific correlated responses of day average bout length (over 4 generations) and waking activity (in one generation) with selection (S2 Table). In addition, day sleep *CV*_E_ (*P* < 0.0001), night average bout length *CV*_E_ (*P* < 0.0001), day bout number *CV*_E_ (*P* <0.0001), and sleep latency *CV*_E_ (*P* = 0.0401) had a significant correlated response to selection for night sleep (Fig 2B, 2D, 2F and 2H; S4 Table), while night bout number *CV*_E_, day average bout length *CV*_E_, and waking activity *CV*_E_ did not (S2B, S2D and S2F Fig; S4 Table). Thus, many parameters describing sleep architecture responded to selection for long or short night sleep duration. Notably, night bout number was previously shown to be negatively correlated with night sleep [18, 71], but it did not respond to the selection procedure in this experiment. Overall, many sleep traits were altered by artificial selection for night sleep, suggesting a shared genetic architecture.

### Changes in sleep architecture with selection

**Fig 3:**
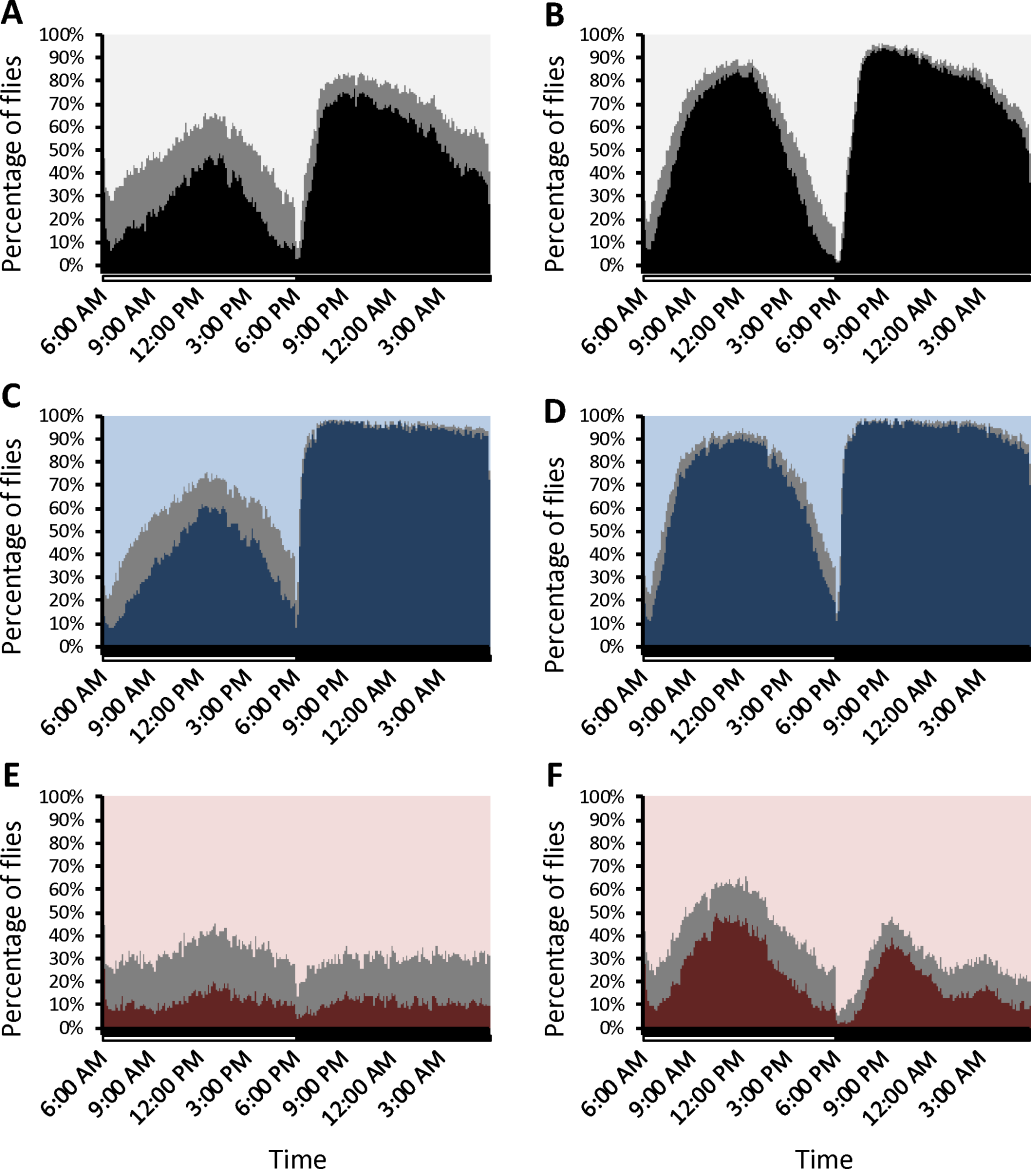
Sleep architecture in selected populations. Shown are the percentages of flies sleeping, awake, or in a transient pause for each minute in a 24-hour day. (A), control females; (B), control males; (C), long sleeper females; (D), long sleeper males; (E), short sleeper females; (F), short sleeper males. Black/dark blue/dark red = sleep; gray = transient pause; light gray/light blue/light red = wake. White and black bars on the x-axis indicate the light and dark periods, respectively. Replicate 1 populations are shown; replicate 2 populations can be found in S3 Fig.

We examined the selection populations to determine whether changes in night sleep duration extended to sleep architecture, i.e., the overall distribution of sleep bouts. We analyzed sleep architecture for each sex separately as characteristic sex-specific differences are well-known in flies [18, 72–75]. Males tend to have pronounced periods of high activity during light-dark transitions and a long siesta during the day, while females have a more uniform distribution of sleep and activity during the day [76]. We plotted the percentages of flies that were sleeping, awake, or in a transient pause (1 to 4 minutes of immobility) during each minute of the day (Fig 3 and S3 Fig). Control flies behaved as expected: both males and females were more likely to be asleep during the first third of the night [42], and males were more likely to be asleep during the day than females (Fig 3A and 3B). The same pattern is evident in the long-sleeper populations, except that a much greater percentage of flies were likely to be sleeping during the night; most males and females slept right up to the lights-on period (Fig 3C and 3D). Sleep in female short sleepers, however, did not exhibit these patterns; instead, the trend was flat, with 7% of the flies asleep at any given time on average (Fig 3E). Male short-sleepers had an almost bimodal distribution of sleep during the night, with the highest propensity for sleep during the first third of the night (Fig 3F). However, more males were likely to be asleep at midday than the first third of the night, suggesting that the males were somewhat nocturnal in their activity patterns. Short sleepers did not have more fragmented sleep than long sleepers, however. Both short and long sleepers had reduced night bout number relative to the control population (S2A Fig), suggesting that the patterns of sleep bouts were more consolidated in both groups of flies. Night average bout length was reduced in short sleepers and increased in long sleepers (Fig 2C). Thus, while sleep architecture in long sleepers was similar to that of the unselected control, some disruption of the circadian clock was evident in the short sleepers. The differences in sleep architecture suggest that the artificial selection protocol affected both sleep and circadian behavior.

### Response of life history traits to selection for long or short night sleep duration

**Fig 4:**
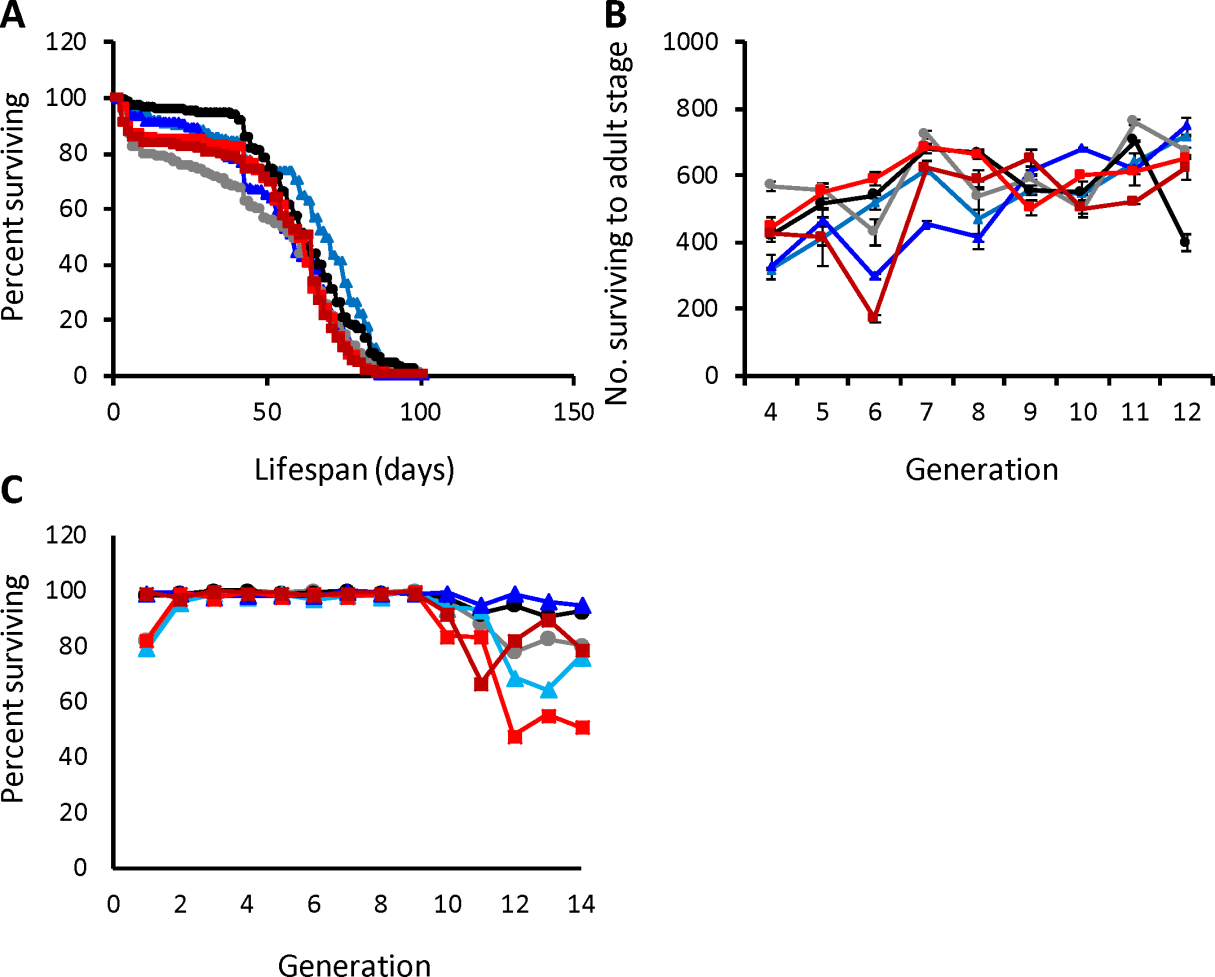
The response of life history traits to selection for long or short night sleep duration. (A), percentage flies surviving versus lifespan; (B), number of flies surviving to the adult stage versus generation of selection; (C), percentage of males and females surviving sleep assay. Colors and symbols are the same as Fig 1.

Sleep is crucial for life, yet its relationship to important life history and fitness traits is not well understood. Several previous mutagenesis screens have noted reduced lifespan in mutants with short sleep duration [44, 45, 49, 51, 77, 78], though there are exceptions [51, 79]. We measured lifespan in all six selection populations; in contrast to the reduced lifespan seen in short-sleeping mutants, we found no significant differences in lifespan for either sex in any of the selection populations (Fig 4A; S5 Table). If we assume that sleep is associated with fitness, an asymmetrical response to selection would indicate reduced fitness in the direction of the greater response to selection [67]. Thus, we would predict that short-sleeping flies would be less fit than long-sleeping ones. To investigate this possibility, we measured egg-to-adult viability as a proxy for fitness. We found no differences among selection populations (Fig 4B; S5 Table). However, we noted a propensity for flies to die during sleep monitoring in the latter generations of the experiment (Fig 4C). Over the course of the entire experiment there were no significant differences among populations in the numbers of flies surviving, but there were significant differences in survival at generations 3 (*P* = 0.0429), 9 (*P* = 0.0352) and 10 (*P* = 0.0455). Short-sleeping females were the most vulnerable, though flies of all populations were less likely to survive the sleep monitoring. Thus, any physiological consequences of being an extreme long or short sleeper did not manifest themselves in either lifespan or egg-to-adult viability, but the reduced survival of short sleepers during the later generations of selection suggests that they might be more susceptible to stress.

### Phenotypic response of the selection populations to sleep deprivation

**Fig 5:**
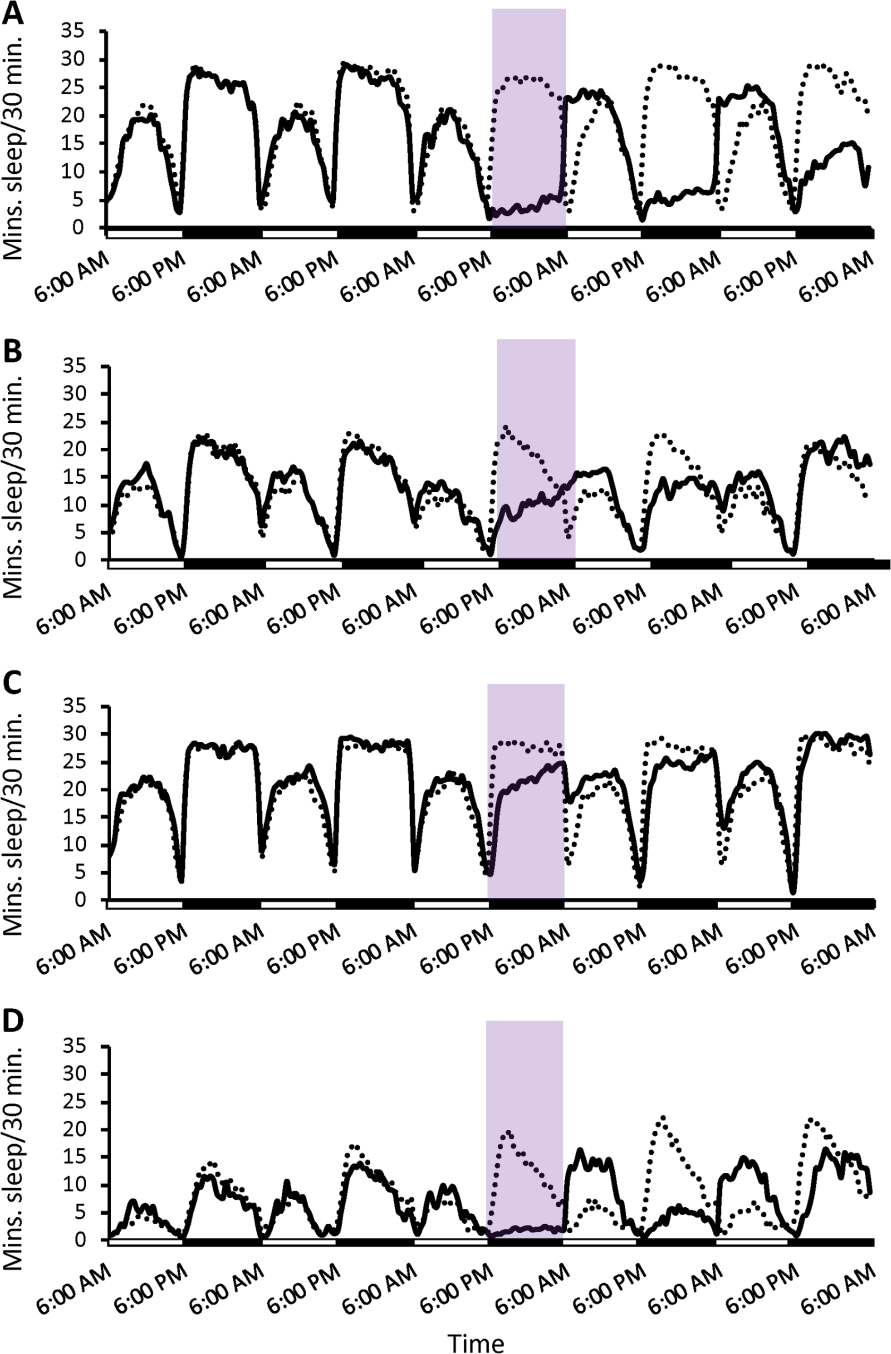
Sleep patterns of long- and short-sleepers after 12 hours of sleep deprivation. Average minutes of sleep per 30 minutes are plotted for control (dotted lines) and deprived (solid lines) flies. Purple shading indicates the application of the shaking stimulus. Shaded bars along the x-axis indicate day (white) and night (black). (A) Long sleeper replicate 1. (B) Short sleeper replicate 1. (C) Long sleeper replicate 2. (D) Short sleeper replicate 2.

**Fig 6:**
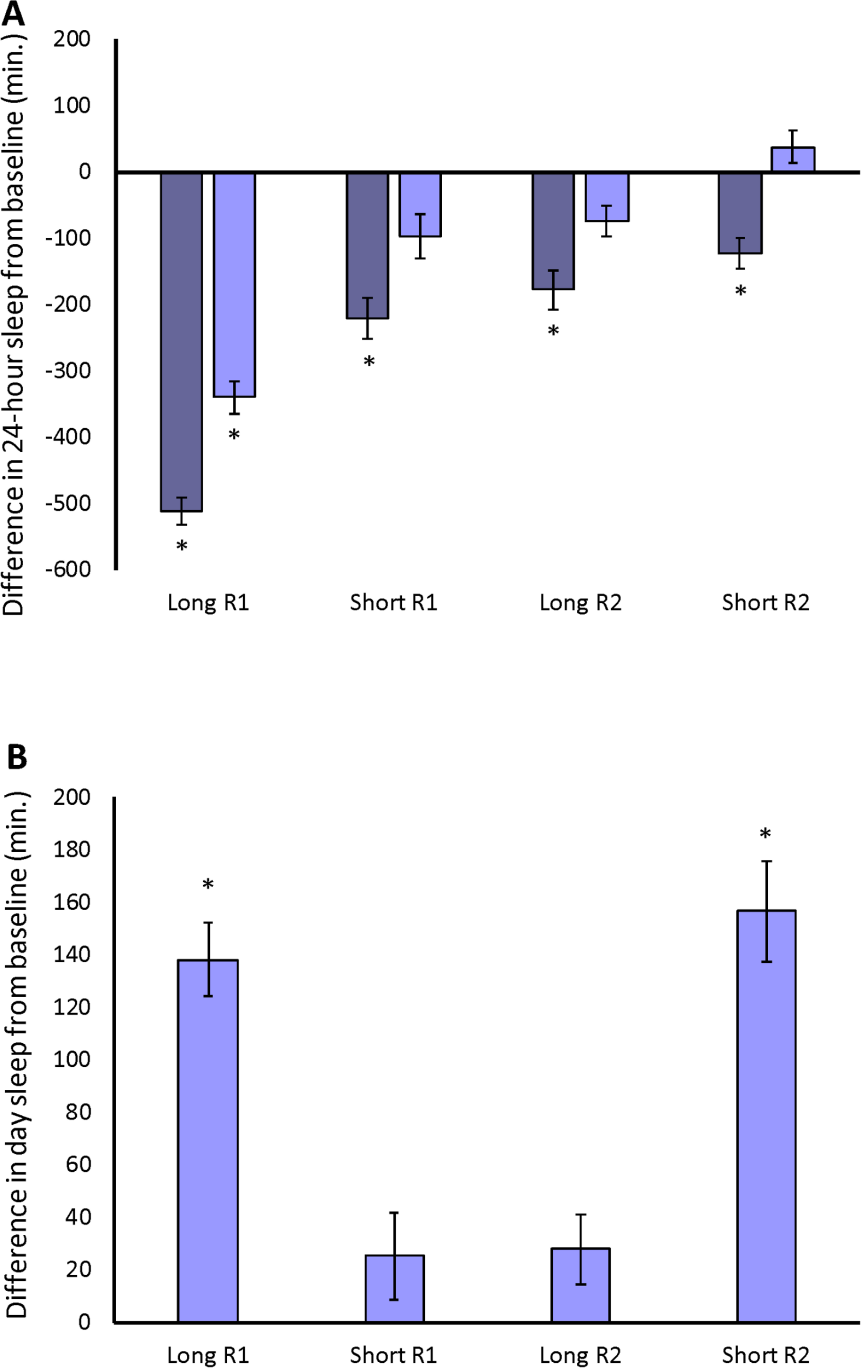
The response of long- and short-sleeper populations to 12 hours of sleep deprivation. (A), the difference in 24-hour sleep from baseline (the average of days 1 and 2) sleep is plotted for day 3, when the mechanical stimulus was applied (dark blue bars) and for day 4, the recovery day (light blue bars). (B), the difference in day sleep from baseline for day 4, the recovery day. * *P* < 0.05; *P*-values reflect the comparison of deprived and/or recovery day sleep with baseline sleep.

One of the hallmarks of sleep need is an increase in sleep, or sleep rebound, when normal sleep has been disrupted [42, 43]. To assess sleep need, we used a mechanical shaker to gently perturb sleep during the 12-hour night cycle in the long- and short-sleeping populations. We monitored sleep for two days prior to the mechanical stimulus (days 1,2, and the day cycle of day 3), during the stimulus (the night of day 3), and for two days after the stimulus (days 4 and 5). We expected to observe a decrease in sleep during the application of the mechanical stimulus, and an increase in sleep during the day after sleep deprivation in addition to normal, unperturbed sleep over the 24-hour period following the stimulus. We conducted this experiment after the artificial selection procedure had been relaxed for 47 generations, i.e., at Generation 60. We noted that after relaxation of selection, the short sleeper lines had an unexpected increase in night sleep as compared to Generation 13 flies: night sleep was 412.8 ± 16.23 minutes for replicate 1, and 186.0 ± 17.47 minutes for replicate 2. This increase in sleep has important implications for the relationship between sleep and fitness (see Discussion). All four populations responded to the shaking stimulus, as indicated by the decrease in night sleep on day 3 (Figs 5 and 6A), and controls were relatively unperturbed by comparison (*P*_Treatment_ and *P*_Treatment×Day_ < 0.0001 for night sleep in all populations) (S4 Fig). Long-sleeper flies of replicate 1 had a pronounced response to the sleep deprivation as their night sleep was reduced by 86% to 84.7 ± 18.49 minutes during the night. Although sleep increased during the day period after the mechanical stimulus was applied relative to baseline days 1 and 2 (Fig 6B), their sleep in the subsequent 24-hour period was actually reduced compared to baseline sleep (Fig 6A). Short sleepers of replicate 1 were less perturbed by the shaking procedure; their sleep dropped to 215.01 ± 27.70 minutes, which was only a 48% decrease in night sleep (Fig 5B). They did not respond with a significant increase in sleep over the subsequent 24-hour period (Fig 6A and 6B). The response of long-sleeper flies of replicate 2 to sleep disruption was similar to that of short-sleeper flies of replicate 1: a mild sleep loss of 25.7% on day 3 (Fig 5C), with a return to their normal sleep without a rebound (Fig 6A and 6B). Short sleepers of replicate 2 had almost no sleep during the shaking stimulus (Fig 5D). Their sleep was reduced to 33.7 ± 13.12 minutes of sleep, an 81.8% loss. Day sleep increased significantly after sleep duration, though the 24-hour sleep on the recovery day remained unchanged as compared to baseline (Fig 6A and 6B). Interestingly, both long sleepers of replicate 1 and short sleepers of replicate 2 had greatly disrupted sleep patterns, with longer periods of day sleep and shorter periods of night sleep (Fig 5A and 5D). Thus, all four populations lost sleep after mechanical perturbation, but their 24-hour sleep did not increase on the recovery day as was expected.

### Allele frequencies in the outbred population

As variability in night sleep duration has a genetic component, we expected that changes in night sleep duration over time would result from changes in allele frequencies in the selected populations. We therefore extracted and sequenced DNA from pools of flies sampled from seven generations: 0, 1, 2, 5, 8, 10, and 12. The starting inbred lines we used to construct the outbred population are sequenced; thus, the number of polymorphisms segregating within the population was known. 2,222,264 polymorphisms were expected to segregate among the 10 DGRP lines that we used [80, 81]. In addition, we used LoFreq [82] to identify additional potential rare or *de novo* polymorphisms that might be present in our selected populations; LoFreq detected an additional 258,268 potential polymorphisms (Materials and Methods). We defined major and minor alleles in our population by summing the allele counts for all polymorphisms across populations for generation zero, the generation prior to the start of selection for night sleep. Minor allele frequency distributions were fairly flat across all five chromosome arms (S5 Fig); 151,694 DGRP polymorphic sites were fixed for the major allele at generation zero of the outbred population. Thus, 2,328, 838 segregating sites could potentially change allele frequency across generations.

### Allele frequency change in selected populations

**Fig 7:**
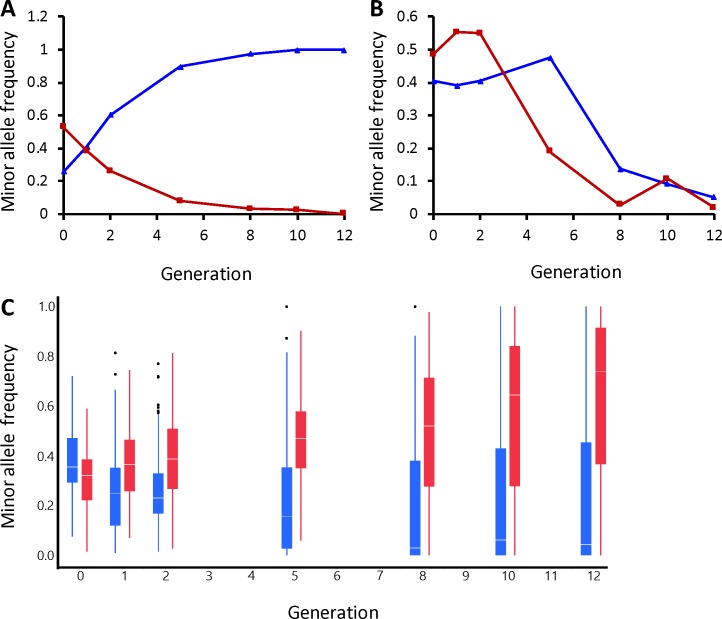
Examples of allele frequency trajectories observed across generations. Each plot shows the minor allele frequency for the long- and short-sleep selection schemes plotted against generation. Minor allele frequency was defined using the combined allele frequencies for all populations prior to selection (i.e., generation 0). (A), divergent trajectory at position 14,976,564 on chromosome *3L*; (B) similar trajectory at position 6,300,126 on chromosome *X*. Dark blue triangles indicate the average minor allele frequencies for long sleep Replicate 1 and Replicate 2 populations; Dark red squares indicate average minor allele frequencies for short sleep Replicate 1 and Replicate 2 populations. (C) box plots for all of the polymorphisms significant for the logistic regression versus generation. Blue, long sleep; red, short sleep.

We used the Cochran-Mantel-Haenszel (CMH) test to detect significant changes in allele frequency between any two generations for each replicate population [83]; forms of this test have been applied previously in artificial selection and laboratory evolution experiments [56, 59, 63, 64]. Large numbers of polymorphisms across the entire genome were statistically significant for the CMH test at a Bonferroni-corrected threshold *P* value (2.3 × 10^−8^) (Table 1). We therefore used the following strategies to identify the polymorphisms that were most likely to be the targets of selection. As a first step, we identified significant polymorphisms in the long- and short-sleeper populations that were also significant in the control populations. The allele frequency changes in these overlapping polymorphisms cannot be distinguished from random genetic drift or environmentally-mediated adaptation, so we eliminated them from consideration. Second, we examined the manner in which allele frequencies from long- and short- sleeper populations changed across generations. We observed many different types of trajectories for polymorphisms having significant allele frequency changes. In some cases, allele frequency changes sometimes diverged between the long- and short-sleeper populations over time. Fig 7A shows this divergence; the average minor allele frequency increased over time in the long-sleeper populations to reach fixation for the minor allele at generation 12, while the average minor allele frequency decreased over time in the short-sleeper populations to reach fixation for the major allele at generation 12. We also observed trajectories in which the minor allele frequencies for both types of selection exhibited the same trend over time, such as that depicted in Fig 7B. We hypothesized that divergent allele frequency patterns such the one depicted in Fig 7A were more likely to be targets of selection. We therefore combined the data into a logistic regression analysis that incorporated minor allele frequency changes over time for 68,971 polymorphisms and 217 indels in the long and short selection populations that had significant changes across any two generations by the CMH test and did not change allele frequency in the control populations. 126 polymorphisms were significant for the logistic regression (S6 Table). Interestingly, there was a strong propensity for selection against the minor allele in the long-sleeper populations, and selection for the minor allele in the short-sleeper populations (Fig 7C); 111 of the 126 polymorphisms had higher minor allele frequencies in short sleepers than in long sleepers. In addition, we conducted simulations to assess the impact of random genetic drift on these polymorphisms (Methods). We determined the magnitude of allele frequency change that could occur due to drift, given the starting allele frequency at generation 0. These simulations suggested that 59 of the 126 polymorphisms exceeded the upper limit of allele frequency changes that would be expected from drift alone (S7 Table). Combining the allele frequency data across selection populations and across generations therefore enabled us to pinpoint a small number of likely selection targets, and considering the upper bound of random drift narrowed the number of candidate variants even further, to 59.

**Table 1:** Polymorphisms with significant allele frequency changes across generations.

Selection scheme	Replicate 1	Replicate 2	Overlap
Short	343,277	219,387	41,110
Control	138,891	118,451	13,357
Long	250,064	182,284	36,245

### Estimated linkage disequilibrium effects

It is quite likely that linkage disequilibrium (LD), the non-random segregation of allelic variants at two loci, affects our results. Estimating LD using pooled sequence data is a difficult challenge as the haplotypes of each fly are not known [84]. Although local LD decays within 10 to 30 bp on average in the DGRP [80], thousands of polymorphisms were in long-range (i.e., greater than 1kb) LD with a given variant, particularly if that variant was at low frequency [81]. To create the SAIP, flies were randomly mated for 21 generations, so there would be considerable heterozygosity in the genomes of these flies. Variants unique to a single fly would be in *de facto* LD with the remainder of the population [85]. Moreover, recombination rates vary with genotype [86, 87], and high recombination can be localized to different positions in the genome based on genotype [86], suggesting that rates of recombination could change across any two generations of selection. However, polymorphisms in high LD tend to have allele frequencies that are similar to one another [88–90]. This observation makes it possible to make a rough estimate of the upper and lower bounds for the minor allele frequency at variant B that would be required to observe LD at variant A, provided that the *r*^2^ and minor allele frequency at variant A are specified [89]. We therefore applied the following strategy to estimate the impact of LD on our results. First, we calculated LD among 18,000 randomly chosen variants in the 10 DGRP lines used to construct the SAIP; we were able to calculate LD directly in this case as the gametic phase of lines of the DGRP is known. There were 2,670,445 polymorphic pairs in high LD (*r*^2^ ≥ 0.8); this is 1.65% of the possible 161,991,000 pairwise combinations, indicating that LD among the ten DGRP lines is quite low. Next, we estimated the change in LD among these 2,670,445 polymorphic pairs after the 21 generations of random mating used to construct the SAIP. We binned the minor allele frequencies of the SAIP into 0.01-increments from 0 to 0.5. For each of the polymorphic pairs, we calculated the range of allele frequencies *p*_b_ at variant B that would result in high LD (i.e., an *r*^2^ of 0.8 or greater) given a binned allele frequency *p*_a_ at variant A (Methods) [89]. At generation 0, the number of variant pairs that were still in LD had decreased to 246,779 on average in the control populations, 247,483 in the long-sleeper populations, and 232,998 in the short-sleeper populations. These estimates suggest that much of the LD in the 10 DGRP lines decreased during the 21 generations of random mating used to construct the SAIP. Based on this random sampling we expected the polymorphisms in the starting outbred populations to be relatively independent.

**Fig 8:**
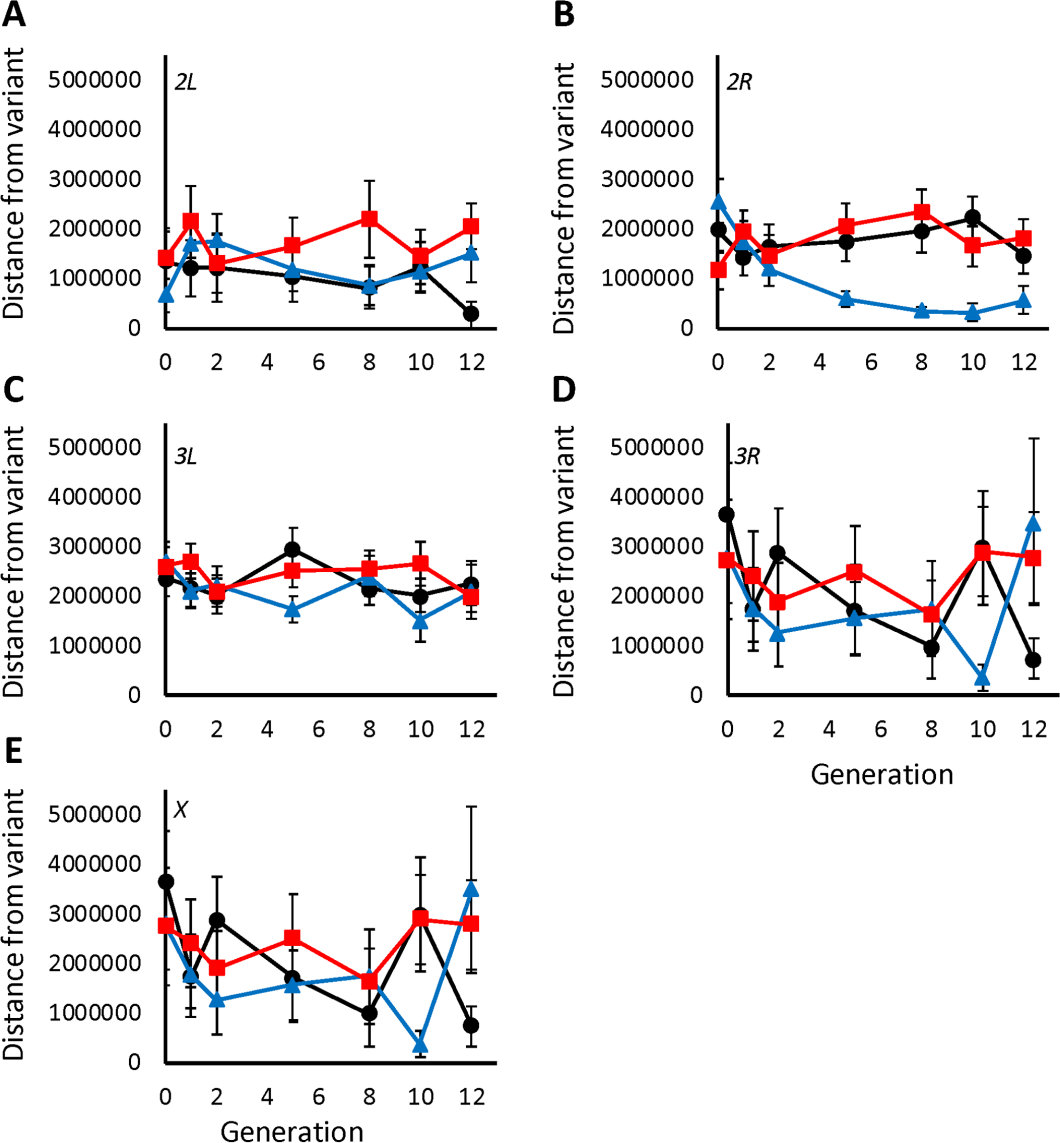
Change in distance between polymorphisms in LD over generation. The average distance between a given variant and a variant in high LD (*r*^2^ > 0.8) is shown for each generation. Dark blue triangles indicate averages over populations selected for long sleep; dark red squares indicate averages over populations selected for short sleep; and black circles indicate averages over control populations. (A) Chromosome *2L*. (B) Chromosome *2R*. (C) Chromosome *3L*. (D) Chromosome *3R*. (E) *X* chromosome.

The polymorphisms implicated in long and short sleep duration would be expected to have increased LD with selection, however, as nearby variants ‘hitchhike’ along with the focal variant. We therefore repeated the above procedure for the polymorphisms that were significant in the logistic regression and examined the changes across generations. 111 pairs out of a possible 7875 were in high LD in the 10 DGRP lines. We assessed the LD in each variant for each generation of selection as outlined above. We found that long-range LD (i.e., LD over distances greater than 1kb) did not decrease in the short- or long-sleeper populations; instead, the average distance between SNPs in high LD tended to remain relatively constant with the exception of long-sleeper variants on chromosome *2R* (Fig 8). Given these results and the relatively low number of generations of selection, we expected that some of the polymorphisms we identified were still in LD at generation 12. These putative LD blocks are indicated in S7 Table. The presence of these blocks suggested that only a subset of the identified variants were causal for changes in sleep duration.

### Comparison with sleep genome-wide association

The outbred population that we constructed used the 5 longest and 5 shortest sleeping lines of the DGRP, and represents the greatest amount of phenotypic variation in night sleep duration in the DGRP. We wondered whether artificial selection using this subset of lines would produce the same results as the previous genome-wide association study (GWAS) of sleep using 167 lines of the DGRP [18]. That study identified 160 polymorphisms associated with night sleep and 1,552 polymorphisms associated with night sleep *CV*_E_ with an FDR of 0.01 or less. In that study, night sleep duration and night sleep *CV*_E_ were highly genetically correlated, and 95.6% of the polymorphisms identified for night sleep overlapped with night sleep *CV*_E_ [18]. We found that none of the polymorphisms that we identified using logistic regression overlapped with those for night sleep duration in the GWAS [18], consistent with previous studies that have compared the results of advanced intercross population studies [91–93] or artificial selection [65] with GWAS. Greater overlap between the two studies occurred at the gene level, particularly if pleiotropic effects on other sleep traits were considered. Two genes, *Myb-interacting protein 120* (*mip120*) and *scribbled* (*scrib*), were implicated in night sleep duration in both studies, though only allele frequency changes in an intron of *scrib* exceeded the simulated drift threshold. Interestingly, 16 genes implicated in night sleep *CV*_E_ in the GWAS overlapped the present study and included *5-hydroxytryptamine (serotonin) receptor 1A (5-HT1A)*, *CG33158*, *CG34353*, *Dpr-interacting protein γ*, *faint sausage*, *Fish-lips*, *frizzled (fz)*, *Guanine nucleotide exchange factor in mesoderm*, *kin of irre* (*kirre*), *mip120*, *NK7*.*1*, *plum*, *scrib*, *still life*, *Synaptosomal-associated protein 25kDa*, and *Tie-like receptor tyrosine kinase*. Of these genes, only *CG34353*, *faint sausage*, *Fish-lips*, *fz*, *kirre*, *plum*, and *scrib* had allele frequency changes that exceeded the simulated drift threshold. If all sleep traits measured in the GWAS were considered, 34 genes overlapped with the logistic regression—15 if the drift threshold was considered (S8 Table). Alternatively, the lack of overlap among polymorphisms for mean night sleep between studies suggests a level of context specificity and complexity that might only be resolved in an analysis that combines the effects of polymorphisms across all loci simultaneously.

### Candidate genes and gene regions

**Fig 9:**
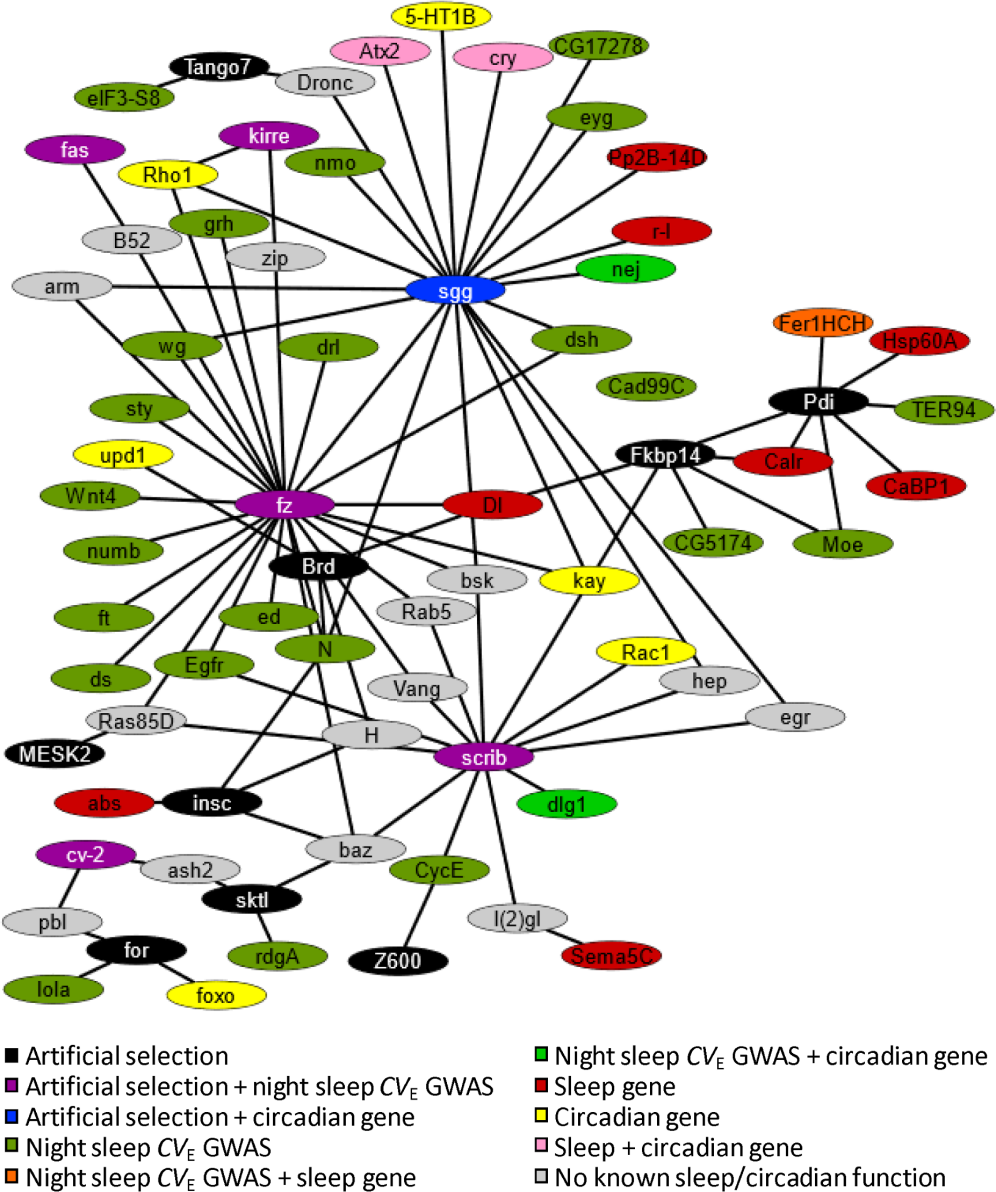
Network relationships between candidate genes from this study, the genome-wide association study, known sleep genes, and known circadian genes. The figure depicts a gene network based on known physical and genetic relationships among genes (S9 Table).

Of the 121 single nucleotide polymorphisms (SNPs) and 5 indels, 9 were in the 3’-UTR region of 8 genes, 2 were in the 5’-UTR of 2 genes, 11 were in the exon of 8 genes, 55 were in the intron of 46 genes, 17 were within 1 kb of 16 genes, and 32 were intergenic. Selection polymorphisms fell within the gene regions (± 1 kb of the coding region) of 80 candidate genes (S7 Table). We searched for known Gene Ontology (GO) relationships using DAVID [94] for all genes within 1kb of the putative selection polymorphisms. Genes were not enriched in GO categories, likely due to the relatively small number of candidate genes identified. However, we noted that several of the candidate genes mapped to well-known developmental and signaling pathways. *fz*, *shaggy* (*sgg*), and *dally* are components of the *Wnt* signaling pathway [95], and *sgg* is also part of the circadian rhythm pathway [96]; *pointed* has functions in the dorso-ventral axis formation (Egfr) [97] and MAPK [98] pathways; *scrib* functions in the Hippo signaling pathway [99]; and *skittles* and *small wing* are part of the phosphatidylinositol signaling pathway. Furthermore, the propensity for selection for the minor allele in short sleepers and the major allele in long sleepers suggests that certain patterns of alleles might function in a single pathway. We used Flybase [100] to mine previously published genetic and physical interactions with our candidate genes. We found that 38 of the genes could be connected by 364 genetic interactions and 312 physical interactions (S9 Table). If we reduced these genes to only those with allele frequency changes exceeding the simulated drift threshold, 15 could be drawn into a single network (Fig 9). This putative network revealed potential connections among the genes identified in this study and allele frequency shifts in long and short-sleeping populations. This network was extended to include published physical and genetic interactions of 45 candidate genes for night sleep and night sleep *CV*_E_ from the genome-wide association study of sleep using the DGRP [18] as well as 13 previously identified sleep [53, 71, 101–103] and 15 previously identified circadian genes [96, 104–116] (Fig 9; S9 and S10 Tables). This network forms a testable hypothesis for the interaction of candidate polymorphisms.

### Verification of candidate genes

**Fig 10:**
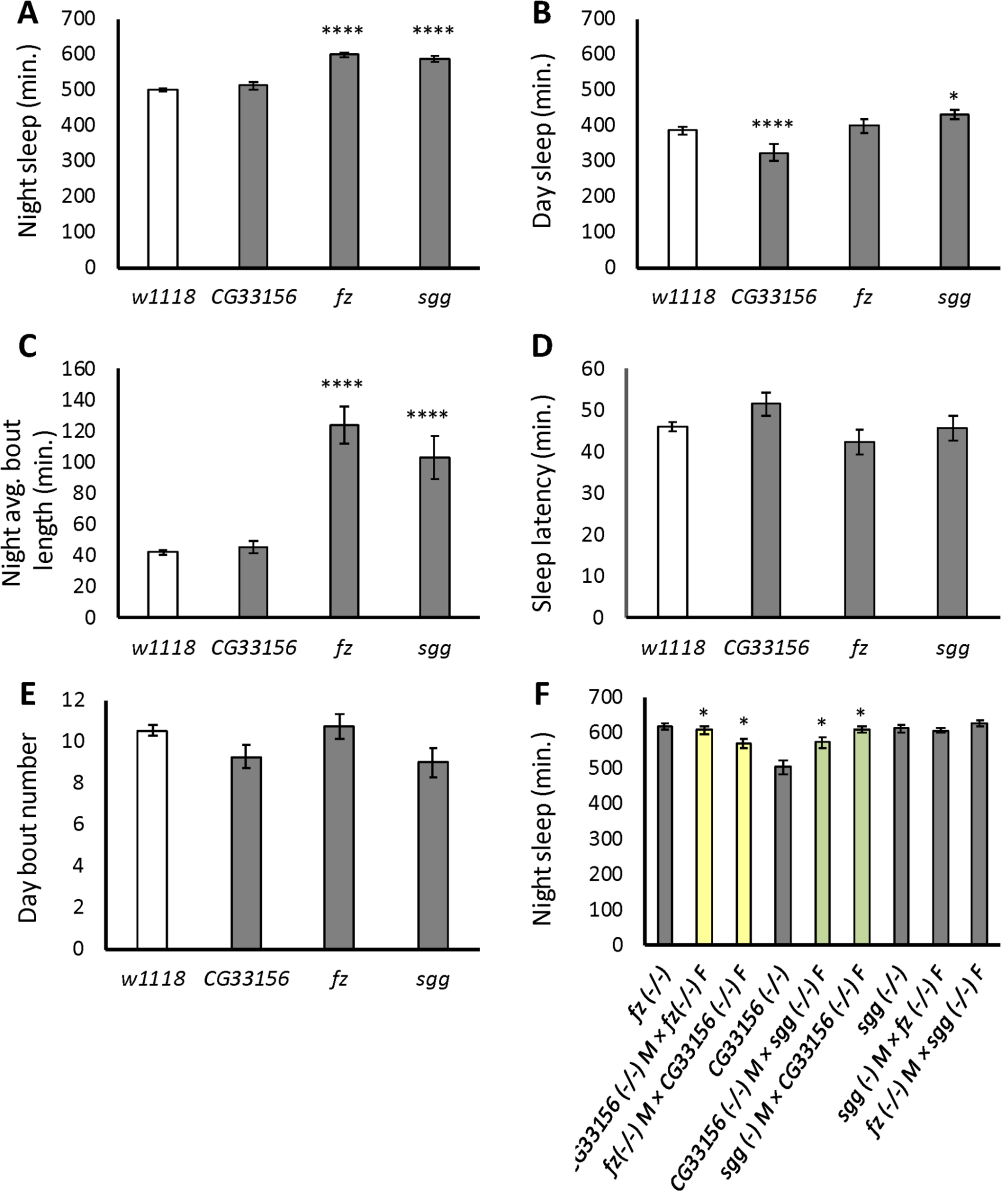
Effects of mutations on night sleep and correlated sleep traits. The figure shows the mean and standard error of sleep phenotypes. (A) Night sleep. (B) Day sleep. (C) Night average bout length. (D) Sleep latency. (E) Day bout number. (F) Night sleep trans-heterozygotes. Yellow bars indicate crosses with significant dominance and/or epistasis effects and maternal effects; green bars indicate crosses with significant maternal effects. M, male; F, female. *, *P* <0.05; **, *P* <0.01; ****, *P* <0.0001.

We tested the effect of *Minos* element insertions in three candidate genes from the logistic regression with allele frequency differences between long and short-sleeping populations that exceeded the simulated drift threshold (*CG33156*, *fz*, and *sgg*). We also tested three chromosomal deficiencies spanning candidate genes on chromosome *2R* for their effects on night sleep. We discuss each of these tests in turn. *CG33156* is a gene predicted to function as an NAD+ kinase due to its sequence similarity to yeast [100]. The *Minos* insertion in the exon region of *CG33156* did not affect night sleep, but did reduce day sleep in male and female flies by 61 minutes on average (*P* <0.0001) (Fig 10A and 10B; S11 Table). There was a corresponding 7.3-minute decrease in day average bout length (*P* = 0.0121), but no other effect on sleep (S6 Fig). *fz* is well-known as a receptor of *Wnt* ligands [95]. A *Minos* insertion in the second intron of *fz* increased night sleep by 100 minutes in males and females on average (*P* <0.0001) (Fig 10A). This insertion also had pleiotropic effects on night average bout length and night bout number, increasing the former by 82 minutes (*P* <0.0001) and reducing the latter by 6.7 bouts (*P* <0.0001) (Fig 10C and S6 Fig). *sgg* is a glycogen synthase kinase 3 with a known role in *Drosophila* circadian rhythms [96]. Male and female flies bearing a *Minos* insertion in an exon of *sgg* slept 86 minutes longer during the night than controls on average (*P* <0.0001) (Fig 10A; S11 Table). This insertion also increased day sleep by 47 minutes (*P* = 0.0206) (Fig 10B). In addition, flies with the *sgg* insertion had pleiotropic effects on night and day average bout length and night bout number, but no effect on sleep latency, day bout number, or waking activity (Fig 10C, 10D and 10E; S6 Fig; and S11 Table). Thus, our tests confirmed *fz* and *sgg* as candidate genes for night sleep duration, while *CG33156* had pleiotropic effects on other sleep traits.

To determine whether there were synergistic effects of these mutations on sleep, we tested full diallel crosses of the three *Minos* insertion lines. *sgg* is located on the *X* chromosome; thus, male progeny of crosses where the *Minos sgg* allele originates from male parent will not have the *sgg Minos* insertion. We therefore analyzed the effects of the trans-heterozygous genotypes on the sleep of males and females separately. We discuss here the effects of the trans-heterozygous genotypes on female sleep only, though all results are provided (Fig 10F, S12–S17 Tables). Highly significant effects of genotype on female night sleep were present (*P* <0.0001; S12 Table), as well as significant pleiotropic effects on all sleep traits (S12 Table). We further decomposed the genetic variance component into effects attributable to general combining ability (GCA), specific combining ability (SCA), and reciprocal cross effects (REC) [117]. GCA measures the effect of a given heterozygous mutation, expressed as a deviation from the mean phenotype of all heterozygotes. SCA is the deviation of trans-heterozygous genotypes from that of the additive combination of the heterozygotes, and reflects non-additive effects, i.e., dominance and epistasis [118]. REC indicates the degree to which the parent of origin of a mutation may alter sleep and can be thought of as maternal effects [117]. These three effects were highly significant in females for night sleep as well as all other sleep traits (S13 Table), indicating that sleep is influenced not only by additive effects, but by epistatic and maternal effects as well. Estimates of each of these effects and their standard errors for each cross are provided (GCA, S14 Table; SCA, S15 Table; REC, S16 Table; for mean phenotypes of all crosses, see S17 Table). For night sleep, general combining abilities were significantly different from zero for every gene in the female progeny, reflecting significant additive as well as additive-by-additive effects. Specific combining ability in night sleep was significant only for female progeny of the *sgg* males crossed with *CG33156* females, indicating dominance or epistatic interaction effects between these mutant alleles (*P* = 0.110) (S15 Table). Parental genotype also influenced night sleep; significant REC effects were seen on night sleep in female progeny when either *fz* or *sgg* were crossed to the *CG33156 Minos* insertion (*P* = 0.0133 and 0.0157, respectively). Though these mutations are in a genetic background distinct from that of the SAIP we used in the artificial selection protocol, we nevertheless observed effects on sleep not only in single-gene mutations, but epistatic and maternal effects among these candidate genes as well.

**Fig 11:**
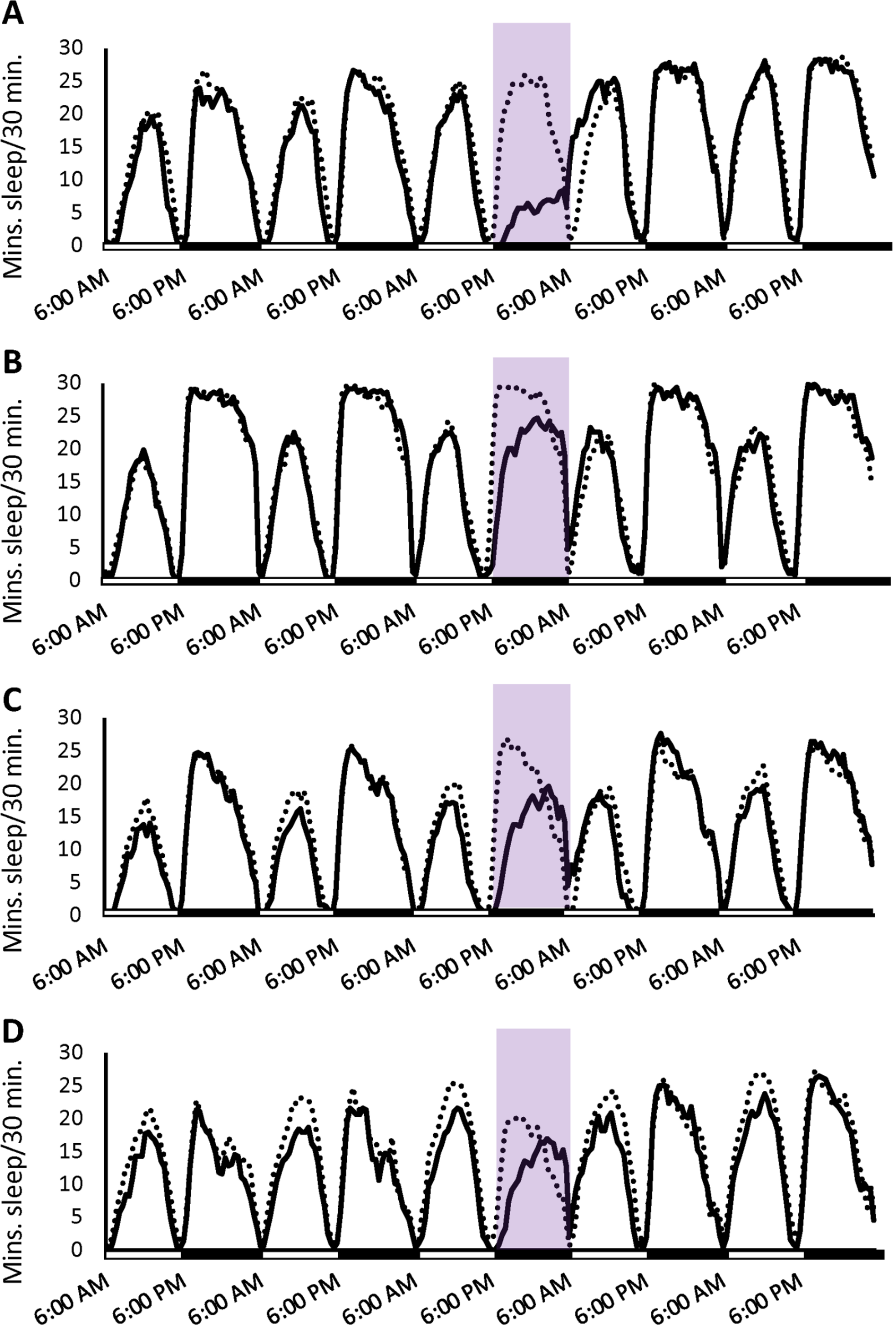
Sleep patterns of *Minos* mutants after 12 hours of sleep deprivation. Average minutes of sleep per 30 minutes are plotted for control (dotted lines) and deprived (solid lines) flies. Purple shading indicates the application of the shaking stimulus. Shaded bars along the x-axis indicate day (white) and night (black). (A) *sgg*. (B) *fz*. (C) *CG33156*. (D) *w*^1118^{5905}.

**Fig 12:**
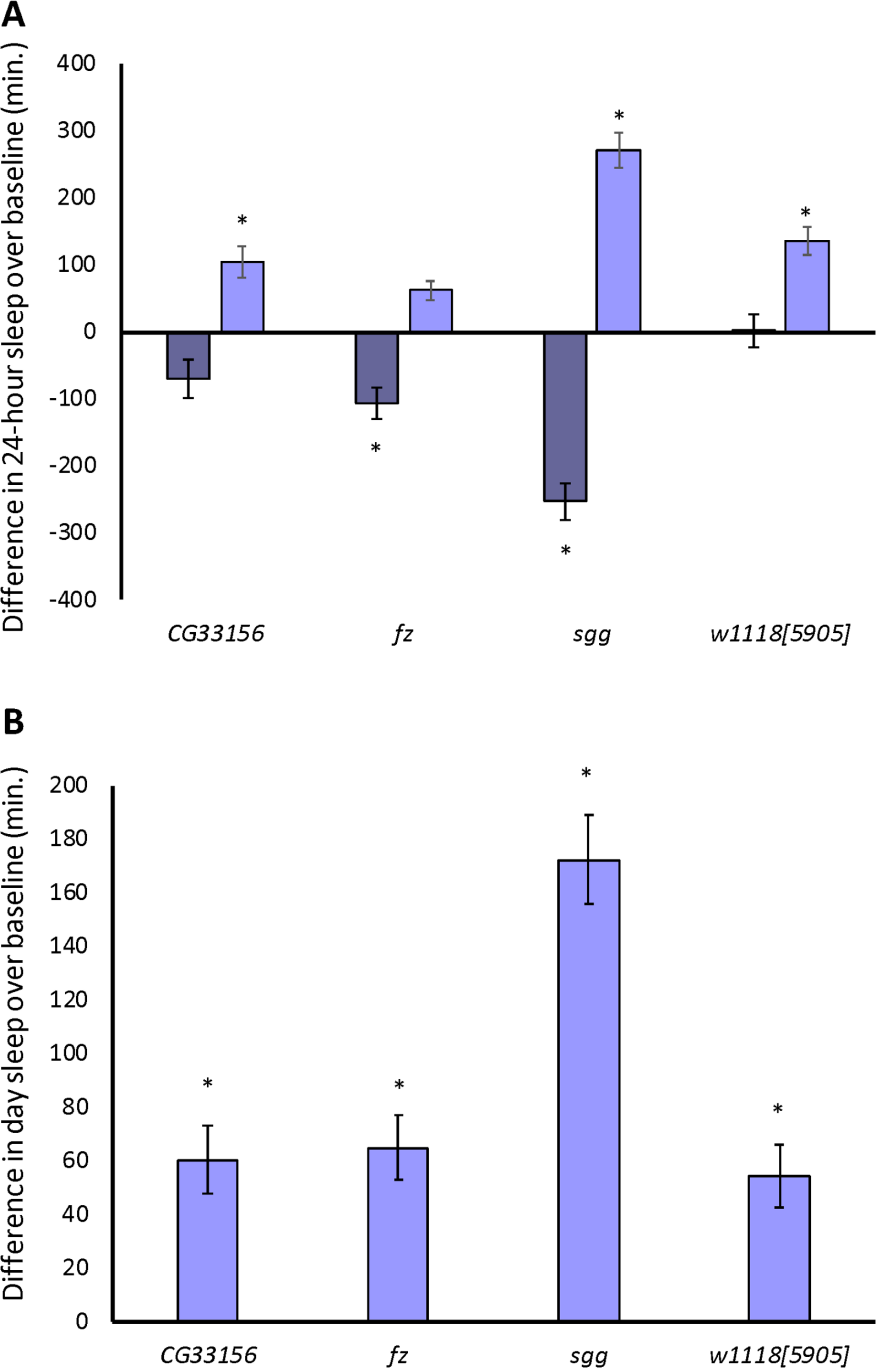
The response of *Minos* mutants to 12 hours of sleep deprivation. (A), the difference in 24-hour sleep from baseline (the average of days 1 and 2) sleep is plotted for day 3, when the mechanical stimulus was applied (dark blue bars) and for day 4, the recovery day (light blue bars). (B), the difference in day sleep from baseline for day 4, the recovery day. * *P* < 0.05; *P*-values reflect the comparison of deprived and/or recovery day sleep with baseline sleep.

In addition, we deprived these three *Minos* lines of 12 hours of night sleep, using the same protocol as for the selection populations. The sleep deprivation significantly affected night sleep in the three *Minos* lines and the control (all *P*_Treatment_ < 0.05). However, flies deprived in the isogenic control line had little night sleep differences over time as compared to their non-sleep-deprived counterparts (*P*_Treatment×Day_ = ns), suggesting that the stimulus was very mild (Fig 11A–11D and S7 Fig). The three mutants were more sensitive to the shaking stimulus (all *P*_Treatment×Day_ < 0.0001). *sgg*, *fz*, and *CG33156* had reductions in night sleep on day 3 that were greater than that of the control line (*P*_Treatment×Line_ < 0.0001, 0.0224, and 0.0446, respectively) (Fig 11A–11D; Fig 12), though the loss of sleep in *CG33156* was not statistically significant as compared to its own baseline sleep (Fig 12A). The *sgg* mutants were deprived of more night sleep and had thus had a larger daytime rebound, as well as increased sleep over the subsequent 24 hours (Fig 11A; Fig 12A and 12B). Likewise, day sleep in *fz* mutants was significantly different from baseline, but their 24-hour sleep did not increase (Fig 11B; Fig 12A and 12B). In addition, rebound sleep in *CG33156* was slightly increased over baseline 24-hour values (Fig 11C; Fig 12A and 12B). Adding to the difficulty in interpreting the behavior of these flies were monotonic increases in day and night sleep over time in the non-sleep-deprived controls (S7 Fig). In summary, we noted a significant increase in day sleep in all three mutants and the control on the recovery day in addition to a significant 24-hour rebound in all flies except *fz* mutants.

**Fig 13:**
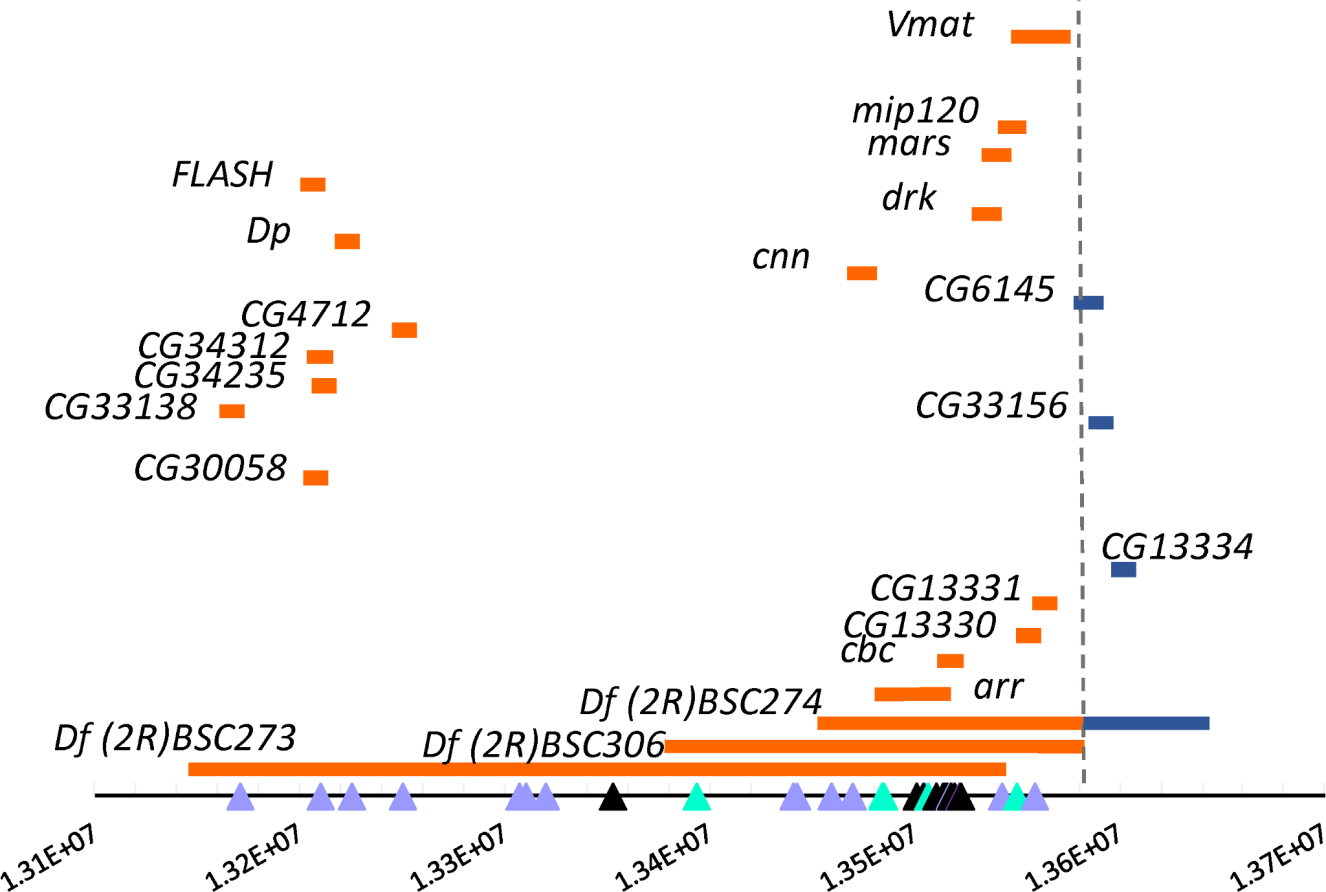
Deficiencies tested in the chromosome *2R* candidate region. The figure depicts genes implicated in this study and a previous genome-wide association study of sleep, plotted along the chromosome, along with tested deficiency lines. Not all genes in the region are plotted, and the plot is not to scale. Polymorphisms are indicated at the bottom as triangles. Lavender triangles, night sleep *CV*_E_; Purple triangles, night sleep from the GWAS; black triangles, both night sleep *CV*_E_ and night sleep; blue triangles, night sleep from the artificial selection study. Deficiency mapping implicated at least two quantitative trait loci (QTL) in the region. Blue bars denote genes within the first QTL; orange bars denote the second QTL.

Finally, we used three deficiencies to map a 99.6-kb region of chromosome *2R* that included polymorphisms for night sleep identified in this study and night sleep and night sleep *CV*_E_ in the GWAS (Fig 13; S18 Table). Deficiencies were crossed to the isogenic control line prior to sleep measures and compared to the control line. These hemizygous chromosome segments had mild but significant effects on night sleep. *Df(2R)BSC273* decreased night sleep by 15 minutes relative to controls (*P* = 0.0195); *Df(2R)BSC306* also decreased night sleep by 15 minutes relative to controls (*P* = 0.0336); and *Df(2R)BSC274* increased sleep by 13.2 minutes relative to controls (*P* = 0.0257). Thus, the mapping did not reveal causal candidate genes in the region, but did suggest opposing effects on night sleep between two regions along the chromosome. One region extended from 13159579 bp to 13539889 bp and had decreased sleep, while the other extended from 13539889 bp to 13593272 bp and increased sleep. Further fine mapping is required to test each of the candidate genes in this region, as quantitative trait loci spanning large genomic regions are likely to fractionate into many smaller candidate regions [119].

## Discussion

### Short sleep duration may be linked to deleterious fitness

Here we have used artificial selection to drive night sleep duration to extremely high and low levels. Our selection procedure also resulted in a positively correlated response to day sleep. This combined response produced mean 24-hour sleep times of 269 ± 21.11 and 176.59 ± 11.27 minutes in the short sleepers (replicate 1 and 2, respectively). This amount of sleep is comparable to short-sleeping flies with single mutations in *Sh*, *sss*, *inc*, and *nAChRα4* [44, 45, 50, 51], yet it was achieved using naturally occurring alleles. This finding suggests that flies with very short sleep could conceivably exist in nature. However, instances of flies with no sleep over the course of the 4-day assay were extremely rare. Of the 15,607 flies surviving the selection experiment, 152 (0.973%) had no night sleep, and only 15 (0.096%) of these flies had no sleep over four days. Even though we observed flies with no sleep over the four-day period, it seems unlikely that 24-hour sleep duration could be consistently driven to zero. First, our assay does not examine sleep patterns across the entire lifespan of the fly. Sleep patterns in very young or very old flies are markedly different, with long consolidated bouts of sleep in young flies and fragmented sleep in very old flies [10, 120]. Thus, it cannot be assumed that flies lacking sleep for four days would not sleep at all over their entire lifespan. Second, our activity-based assay does not account for the potential of localized sleep in the short sleepers, a phenomenon in which subsets of neurons display sleep-like properties while flies are awake [121]. Third, any natural selection pressure acting on night sleep would be expected to proceed in the direction of increased fitness. Consequently, artificial selection pressure would result in a greater phenotypic response in the direction of decreased fitness [65, 67]. We found that the response to selection was asymmetrical and stronger in the direction of reduced sleep, suggesting that short sleepers are less fit. Consistent with this finding, the short sleeper populations had increased sleep after 47 generations of relaxed selection, suggesting that very short sleep duration might be difficult to maintain in nature, except when strong selection pressure is present. We also noted that fewer short sleepers survived the sleep assay in later generations of selection, which may also be an indication that short sleepers are less fit. Alternatively, the lower survival rates may be an effect of inbreeding depression independent of sleep as survival rates were reduced in the control populations as well. Greater numbers of males from all selection populations survived the sleep assay as selection progressed, indicating that inbreeding effects may have a sex-specific basis [122]. However, both short and long sleepers did not have decreased lifespan, nor differences in egg-to-adult viability. Reduced lifespan is often seen in short-sleeping flies bearing a mutation in a single gene compared to controls [44, 45, 49, 51, 77, 78], though methodological differences such as measuring lifespan in the sleep monitor tubes [44, 77, 78], or using mated flies [45] may contribute to these differences. Additional work will be required to distinguish among these possibilities and to understand the relationship of sleep to fitness.

In long sleepers, mean 24-hour sleep times were 1073.25 ± 14.84 and 1060.43 ± 11.62 minutes (replicate 1 and 2, respectively). Only 40 flies (0.256%) had 720 minutes (12 hours) of night sleep. It seems unlikely that artificial selection could obtain 12 hours of mean night sleep, at least from the outbred starting population we created, because we were not able to increase the mean sleep above that of the longest-sleeping DGRP line. DGRP_335 averaged 688.76 minutes of night sleep [18], and night sleep in our long sleeper populations was 3.78 and 10.27 minutes (replicate 1 and 2, respectively) shy of this mean. Interestingly, the phenotypic variation in night sleep decayed very rapidly with selection for long sleep, which is another indication that the underlying genetic variation could not be pushed any further in the direction of long sleep.

### The sleep homeostat may be perturbed in selected populations

One of the hallmarks of sleep as a homeostatic process is the recovery sleep experienced after sleep is disturbed. Both long- and short-sleeping populations responded to a mild mechanical sleep deprivation stimulus, exhibiting decreased sleep during the stimulation procedure and increasing their sleep immediately afterwards. However, there was not an increase in sleep over the subsequent 24-hour period. Interestingly, *fz* mutants did not have increased sleep in the 24 hours after sleep deprivation either. There are several possible explanations for this pattern. First, the sleep homeostat may be disturbed in the selected populations. The disturbance is independent of the selection procedure since it was observed in all four selected populations. Alternatively, low or no rebound has been observed in genetically modified flies [123–127], indicating that sleep propensity can be partitioned from time spent awake [123, 124]. Periods of low sleep with little to no rebound can also occur in both flies and other species in nature under certain environmental conditions [32, 125, 126, 128–130], or in conjunction with reproductive behavior [33–36, 125]. Second, there may have been differences in the intensity of sleep among populations after the mechanical stimulus, which is an important characteristic of sleep [131] not measured in this study. Such differences in sleep intensity have been observed in flies, and they vary by genotype [132, 133]. Third, the mechanical stimulus we applied was relatively mild in comparison to other studies [47, 134]. We might have seen a greater response had a stronger stimulus been applied. In addition, sleep in the long sleeper replicate 1 population, and to a certain extent short sleepers of replicate 2, was disrupted after sleep deprivation. These flies had an increase of sleep during the day, and a drastic reduction of sleep at night. Overall, the sleep homeostat may be disrupted in the selected populations, and in two of the populations sleep deprivation impacted sleep patterns as well as the sleep homeostat.

### Selection for night sleep duration may have altered circadian rhythms

General patterns of sleep changed in both long and short sleepers. Previous work has shown that night average bout length is positively correlated with night sleep duration, while night bout number is negatively correlated with night sleep duration [18, 71]. Thus, as night sleep duration decreases, the average length of a nap is expected to decrease, while the number of naps are expected to increase, resulting in a more fragmented sleep pattern. Unexpectedly, however, both long and short sleepers exhibited a trend towards more consolidated sleep bouts. While the length of the average night sleep bout was reduced in the short sleepers and increased in the long sleepers, the number of night bouts for both selection schemes became progressively lower over time with respect to the unselected control. In addition to having more consolidated sleep bouts, the circadian timing of sleep became more variable in the short sleepers, even though they were being measured in standard LD conditions. Female flies adopted a more arrhythmic pattern of sleep while males became more nocturnal. Interestingly, one of the candidate polymorphisms we identified is located in the intronic region of *sgg*, a glycogen synthase kinase 3β (GSK-3β) that phosphorylates the canonical circadian clock gene *timeless* [96]. *sgg* overexpression reduces circadian period, while its reduction increases circadian period in flies [96]. *sgg* alleles exhibit a clinal distribution in North American flies, linking alleles of the gene with the forces of natural selection [60]. Here we demonstrated that *sgg* can alter sleep in flies in addition to its known function in circadian rhythms. Evidence from mouse studies suggests that GSK-3β may also have a role in mammalian sleep. Mice with increased GSK-3β enzymatic activity do not have changes in sleep duration, but instead have increased numbers of NREM and REM bouts as compared to wild-type mice [135]. In addition, the level of phosphorylated Ser9 in GSK-3β protein increases in the hippocampus of spontaneously waking versus sleeping rats [136]. Thus, *sgg* may have a conserved role in sleep as well as circadian rhythms.

### Genome-wide studies reveal that different underlying genotypes lead to similar phenotypes

Using the CMH test, we observed genome-wide allele frequency changes in the long and short sleeper populations across just 13 generations of selection, making it a challenge to pinpoint the true targets of selection. Remarkably, large numbers of changes also occurred in the control populations, which did not undergo any selection. We therefore assumed that any significant allele frequency changes in the control population were due to random genetic drift, laboratory adaptation, or potential adaptation to the admixture of genomes used to construct the SAIP, and removed them from further consideration. This does not mean that these polymorphisms are not relevant to sleep, however; they were simply indistinguishable from other potential causes in this experiment. Also, few polymorphisms with significant allele frequency changes via the CMH test were common to replicates within each selection scheme despite the similarities in night sleep duration phenotypes. Thus, different constellations of alleles may produce similar phenotypes, as previously observed in a comparison of replicate selection populations adapting to a novel environment [56]; alternatively, the vast majority of alleles may have nothing to do with sleep and are randomly drifting, as might be anticipated given the changes we observed in the control populations. Here we focused on allele frequency changes common to all replicates of both long and short sleeper populations over all seven sequenced generations simultaneously as the most likely targets of selection. In addition, we could further refine the number of target variants to 59 by simulating the greatest allele frequency changes that could be expected to be due to drift. This was an effective strategy for reducing the total number of potential selection targets. Our estimates of linkage disequilibrium strongly suggest that many of the polymorphisms we identified are not causal for long or short sleep but are in fact in linkage disequilibrium (LD) with the causal SNP [137]. Interestingly, the estimates of LD show persistent long-range LD, which has been observed in the DGRP and other wild-derived populations previously. Some evidence for this is present in the genomic region surrounding *mip120* on chromosome *2R*. Polymorphisms along a 99.6-kb region of chromosome *2R* were implicated in either this study or the GWAS [18] and include *arrow* (*Wnt* signaling pathway), *crowded by cid*, *downstream of receptor kinase* (MAPK pathway), *mars*, *Vesicular monamine transporter (Vmat)*, *CG13330*, *CG13331*, and *CG33156*. Portions of this region are in LD in the DGRP [18], and *Vmat* has already been shown to be relevant to sleep [18, 138]. Deficiency mapping of this region could not eliminate any of these genes as potential candidates. In addition, many of the variants we identified on chromosome *3L* were also estimated to be in high LD. Mapping at higher resolution will be required to determine the causal genes.

The allele frequency distribution of the significant polymorphisms identified for night sleep duration in the GWA study differs from the original distribution of these alleles in the DGRP. The average minor allele frequency of a night sleep duration polymorphism in that study was 0.088. At generation 0, these polymorphisms were far more common in the outbred population constructed in this experiment, with an average minor allele frequency of 0.232. Yet it was a different group of polymorphisms, also at a relatively high average minor allele frequency (0.337) in generation 0, that are the putative targets of artificial selection. Both groups of candidate polymorphisms have some similarities, however. Most of the candidate polymorphisms identified in the present study were selected in the direction of the minor allele for short-sleepers [97], and towards the major allele for long sleepers [91]. We observed the same pattern in the GWAS: the minor alleles of candidate polymorphisms were more often associated with short sleep, while the major alleles were associated with long sleep [18]. Furthermore, a correlated response to artificial selection for short sleep was the increased coefficient of environmental variation (*CV*_E_), reflected by the slow decay of variability in sleep among individuals over time. In contrast, the variability in sleep among individuals from populations selected for long sleep decreased rapidly, greatly reducing *CV*_E_. This pattern was also observed in the GWA study [18]. One of the consequences of short sleep, then, is increased sensitivity to small environmental perturbations, heightening arousal. This variability sets up a scenario in which low-frequency deleterious alleles might be maintained in the face of natural selection for sleep [18]. Alternatively, negative impacts on fitness might be reduced if the alleles are assembled in the fashion we have seen in this experiment, i.e., all of the minor alleles in one group, and all of the major alleles in the other.

### Artificial selection using natural variation leads to candidate networks for sleep duration

As previous experiments in olfaction, starvation resistance, startle response, chill coma recovery, and food intake have suggested, the genetic overlap of natural population-based studies is neither single nucleotides nor single genes, but instead occurs among genetic pathways [65, 91, 92]. We identified 80 candidate genes implicated in extreme long or short sleep. Two of these genes, *mip120* and *scrib*, were implicated in the GWAS for night sleep, and an additional 16 were associated with night sleep *CV*_E_ [18]. Mutations or knockdown in several of these candidate genes in flies have been previously shown to affect night sleep (*foraging* and *fz*) [18, 139], sleep homeostasis (*heimdall*) [53], or 24-hour sleep duration (*5-HT1A*, *foraging*, *Calreticulin*, and *Semaphorin 5C*) [71, 139, 140]. Genes involved in several developmental and signaling pathways were implicated: dorso-ventral axis formation (EGFR), Wnt signaling, MAPK signaling, Hippo, phosphatidylinositol signaling, and circadian rhythms. Components of these pathways have been implicated in sleep and circadian rhythms. Previous work has shown that increases in EGFR signaling increases sleep duration in flies [141] and rabbits [142]. Wnt signaling components were previously implicated in the GWAS of sleep in flies [18], and were recently noted in a human study of changes in methylation during sleep deprivation [143]. The MAP kinase pathway has a role in circadian signaling in flies [144] and mammals [145]. Mutations in components of the circadian rhythm pathway have altered sleep as well [42, 43, 73]. Here we verified that *CG33156*, *fz*, and *sgg* are candidate genes for night sleep using mutations. Our results measuring sleep in trans-heterozygous crosses among these mutations demonstrate that sleep can be altered by dominance, epistatic, and maternal effects. Future functional validation is needed to validate the role of the remaining genes in sleep and their relationship to each other in the network. If this network of highly conserved genes indeed represents a core sleep network, one might predict that allele frequency shifts in this network will 1) underlie pleiotropic responses to changes in environmental conditions [146] and 2) be relevant to sleep in other species. More broadly, the study of naturally-occurring genetic variation as implemented here can be manipulated to gain insights on the genetic basis of complex physiological traits and disease.

## Materials and methods

### Construction of outbred population

We constructed an outbred population of flies using ten lines from the *Drosophila* Genetic Reference Panel (DGRP) [80, 81] with the most extreme night sleep phenotypes for both sexes combined. Five lines had the shortest average night sleep for both males and females in the population: DGRP_38, DGRP_310, DGRP_365, DGRP_808, and DGRP_832 [18]. The other five lines had the longest average night sleep in the population: DGRP_235, DGRP_313, DGRP_335, DGRP_338, and DGRP_379 [18]. All ten lines were crossed in a full diallel design, resulting in 100 crosses. Two virgin females and two males from the F1 of each cross were then randomly mixed together and placed into 20 bottles, with 10 males and 10 females in each bottle. At each generation thereafter, 20 virgin females and 20 males from each bottle were randomly mixed with the other flies, and a new generation of 20 bottles was reared. Each generation of random mating had a census population size of 800. The genomes of these flies were allowed to recombine in this manner for 21 generations, resulting in an advanced intercross population of flies we named the Sleep Advanced Intercross Population (SAIP).

### Sleep assays and phenotyping

Flies were maintained and assayed on standard food (http://flystocks.bio.indiana.edu/Fly_Work/media-recipes/bloomfood.htm) in constant temperature (25°C), humidity (60%), and light:dark cycle (12hr:12hr). To measure sleep, we collected male and female virgins from each population. Virgins were maintained in single-sex vials of 20 flies for four days before the experiment began (i.e., five vials per sex per population) to control for any potential effects on sleep of mating status [125] and social exposure [147]. Individual flies were loaded into Drosophila Activity Monitors (DAM2, Trikinetics, Waltham, MA) and sleep and activity parameters were recorded for five days. The monitors use an infra-red beam to detect activity counts in individual flies as they move past it; five minutes without an activity count is defined as sleep [42, 43]. To mitigate the effects of CO_2_ anesthesia or any other potential acclimation effects, the first day of data recording was discarded. Flies were visually examined after the sleep and activity recordings were completed; data from any flies that did not survive the recording period was discarded. Sleep Analysis v6-1 software (R. S. Barnes, personal communication) was used to calculate night and day sleep duration in minutes, night and day sleep bout number, and night and day average sleep bout length; it also calculated sleep latency, the time in minutes to the first sleep bout after lights are turned off and waking activity, the number of activity counts per minute spent awake. In addition, we calculated the coefficient of environmental variation (*CV*_E_) for each sleep trait as (*σ*_E_/*μ*)×100 [148], where *σ*_E_ is the within-replicate population standard deviation, and *μ* is the within-replicate mean. We used an in-house C# program (R. S. Barnes, personal communication) to partition the activity counts for each fly as asleep, awake, or in a transient pause (1 to 4 minutes of inactivity). We used this information to calculate the percentage of flies in each state over time.

### Quantitative genetic analysis of outbred population prior to selection

At Generation 0 of the selection procedure, we divided the outbred population into six populations by seeding six bottles with 25 randomly chosen flies of each sex. Two populations served as replicate unselected controls (C1 and C2), two populations were selected for long night sleep (L1 and L2), and two populations were selected for short night sleep (S1 and S2). We measured sleep in 100 males and 100 females from each of these populations prior to initiating the artificial selection procedure. We calculated differences in sleep among these populations using an ANOVA model (see Quantitative genetics of selection below for the model). The resulting analyses indicated that there were no significant differences in any sleep trait among these populations, with the exception of night bout number (S1 Fig; Generation 0 of S2 Table). Thus, each population had essentially the same phenotypic characteristics prior to selection.

### Artificial selection procedure

We implemented the following artificial selection procedure each generation. First, 100 virgins of each sex were collected from each population bottle and placed into the sleep monitors. Sleep and activity were monitored continuously over a five-day period. Second, we calculated all sleep parameters for every fly in each selection population. For the four selection populations (L1, L2, S1, and S2), the 25 males and females with the most extreme (high or low) night sleep within each population were chosen as parents for the next generation of that population. For the control populations (C1 and C2), we chose 25 males and 25 females at random to be the parents for the next generation of each population. We repeated this procedure for 13 generations. After generation 13, the six populations were maintained by seeding culture bottles with 25 randomly chosen flies of each sex.

### Sleep deprivation protocol

We subjected artificially-selected flies to sleep deprivation at Generation 60 using the following procedure. Individual flies were loaded into the DAM2 Trikinetics monitors using the protocol above. After allowing the flies to acclimate in the monitors for one day, we recorded normal sleep and activity for two days. We deprived the flies of sleep on the night of the third day using a standard vortexer (Troemner, Thorofare, NJ) with a custom mounting plate (Trikinetics, Waltham, MA). The vortexer gently shook (speed = 2.0) the flies for five seconds every minute of the 12-hour night sleep period. We allowed the flies to recover for two additional days. An identical set of flies was placed into Trikinetics monitors on a different shelf in the same incubator to serve as a non-sleep-deprived control. We repeated these experiments two times. We used this protocol to deprive the long- (L1 and L2) and short-sleeper (S1 and S2) populations. We also applied this protocol to deprive the *Minos* element lines (see below) of sleep. We used an in-house Python program to calculate the number of flies asleep during any given minute, and to compute the average amount asleep per 30 minutes. We examined the overall efficacy of the sleep deprivation protocol for each population/line separately using the ANOVA model *Y* = *µ* + *T* + *D* + *S* + *T*×*D* + *T*×*S* + *D*×*S* + *T*×*D*×*S* + *ε*, where *T* is the control or sleep deprived treatment, *D* is day, and *S* is sex. To determine whether night and/or day sleep changed across days for the sleep-deprived group and their respective controls, we analyzed the data for each population/line separately using the reduced ANOVA model *Y* = *μ* + *D* + *S* + *D*×*S* + *ε*, where *D* and *S* are as defined above. Post-hoc Tukey analysis was used to determine which days had significant changes in sleep duration. In addition, to assess sleep deprivation in the *Minos* lines, we assessed differences between mutants and controls per day using the model *Y* = *μ* + *T* + *L* + *S* + *T*×*L* + *T*×*S* + *S*×*L* + *T*×*L*×*S* + *ε*, where *T* and *S* are as defined above and *L* is Line.

### Lifespan and egg-to-adult viability assay

We evaluated two different life-history traits in the selected and control populations of flies. At generation 14, flies were assayed for lifespan. 100 male and female virgins of each population were placed into 10 replicate vials per sex per population, with 10 same-sex flies per vial. Every two days, the flies were tipped into vials containing fresh food, and deaths were recorded until all flies were dead. Flies were assayed for egg-to-adult viability for nine generations, beginning at generation 4 and continuing through generation 12. Two replicate fly cultures per population per generation were seeded with 25 flies of each sex. Parents were cleared from the vials after 3 days, and the numbers of adult flies emerging were counted from days 10–14.

### Quantitative genetic analysis of selection

To evaluate the effectiveness of the selection procedure, we used the equation *Y* = *μ* + *Sel* + *Rep*(*Sel*) + *Sex* + *Gen* + *Sel*×*Sex* + *Sel*×*Gen* + *Rep*(*Sel*)×*Sex* + *Rep*(*Sel*)×*Gen* + *Sex*×*Gen* + *Sel*×*Sex*×*Gen* + *Rep*(*Sel*)×*Sex*×*Gen* + *ε*, where *Y* is night sleep duration, *Sel* is the selection procedure (Short or Long night sleep); *Gen* is the fixed effect of generation; *Rep* is the random effect of replicate population; and *ε* is the variance within populations [68]. A significant (*P* < 0.05) *Sel* term indicates that the selection procedure was effective, and a significant *Sel*×*Sex* term reveals sex-specific responses to selection. A significant *Sel*×*Gen* (*Sel*×*Sex*×*Gen*) term implies that the response to selection differed across generations (and sexes). We used the same model to determine whether the other sleep parameters and egg-to-adult viability had a correlated response to selection for night sleep. To further characterize sex- and generation-specific responses to selection for night sleep, we used the reduced model *Y* = *μ* + *Sel* + *Rep*(*Sel*) + *Gen* + *Sel*×*Gen* + *Rep*(*Sel*)×*Gen* + *ε* to evaluate sleep characteristics in each sex separately; and the reduced models *Y* = *μ* + *Sel* + *Rep*(*Sel*) + *Sex* + *Sel*×*Sex* + *Rep*(*Sel*)×*Sex* + *ε* and *Y* = *μ* + *Sel* + *Rep*(*Sel*) + *ε* to analyze the response to selection in each generation separately and for each generation in each sex separately, respectively. The model *Y* = *μ* + *Sel* + *Rep*(*Sel*) + *Sex* + *Sel*×*Sex* + *Rep*(*Sel*)×*Sex* + *ε* was used for the lifespan analysis. All analyses were implemented using SAS (Cary, NC). To assess the response of night sleep to selection, we used the breeder’s equation Σ*R* = *h*^2^Σ*S* to calculate the realized heritability *h*^2^, where Σ*R* is the cumulated response to selection, calculated as the mean night sleep of the offspring minus the mean night sleep of the parental generation; and Σ*S* is the cumulated selection differential, calculated as the mean night sleep of the selected parents minus the mean night sleep of the parental generation [67]. The same equation was used to assess changes in night sleep in the unselected control, except that the cumulated response is more properly termed cumulative difference, and the slope is the coefficient of the regression rather than the realized heritability.

### DNA extraction of multi-fly samples

For each generation of selection, we flash-froze 35 flies of each sex from each of the selection and control populations. We extracted DNA from these flies using a cell lysis solution {1.58 g of Tris-HCl (Quality Biological, Gaithersburg, MD), 37.22 g EDTA disodium salt (Quality Biological, Gaithersburg, MD) and filled to 1 liter with RNase/DNase-free water, adjusting the pH to 8.0 with 10 M NaOH (Sigma Aldrich, St. Louis, MO) when necessary}. Flies were homogenized in 300 μl of cell lysis solution and four to six 2.38 mm metal beads (Omni International, Kennesaw, GA) using an Omni Bead Ruptor {5 cycles of 5 seconds ON/ 5 seconds OFF—Setting 5 m/s, equal to 3100 rpm (Omni International, Kennesaw, GA)}. An additional 400 μl of cell lysis solution, 120 μl of 10% SDS (Invitrogen, Grand Island, NY), and 60 μl of Proteinase K (Life Technologies, Grand Island, NY) were added and the samples were incubated at 65°C for 1 hour. The lysate was then transferred to a clean 1.5 ml tube, leaving the beads behind. RNA was eliminated from the lysate by adding 3.5 μl RNase A (20 mg/ml) (Life Technologies, Grand Island, NY), mixing, and incubating at 37°C for 15 minutes. Protein precipitation was initiated by adding 200 μl of Ammonium Acetate (Quality Biological, Gaithersburg, MD) solution (289.1 g of Ammonium Acetate in 500 ml of RNase/DNase-free water) to tubes chilled on ice for 5 minutes. Samples were vortexed at high speed for 20 seconds to mix, incubated on ice for 15 minutes, and then centrifuged at maximum speed for 5 minutes. The supernatant was then transferred to a clean 1.5 ml tube, and 3 μl of glycogen (Qiagen, Valencia, CA) was added. 700 μl of 100% isopropanol (VWR International, Radnor, PA) was added and mixed to precipitate the DNA; samples were incubated for 1 hour at -20°C. The samples were then centrifuged at maximum speed for 5 minutes. The supernatant was poured off and the DNA pellet was washed with 600 μl of 75% ethanol (NIH Supply Center, Gaithersburg, MD), then centrifuged at maximum speed for 5 minutes. The ethanol was poured off and the pellet air-dried for 15 minutes. Samples were re-hydrated in 120 μl of RNase/DNase-free water.

DNA samples were then purified using a phenol-chloroform extraction. First, 80 μl of 10 mM Tris (Quality Biological, Gaithersburg, MD), 1 mM EDTA, pH 7.8 and 200 μl of fresh phenol:chloroform:isoamyl alcohol (25:24:1) (Sigma Aldrich, St. Louis, MO) were added to the DNA sample. The samples were vortexed for 30 seconds, then centrifuged at maximum speed in a 4°C centrifuge for 5 minutes. The resulting upper layer (~ 170 μl) was transferred to a fresh 1.5-ml tube. An equal volume of chloroform (NIH Supply Center, Gaithersburg, MD) (200 μl) was added. The sample was vortexed and centrifuged at maximum speed in a 4°C centrifuge for 5 minutes. The resulting upper layer (~ 150 μl) was transferred to a fresh 1.5-ml tube. Next, 20 μl of sodium acetate (NaOAc) (Sigma Aldrich, St. Louis, MO), 500 μl of pure ethanol, and 1 μl glycogen were added. The sample was mixed and placed in ice for 15 minutes. Afterwards, the samples were centrifuged at maximum speed in a 4°C centrifuge for 30 minutes. The supernatant was removed, and the pellet washed with 0.5 ml of 70% ethanol. The sample was centrifuged at maximum speed for 5 minutes. The supernatant was removed and the sample air-dried for 5 minutes. The resulting DNA pellet was dissolved in 25 μl sterile 10 mM Tris, 0.1 mM EDTA, pH 7.8 and heated for 2 minutes at 55°C. DNA quantity was measured and quality evaluated (260/280 ratios of 1.8 or greater) using the Nanodrop 8000 (Thermo Fisher Scientific, Asheville, NC).

### DNA library preparation and sequencing

We sequenced DNA from each selection population and sex for the following generations: 0, 1, 2, 5, 8, 10, and 12. One microgram of genomic DNA was sheared to ~350 bp using a Covaris E210 (Covaris, Woburn, MA) with settings: duty cycle 10%; intensity 5; cycles/burst 200; time 45s. Libraries were constructed using the Tru-Seq DNA PCR-Free LT Sample Prep Kit (Illumina, San Diego, CA) according to the manufacturer’s protocol. The libraries were pooled in groups of 24 and run on a HiSeq2500 (Illumina, San Diego, CA) with version 3 sequencing reagents to generate a minimum of 37 million paired-end 126 base reads per library. The HiSeq data was processed using RTA1.18.64 and CASAVA 1.8.2.

### Sequence alignment and SNP calls

Sequences were aligned and mapped to the *D*. *melanogaster* assembly BDGP Release 6, UCSC version dm6 using with Burrows-Wheeler Alignment-MEM version 0.7.12 [149] and Novoalign version 3.02.07 (Novocraft Technologies, Selangor, Malaysia), using the -t 400 option to optimize alignment speed. We used previously identified informative SNPs and indels in Freeze 2.0 of the DGRP [81], after converting the coordinates to the Release 6 assembly [150]. Reads were realigned around indels from this set of polymorphisms with GATK version 2.8.1 [151]. Duplicate reads were removed with samtools version 0.1.18 after alignment, merging, and sorting of the reads. Novel variants were discovered using LoFreq version 2.1.2 [82] with default parameters. We retained variants called by LoFreq that occurred in 14 or more samples. Allele counts for all single nucleotide variant sites (both known DGRP Freeze 2.0 and novel LoFreq polymorphisms) were determined using the ‘bamcounts’ command of the bardCNV package (http://github.com/nhansen/BardCNV) with the option -minqual 20 to filter reads for a minimum phred quality of 20. Counts for reads spanning indels were performed by first widening indel variants to their narrowest unambiguous region, then tallying spanning reads both with and without the indel using the perl module Bio::SamTools (http://search.cpan.org/~lds/Bio-SamTools/lib/Bio/DB/Sam.pm). Confidence interval boundaries with the highest posterior density for the estimated read allele proportions were calculated in R using CRAN ‘binom’ package’s ‘binom.bayes’ function. For downstream analyses, we considered all bialleleic polymorphisms with total coverage of 10 and less than 2000 in each population [56].

### Detection of allele frequency changes

We used the Cochran-Mantel-Haenszel (CMH) Test to detect changes in allele frequency for all known segregating 2,222,264 and putative de novo 258,268 polymorphisms [83] between generations 0–1, 1–2, 2–5, 5–8, 8–10, and 10–12. This test is unbiased, easy to apply, and has been previously used to identify candidate polymorphisms in evolve and re-sequence studies [56, 59]. We removed polymorphisms with less than 10 total read counts or more than 2000 total read counts from the analysis. We used a Bonferroni-corrected *P*-value (2.3 × 10^−8^) as the threshold of significance for the CMH tests in order to account for multiple tests. We made this calculation for both sexes combined as sleep in both sexes responded to the selection procedure (*P*_sel×sex_ = 0.1501, S1 Table). Despite the similarity in phenotypic changes among replicate long- or short-sleeper populations, few polymorphisms with significant allele frequency changes overlapped between replicate populations. However, significant CMH tests cannot distinguish among allele frequency changes that are responsible for the sleep phenotypes we observed, and changes due to other forces that might alter allele frequency. For example, large numbers of frequency changes were potentially due to random genetic drift or laboratory adaptation, as the numbers of significant polymorphisms in the control populations indicate. Nor can the CMH test account for differences in allele counts that arise due to differences in sequencing coverage [59, 65], sampling bias, or sequencing error. We examined the overlap between candidate polymorphisms that were significant for the CMH test in the control population with those in the selected populations. We eliminated polymorphisms that overlapped with the control populations as being indistinguishable from random genetic drift. This left 69,188 polymorphisms that overlapped between the long- and short-sleeping populations. We then applied a logistic regression model to the read count data of the form: *Y* = *β*_0_ + *β*_1_*Gen* + *β*_2_*Sel* + *β*_3_*Gen*×*Sel*, where *Y* is allele type (major or minor), *Gen* is generation, and *Sel* is selection scheme (Long or Short). Both long and short selection population replicates for all seven generations and both sexes were put into the logistic regression analysis. We required that the *P*-value for the lack of fit be ≥ 0.05 (higher *P*-value indicates that there is no evidence to reject the null hypothesis that the model fits); the *P*-value for *Sel* to be ≤ 0.05, and the *P*-value for *Gen*×*Sel* to be ≤ 0.05 [152]. All analyses were implemented using SAS (Cary, NC).

### Linkage disequilibrium estimation

We chose 3,600 SNPs at random per chromosome arm and calculated linkage disequilibrium among these SNPs for the 10 DGRP lines using plink 1.0.7 [153]. To estimate LD between SNPs in the SAIP and resulting artificial selection populations, we used the following procedure. For each SNP pair, we designated one SNP as SNP A and the other as SNP B. We binned the minor allele frequency of SNP A, *p*_a_, in 0.01 increments from 0.01 to 0.5. We then calculated the lower and upper bounds of the corresponding minor allele frequency at SNP B, *p*_b_, that would result in an *r*^2^ of 0.8 or greater using the following formulations from VanLiere *et al*. [89]:

The lower bound of *p*_b_ is *r*^2^*p*_a_/(1 + *r*^2^*p*_a_−*p*_a_).

The upper bound of *p*_b_ is the minimum of *p*_a_/(*r*^2^ –*r*^2^*p*_a_ + *p*_a_), or 0.5.

We determined whether the actual minor allele frequency at SNP B was within the upper and lower bounds given by these two equations. If it was, we assumed that SNP B was in high LD (i.e., *r*^2^ ≥ 0.8) with SNP A.

In addition, we used the *Drosophila melanogaster* Recombination Rate Calculator (http://petrov.stanford.edu/RRcalculator.html) to estimate recombination rates for the 126 variants we identified using logistic regression. Estimates based on Marey map equations [154] and on empirical observations of recombination [86] are included in S7 Table.

### Drift simulations

We compared the actual allele frequency changes in the control populations with simulated random genetic drift. We first estimated the variance effective population size, *N*_e_, of our populations as

$$N_{e}=\frac{-t}{2ln(1-\frac{\sigma^{2}}{q_{0}(1-q_{0})})}$$

where *t* is the time in generations, *q*_0_ is the starting allele frequency at generation 0, and *σ*^2^ is the variance in allele frequencies [155]. Using the SNPs that had significant CMH tests in the control populations (i.e., those SNPs presumed to be drifting), we binned the allele frequencies at generation 0 from 0.05–0.50. We then calculated the variance in allele frequency across SNPs in the control populations at generation 12 [65]. We estimated *N*_e_ using the equation above for each allele frequency bin at Generation 12. The median *N*_e_ was 42.5 (21 males and 21 females) which we applied to the drift simulations. Drift was simulated for a single SNP by random and independent sampling of parental genotypes over 12 generations for a given starting minor allele frequency [65]. We simulated drift using starting minor allele frequencies that ranged from 0.01 to 0.5 in 0.01-frequency increments. Each simulation for a given starting minor allele frequency was repeated 10,000 times. The entire process was replicated 2 times for the autosomes and 2 times for the *X* chromosome, to represent the long-sleeper and short-sleeper populations with replication. We then calculated the absolute value of the average difference between short and long sleeper populations for each starting minor allele frequency. The 99.9% quantile was used as an upper bound of the potential allele frequency change due to drift for each starting minor allele frequency (65).

### Verification of candidate genes

We tested *Minos* element *Mi{ET1}* insertion lines [156] in three candidate genes: *CG33156*, *fz*, and *sgg*. The alleles tested were *CG33156*^MB05931^, *fz*^MB07478^, and *sgg*^MB03827^ (Bloomington, IN stock center). The *Mi{ET1}CG33156*^MB05931^ insertion is located on chromosome *2R* at position 13,549,134, within an exon common to all 5 isoforms of *CG33156*. The *Mi{ET1}fz*^MB07478^ insertion is located at position 14,303,880 on chromosome *3L* in the second intron of both isoforms of *fz*. The *Mi*{*ET1*}*sgg*^MB03827^ insertion is located at position 2,673,583 on the *X* chromosome; this insertion is in an exon common to all 17 isoforms of *sgg*. These insertions were created in an isogenic *w*^1118^ background (*w*^1118^{5905}); we used this background as a control [156]. Sleep phenotypes were measured as described above for 16 flies/sex/genotype in both the homozygous mutant lines and the trans-heterozygous lines, and the measures were repeated two times. Sleep phenotypes in each mutant were compared to the control using the ANOVA model *Y* = μ + *G* + *S* + *R* + *G×S* + *G*×*R* + *R*×*S* + *G*×*S*×*R* + *ε*, where *G* is genotype, *S* is sex, *R* is replicate, and *ε* is the error term. Males and females were analyzed separately using the reduced ANOVA model *Y* = μ + *G* + *R* + *G*×*R* + *ε*.

We also investigated potential interactions among these candidate genes by crossing them together. We crossed the three *Minos* lines in a full diallel design, so that all possible trans-heterozygous combinations were represented. Five virgin females of each line were crossed to five males of each line. *sgg* is located on the *X* chromosome; thus, male progeny from male *sgg* parents did not have the *sgg* mutation. We therefore restricted our analysis to males and females separately. We compared differences in sleep across trans-heterozygous genotypes using the ANOVA model *Y* = μ + *G* + *R* + *G*×*R* + *ε* as defined above. Genotypic effects can be decomposed into general combining ability (GCA), specific combining ability (SCA) and reciprocal effect (REC) using the full diallel, fixed effects design (Method 1, Model I) of Griffing [117]. The GCA of a mutation is the mean phenotype of the progeny bearing that mutation calculated as a deviation from the overall mean of all crosses; SCA is the deviation from the GCA and overall mean and reflects both dominance and epistatic interactions [67, 118]. Mean sleep phenotypes of all crosses in the full diallel design were designated as *Y*_*ij*_, where *Y* is the mean of the *ij*th genotype. We estimated the GCA of a mutation as (1/2n)(*X*_i._ + *X*_.j_)-(1/n^2^)*X*_.._, where *X*_*i*._ and *X*_.j_ are the sums of mean sleep phenotypes of heterozygotes with the *i*th and *j*th *Minos* element, respectively; *X*_.._ is the sum of mean sleep phenotypes for all genotypes; and n equals the number of mutations (3 in this case) [117]. We estimated SCA as (1/2)(*Y*_ij_ + *Y*_ji_)–(1/2n)(*X*_i._ + *X*_.i_ + *X*_j._ + *X*_.j_) + (1/n^2^)*X*_.._ [117]. REC effects were estimated using the formula (1/2)(*Y*_ij_−*Y*_ji_) [117]. The GCA is the average of a heterozygote relative to the other heterozygous effects, while SCA is a measure of dominance and/or epistatic effects [157, 158]. We partitioned the genetic variance for GCA, SCA, and REC components for each sex using the ANOVA model *Y* = μ + *GCA* + *SCA* + *REC* + *GCA*×*R* + SCA×*R* + REC×*R* +*ε*, where μ is the overall mean and *ε* is the error term [117]. We used DIALLEL-SAS to evaluate the ANOVA models and estimate GCA, SCA, and REC effects [159].

No *Mi{ET1}* lines were available for *mip120*, the candidate gene common to this study and the genome-wide association of sleep. However, many candidate genes were located along a 99.6-kb region on chromosome *2R*, including *crowded by cid*, *mip120* and *CG33156* from the present study, and *arrow*, *downstream of receptor kinase*, *mars*, *mip120*, *Vmat*, *CG13330*, and *CG13331* from the genome-wide association study [18]. We therefore tested three deficiency lines [160] *Df(2R)BSC273*, *Df(2R)BSC274*, and *Df(2R)BSC306*, which have breakpoints from 13159579–13502150, 13430464–13593272, and 13364133–13539889, respectively. These deletions were created in an isogenic *w*^1118^ background (*w*^1118^ {6326}); we used these backgrounds as a control [160]. The chromosomal deficiency stocks were maintained over a *CyO* balancer chromosome. We crossed 5 virgin females of each deficiency line to 5 males of the *w*^1118^ {6326} control and compared the heterozygotes bearing the deficiency to the control line. Sleep phenotypes were measured as described above for 16 flies/sex/genotype, and the measures were repeated two times. Sleep phenotypes were analyzed using the ANOVA model *Y* = μ + *G* + *S* + *G×S* + *R(G×S)* + *ε*, where *G* is deficiency genotype, *S* is sex, *R* is replicate, and *ε* is the error term. All stocks were obtained from the Bloomington, Indiana stock center. All analyses were implemented using SAS (Cary, NC).

**Data availability:** Raw DNA sequence data have been deposited in the NCBI SRA database under BioProject PRJNA369048, SRA database SRP098556.

## Supporting information

S1 Fig Sleep is homogeneous in the outbred population. The graphs show the differences among the sleep phenotypes in the control, long, and short sleep populations at Generation 0, prior to artificial selection. Mean ± SE are plotted for sexes combined. C1 and C2, control population replicates 1 and 2; L1 and L2, long-sleeping population replicates 1 and 2; S1 and S2, short-sleeping populations 1, and 2. (A), night sleep; (B), Day sleep; (C), night bout number; (D), day bout number; (E), night avg. bout length; (F), day avg. bout length; (G), sleep latency; (H), waking activity.
(PPTX)

S2 Fig Sleep traits not correlated with selection for long or short night sleep. (A), night bout number; (B), night bout number *CV*_E_; (C) day average bout length; (D) day average bout length *CV*_E_; (E), waking activity; and (F), waking activity *CV*_E_. (A, C, E), Mean ± SE are plotted for sexes combined. (B, D, F), mean is plotted for sexes combined. Light blue and dark blue triangles indicate Replicate 1 and Replicate 2 populations selected for long sleep; Light red and dark red squares indicate Replicate 1 and Replicate 2 populations selected for short sleep; and light gray and black circles indicate Replicate 1 and Replicate 2 control populations.
(PPTX)

S3 Fig Sleep architecture in replicate 2 populations. Shown are the percentages of flies sleeping, awake, or in a transient pause for each minute in a 24-hour day. (A), control females; (B), control males; (C), long sleeper females; (D), long sleeper males; (E), short sleeper females; (F), short sleeper males. Black/dark blue/dark red = sleep; gray = transient pause; light gray/light blue/light red = wake. White and black bars on the x-axis indicate the light and dark periods, respectively.
(PPTX)

S4 Fig Sleep in long- and short-sleeper populations not subjected to sleep deprivation. (A), the difference in 24-hour sleep from baseline (the average of days 1 and 2) sleep is plotted for day 3 (dark blue bars) and for day 4 (light blue bars). (B), the difference in day sleep from baseline for day 4.
(PPTX)

S5 Fig Minor allele frequency distributions in the outbred population prior to selection. (A), chromosome *2L*; (B), chromosome *2R*; (C), chromosome *3L*; (D) chromosome *3R*; (E) chromosome *X*; (F) all insertions and deletions.
(PPTX)

S6 Fig Effects of mutations on uncorrelated sleep traits. (A), day average bout length; (B), night bout number; (C), waking activity. *, *P* <0.05; ***, *P* <0.001; ****, *P* <0.0001.
(PPTX)

S7 Fig Sleep in *Minos* mutants not subjected to sleep deprivation. (A), the difference in 24-hour sleep from baseline (the average of days 1 and 2) sleep is plotted for day 3 (dark blue bars) and for day 4 (light blue bars). (B), the difference in day sleep from baseline for day 4. * *P* < 0.05; *P*-values reflect the comparison of deprived and/or recovery day sleep with baseline sleep.
(PPTX)

S8 Fig Estimated LD in candidate polymorphisms for chromosome *2R* across generations. For each population and generation, the LD is plotted as yellow for *r*^2^ values estimated to be below 0.8, and blue for *r*^2^ values ≥ 0.8.
(PPTX)

S1 Table Quantitative genetics of the response to selection for night sleep in both night sleep and associated sleep traits. For each sleep trait, the ANOVA results are presented. gen, generation; rep, replicate; sel, long or short selection scheme; *d*.*f*., degrees of freedom; M.S., Type III mean squares; *F*, *F* ratio statistic; *P*, *P*-value.
(XLSX)

S2 Table Quantitative genetics of the response to selection for long or short night sleep per generation in both night sleep and associated sleep traits. For each sleep trait listed, the ANOVA results are presented. rep, replicate; sel, long or short selection scheme; *d*.*f*., degrees of freedom; M.S., Type III mean squares; *F*, *F* ratio statistic; *P*, *P*-value.
(XLSX)

S3 Table Quantitative genetics of sleep in control populations. For each sleep trait listed, the ANOVA results are presented. rep, replicate; *d*.*f*., degrees of freedom; M. S., Type III mean squares; *F*, *F* ratio statistic; *P*, *P*-value.
(XLSX)

S4 Table Response of sleep trait coefficient of environmental variance (*CV*_E_) to selection for long or short night sleep duration. For each sleep *CV*_E_ trait listed, the ANOVA results are presented. *d*.*f*., degrees of freedom; M.S., Type III mean squares; *F*, *F* ratio statistic; *P*, *P*-value.
(XLSX)

S5 Table Correlated response of lifespan and egg-to-adult viability to selection for long or short night sleep. rep, replicate; sel, long, short, or control selection scheme; *d*.*f*., degrees of freedom; MS, Type III mean squares; *F*, *F* ratio statistic; *P*, *P*-value.
(XLSX)

S6 Table Polymorphisms significant for the logistic regression. Listed are the chromosome, position in base pairs, the selection population (L1 = long replicate 1; S1 = short replicate 1; L2 = long replicate 2; S2 = short replicate 2), selection scheme (long or short), sex, replicate, minor allele frequency for each generation, which generation(s) the polymorphism was significant for the CMH test, whether the polymorphism is located in an inversion region, and the *P*-values for the generation, selection scheme (sel), generation×sel, and the lack-of-fit statistic.
(XLSX)

S7 Table Candidate genes for night sleep duration. Each polymorphism is listed by chromosome, position in base pairs, and type of polymorphism (SNP or indel). Variants whose average allele frequency difference between the long- and short-sleepers exceeded the largest change expected due to drift are indicated, as are the putative LD blocks for long-and short-sleeper populations. If the polymorphism was within a gene region or ± 1kb from a gene region, the Flybase ID, gene symbol, and polymorphism location are given. The midpoint recombination rates (RRC) as calculated by Fiston-Lavier et al.^g^ and as mapped by Comeron et al.^h^ are given. Genes previously identified as candidate genes for night sleep or night sleep CVE from the genome-wide association study of the DGRP are indicated. Genes previously implicated in other sleep or circadian rhythm studies are indicated. Polymorphisms falling within two or more genes are included with a separate row for each gene.
(XLSX)

S8 Table Overlap among artificial selection candidate genes and genes from the genome-wide association study of sleep. Listed are the gene symbol and the number of polymorphisms per gene (if any were found) for each trait that were identified in the GWAS.
(XLSX)

S9 Table Known physical and genetic interactions with artificial selection candidate genes. Candidate genes with known physical and genetic (enhanceable or suppressible) interactions are indicated. In some cases, the candidate sleep gene is the source; in others, it is the target. This is indicated by the Source gene and Target gene columns. Also listed are Flybase ID numbers, whether the genes were candidate genes from a genome-wide association study, previously identified sleep genes, or previously identified circadian genes. Genes without highlights have average allele frequency differences greater than that predicted by drift simulation; highlighted genes did not pass this threshold difference. Publications supporting the known physical and genetic interactions among genes are given as PubMed ID’s and are listed in S10 Table.
(XLSX)

S10 Table List of publications supporting physical and genetic interactions. Publications are numbered according to the gene entries in S9 Table.
(DOCX)

S11 Table Results of tests of homozygous *Minos* element insertions in candidate genes. For each sleep trait, the ANOVA results are presented. *P* values reflect the comparison of the *Minos* element in the gene listed versus the isogenic control. rep, replicate; *d*.*f*., degrees of freedom; M.S., Type III mean squares; *F*, *F* ratio statistic; *P*, *P*-value. Significant comparisons are shown in bold.
(XLSX)

S12 Table Results of tests of trans-heterozygous *Minos* element insertions in candidate genes. For each sleep trait, the ANOVA results are presented. *P* values reflect the comparison among all trans-heterozygous crosses. rep, replicate; *d*.*f*., degrees of freedom; M.S., Type III mean squares; *F*, *F* ratio statistic; *P*, *P*-value. Significant comparisons are shown in bold.
(XLSX)

S13 Table Results of tests of general combining ability of trans-heterozygous Minos element insertions in candidate genes. For each sleep trait, the ANOVA results are presented. *P* values reflect the comparison among all trans-heterozygous crosses. rep, replicate; *d*.*f*., degrees of freedom; M.S., Type III mean squares; *F*, *F* ratio statistic; *P*, *P*-value. Significant comparisons are shown in bold.
(XLSX)

S14 Table General combining effects for each *Minos* mutation. For each sleep trait, the estimate of general combining ability (GCA) is listed, along with the standard error. The *t*-test statistic and corresponding *P* value indicate the degree of significance of the GCA effect. Significant comparisons are shown in bold.
(XLSX)

S15 Table Specific combining effects for each *Minos* mutation. For each sleep trait, the estimate of specific combining ability (SCA) is listed, along with the standard error. The *t*-test statistic and corresponding *P* value indicate the degree of significance of the SCA effect. Significant comparisons are shown in bold.
(XLSX)

S16 Table Reciprocal combining effects for each *Minos* mutation. For each sleep trait, the estimated reciprocal effect (REC) is listed, along with the standard error. The *t*-test statistic and corresponding *P* value indicate the degree of significance of the REC effect. Significant comparisons are shown in bold.
(XLSX)

S17 Table Mean phenotype for each cross. For each sleep trait, the genotype of the male, female, and progeny are listed, along with the number of flies tested, the mean phenotype, and the standard error.
(XLSX)

S18 Table Results of deficiency mapping. For each sleep trait, the ANOVA results are presented. *P* values reflect the comparison of the deficiency listed versus the isogenic control. rep, replicate; *d*.*f*., degrees of freedom; M.S., Type III mean squares; *F*, *F* ratio statistic; *P*, *P*-value. Significant comparisons are shown in bold.
(XLSX)
