# Supplementary material for: Selection for long and short sleep duration in Drosophila melanogaster reveals the complex genetic network underlying natural variation in sleep
Source: PLoS Genet. 2017 Dec 14;13(12):e1007098. doi: 10.1371/journal.pgen.1007098 (PMC5730107; doi:10.1371/journal.pgen.1007098)
Supplement: S1 Fig — The graphs show the differences among the sleep phenotypes in the control, long, and short sleep populations at Generation 0, prior to artificial selection. Mean ± SE are plotted for sexes combined. C1 and C2, control population replicates 1 and 2; L1 and L2, long-sleeping population replicates 1 and 2; S1 and S2, short-sleeping populations 1, and 2. (A), night sleep; (B), Day sleep; (C), night bout number; (D), day bout number; (E), night avg. bout length; (F), day avg. bout length; (G), sleep latency; (H), waking activity. (PPTX) [file pgen.1007098.s001.pptx]

## Slide 1
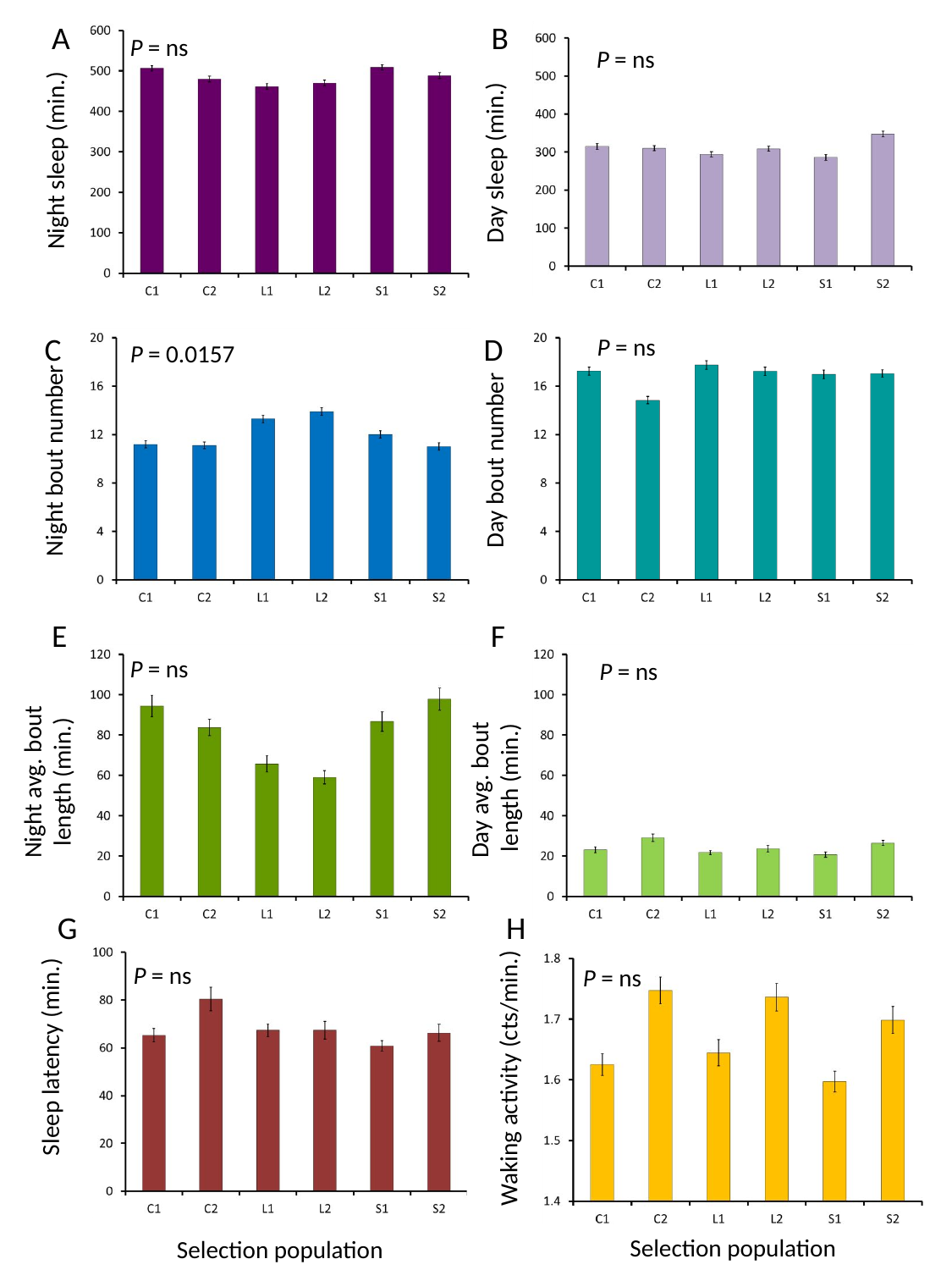

A
B
P = ns
P = ns
P = ns
P = ns
Night sleep (min.)
Day sleep (min.)
C
D
P = 0.0157
Night bout number
Day bout number
E
F
P = ns
Night avg. bout
length (min.)
Day avg. bout
length (min.)
G
H
Waking activity (cts/min.)
P = ns
Sleep latency (min.)
P = ns
Selection population
Selection population
