# Supplementary material for: Selection for long and short sleep duration in Drosophila melanogaster reveals the complex genetic network underlying natural variation in sleep
Source: PLoS Genet. 2017 Dec 14;13(12):e1007098. doi: 10.1371/journal.pgen.1007098 (PMC5730107; doi:10.1371/journal.pgen.1007098)
Supplement: S2 Fig — (A), night bout number; (B), night bout number CVE; (C) day average bout length; (D) day average bout length CVE; (E), waking activity; and (F), waking activity CVE. (A, C, E), Mean ± SE are plotted for sexes combined. (B, D, F), mean is plotted for sexes combined. Light blue and dark blue triangles indicate Replicate 1 and Replicate 2 populations selected for long sleep; Light red and dark red squares indicate Replicate 1 and Replicate 2 populations selected for short sleep; and light gray and black circles indicate Replicate 1 and Replicate 2 control populations. (PPTX) [file pgen.1007098.s002.pptx]

## Slide 1
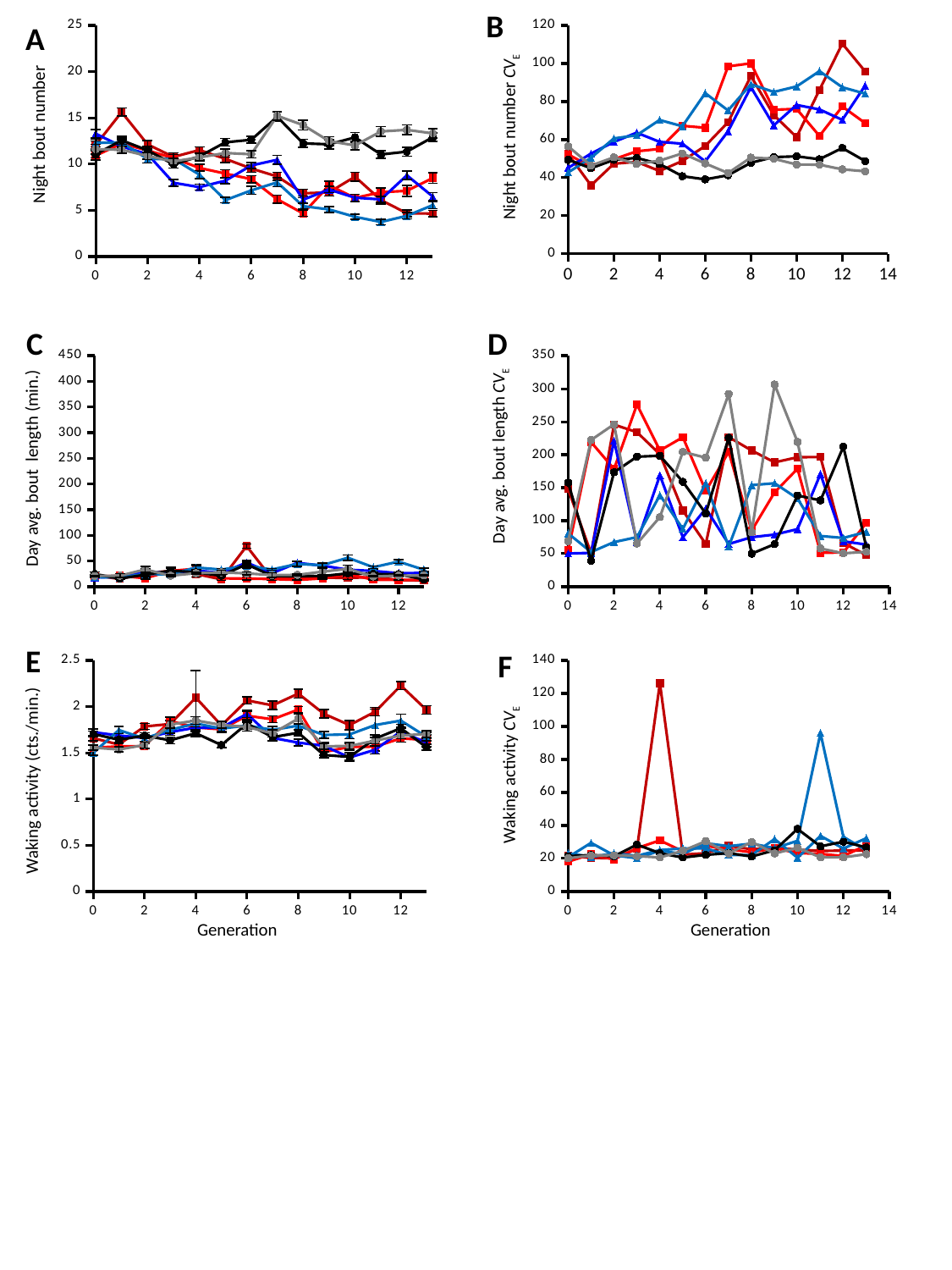

B
A
### Chart
| Category | C1 | C2 | L1 | L2 | S1 | S2 |
|---|---|---|---|---|---|---|
### Chart
| Category | boutn C1 1 | boutn C2 2 | boutn L1 1 | boutn L2 2 | boutn S1 1 | boutn S2 2 |
|---|---|---|---|---|---|---|Night bout number CVE
Night bout number
C
D
### Chart
| Category | C1 | C2 | L1 | L2 | S1 | S2 |
|---|---|---|---|---|---|---|
### Chart
| Category | avgboutd C1 1 | avgboutd C2 2 | avgboutd L1 1 | avgboutd L2 2 | avgboutd S1 1 | avgboutd S2 2 |
|---|---|---|---|---|---|---|Day avg. bout length CVE
Day avg. bout length (min.)
E
F
### Chart
| Category | C1 | C2 | L1 | L2 | S1 | S2 |
|---|---|---|---|---|---|---|
### Chart
| Category | wakeact C1 1 | wakeact C2 2 | wakeact L1 1 | wakeact L2 2 | wakeact S1 1 | wakeact S2 2 |
|---|---|---|---|---|---|---|Waking activity (cts./min.)
Waking activity CVE
Generation
Generation
