# Supplementary material for: Selection for long and short sleep duration in Drosophila melanogaster reveals the complex genetic network underlying natural variation in sleep
Source: PLoS Genet. 2017 Dec 14;13(12):e1007098. doi: 10.1371/journal.pgen.1007098 (PMC5730107; doi:10.1371/journal.pgen.1007098)
Supplement: S4 Fig — (A), the difference in 24-hour sleep from baseline (the average of days 1 and 2) sleep is plotted for day 3 (dark blue bars) and for day 4 (light blue bars). (B), the difference in day sleep from baseline for day 4. (PPTX) [file pgen.1007098.s004.pptx]

## Slide 1
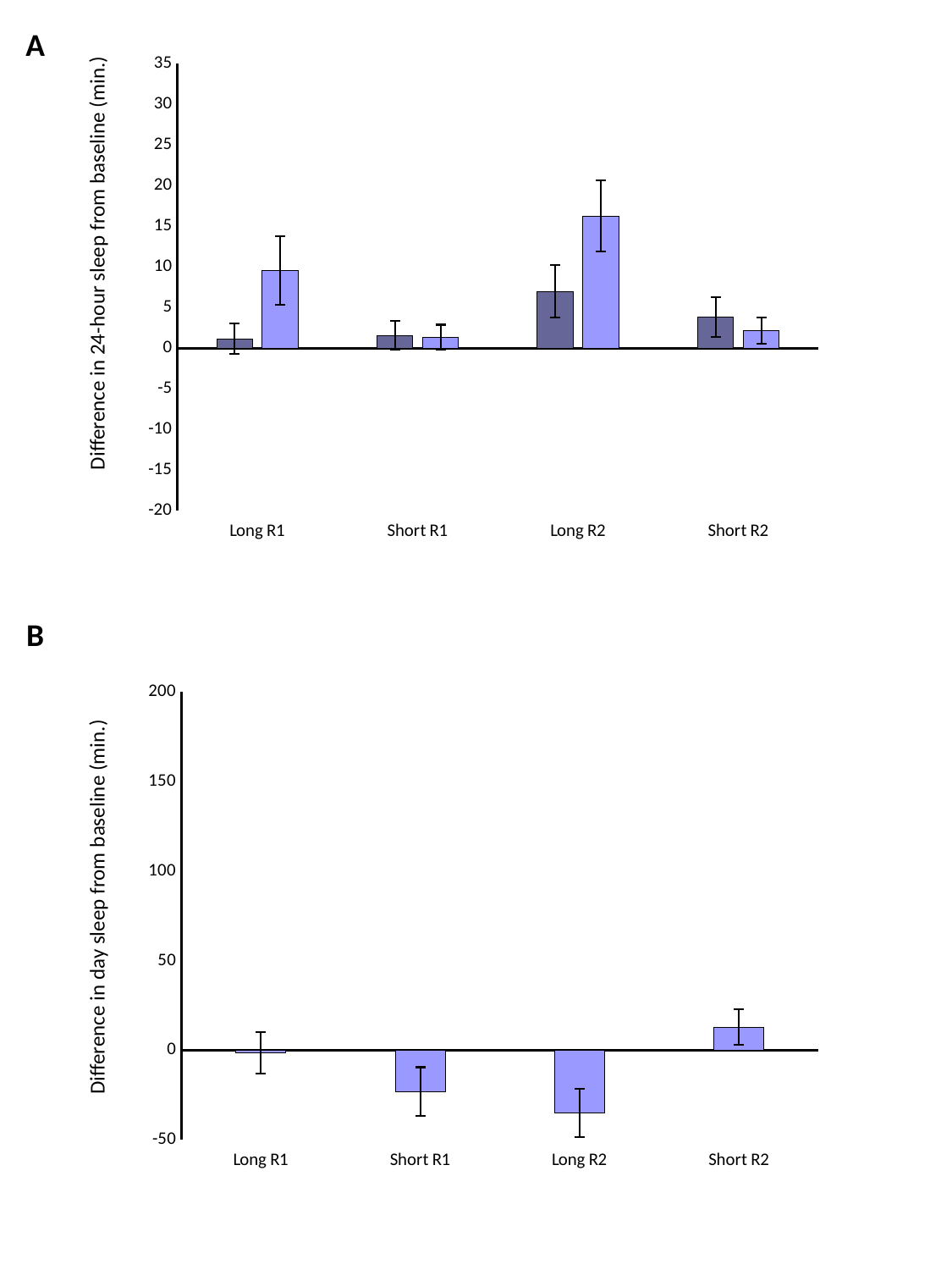

A
### Chart
| Category | Mean(avgbout24_dep_diff) | Mean(avgbout24_reb_diff) |
|---|---|---|
| Long R1 | 1.1233980301 | 9.5620676501 |
| Short R1 | 1.5622368946 | 1.3507618775 |
| Long R2 | 6.9716109227 | 16.22172922 |
| Short R2 | 3.8125629987 | 2.1096763705 |Difference in 24-hour sleep from baseline (min.)
B
### Chart
| Category | Mean(sleepd_reb_dep) |
|---|---|
| Long R1 | -1.475409836 |
| Short R1 | -23.10344828 |
| Long R2 | -34.99152542 |
| Short R2 | 12.796610169 |Difference in day sleep from baseline (min.)
