# Supplementary material for: Selection for long and short sleep duration in Drosophila melanogaster reveals the complex genetic network underlying natural variation in sleep
Source: PLoS Genet. 2017 Dec 14;13(12):e1007098. doi: 10.1371/journal.pgen.1007098 (PMC5730107; doi:10.1371/journal.pgen.1007098)
Supplement: S5 Fig — (A), chromosome 2L; (B), chromosome 2R; (C), chromosome 3L; (D) chromosome 3R; (E) chromosome X; (F) all insertions and deletions. (PPTX) [file pgen.1007098.s005.pptx]

## Slide 1
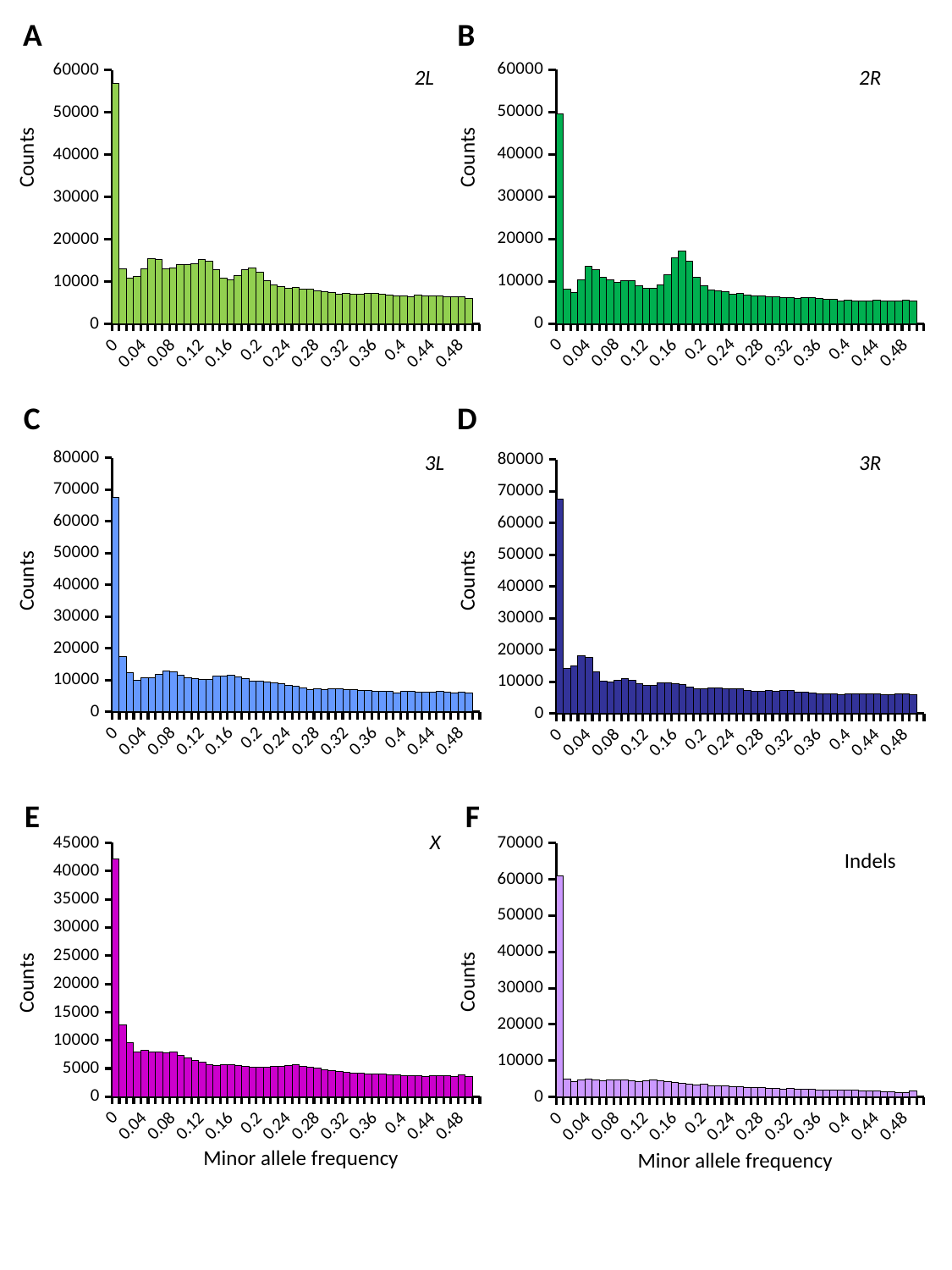

A
B
### Chart
| Category | Chrs 2R Count |
|---|---|
| 0 | 49491.0 |
| 0.01 | 8106.0 |
| 0.02 | 7339.0 |
| 0.03 | 10401.0 |
| 0.04 | 13433.0 |
| 0.05 | 12740.0 |
| 0.06 | 10993.0 |
| 0.07 | 10298.0 |
| 0.08 | 9814.0 |
| 0.09 | 10112.0 |
| 0.1 | 10213.0 |
| 0.11 | 8835.0 |
| 0.12 | 8350.0 |
| 0.13 | 8285.0 |
| 0.14 | 9120.0 |
| 0.15 | 11590.0 |
| 0.16 | 15518.0 |
| 0.17 | 17209.0 |
| 0.18 | 14780.0 |
| 0.19 | 11003.0 |
| 0.2 | 8912.0 |
| 0.21 | 8015.0 |
| 0.22 | 7763.0 |
| 0.23 | 7544.0 |
| 0.24 | 7017.0 |
| 0.25 | 7062.0 |
| 0.26 | 6717.0 |
| 0.27 | 6617.0 |
| 0.28 | 6514.0 |
| 0.29 | 6376.0 |
| 0.3 | 6281.0 |
| 0.31 | 6176.0 |
| 0.32 | 6147.0 |
| 0.33 | 5872.0 |
| 0.34 | 6057.0 |
| 0.35 | 6048.0 |
| 0.36 | 5898.0 |
| 0.37 | 5663.0 |
| 0.38 | 5650.0 |
| 0.39 | 5375.0 |
| 0.4 | 5589.0 |
| 0.41 | 5321.0 |
| 0.42 | 5408.0 |
| 0.43 | 5395.0 |
| 0.44 | 5465.0 |
| 0.45 | 5357.0 |
| 0.46 | 5236.0 |
| 0.47 | 5310.0 |
| 0.48 | 5484.0 |
| 0.49 | 5342.0 |
| 0.5 | 135.0 |
### Chart
| Category | Chrs 2L Count |
|---|---|
| 0 | 56876.0 |
| 0.01 | 13062.0 |
| 0.02 | 10901.0 |
| 0.03 | 11213.0 |
| 0.04 | 13021.0 |
| 0.05 | 15408.0 |
| 0.06 | 15189.0 |
| 0.07 | 13064.0 |
| 0.08 | 13117.0 |
| 0.09 | 13963.0 |
| 0.1 | 14069.0 |
| 0.11 | 14220.0 |
| 0.12 | 15180.0 |
| 0.13 | 14897.0 |
| 0.14 | 12842.0 |
| 0.15 | 10882.0 |
| 0.16 | 10491.0 |
| 0.17 | 11491.0 |
| 0.18 | 12730.0 |
| 0.19 | 13151.0 |
| 0.2 | 12187.0 |
| 0.21 | 10177.0 |
| 0.22 | 9110.0 |
| 0.23 | 8716.0 |
| 0.24 | 8440.0 |
| 0.25 | 8573.0 |
| 0.26 | 8197.0 |
| 0.27 | 8233.0 |
| 0.28 | 7862.0 |
| 0.29 | 7590.0 |
| 0.3 | 7340.0 |
| 0.31 | 7107.0 |
| 0.32 | 7122.0 |
| 0.33 | 7038.0 |
| 0.34 | 6994.0 |
| 0.35 | 7234.0 |
| 0.36 | 7246.0 |
| 0.37 | 7050.0 |
| 0.38 | 6905.0 |
| 0.39 | 6540.0 |
| 0.4 | 6547.0 |
| 0.41 | 6483.0 |
| 0.42 | 6737.0 |
| 0.43 | 6663.0 |
| 0.44 | 6701.0 |
| 0.45 | 6598.0 |
| 0.46 | 6469.0 |
| 0.47 | 6455.0 |
| 0.48 | 6405.0 |
| 0.49 | 6073.0 |
| 0.5 | 175.0 |2R
2L
Counts
Counts
C
D
### Chart
| Category | Chrs 3L Count |
|---|---|
| 0 | 67674.0 |
| 0.01 | 17287.0 |
| 0.02 | 12377.0 |
| 0.03 | 9906.0 |
| 0.04 | 10682.0 |
| 0.05 | 10741.0 |
| 0.06 | 11773.0 |
| 0.07 | 12988.0 |
| 0.08 | 12609.0 |
| 0.09 | 11605.0 |
| 0.1 | 10847.0 |
| 0.11 | 10367.0 |
| 0.12 | 10125.0 |
| 0.13 | 10191.0 |
| 0.14 | 11233.0 |
| 0.15 | 11350.0 |
| 0.16 | 11609.0 |
| 0.17 | 11090.0 |
| 0.18 | 10559.0 |
| 0.19 | 9658.0 |
| 0.2 | 9712.0 |
| 0.21 | 9381.0 |
| 0.22 | 9071.0 |
| 0.23 | 8837.0 |
| 0.24 | 8290.0 |
| 0.25 | 7978.0 |
| 0.26 | 7635.0 |
| 0.27 | 7109.0 |
| 0.28 | 7233.0 |
| 0.29 | 6978.0 |
| 0.3 | 7228.0 |
| 0.31 | 7192.0 |
| 0.32 | 6961.0 |
| 0.33 | 7005.0 |
| 0.34 | 6780.0 |
| 0.35 | 6669.0 |
| 0.36 | 6596.0 |
| 0.37 | 6370.0 |
| 0.38 | 6337.0 |
| 0.39 | 6059.0 |
| 0.4 | 6405.0 |
| 0.41 | 6339.0 |
| 0.42 | 6275.0 |
| 0.43 | 6235.0 |
| 0.44 | 6326.0 |
| 0.45 | 6334.0 |
| 0.46 | 6259.0 |
| 0.47 | 6064.0 |
| 0.48 | 6214.0 |
| 0.49 | 5983.0 |
| 0.5 | 157.0 |3L
3R
### Chart
| Category | Chrs 3R Count |
|---|---|
| 0 | 67498.0 |
| 0.01 | 14272.0 |
| 0.02 | 14915.0 |
| 0.03 | 18071.0 |
| 0.04 | 17541.0 |
| 0.05 | 13109.0 |
| 0.06 | 10279.0 |
| 0.07 | 9899.0 |
| 0.08 | 10443.0 |
| 0.09 | 11006.0 |
| 0.1 | 10339.0 |
| 0.11 | 9357.0 |
| 0.12 | 8888.0 |
| 0.13 | 8950.0 |
| 0.14 | 9506.0 |
| 0.15 | 9528.0 |
| 0.16 | 9329.0 |
| 0.17 | 9004.0 |
| 0.18 | 8195.0 |
| 0.19 | 7898.0 |
| 0.2 | 7896.0 |
| 0.21 | 7990.0 |
| 0.22 | 8055.0 |
| 0.23 | 7834.0 |
| 0.24 | 7713.0 |
| 0.25 | 7704.0 |
| 0.26 | 7243.0 |
| 0.27 | 6896.0 |
| 0.28 | 6939.0 |
| 0.29 | 7127.0 |
| 0.3 | 7100.0 |
| 0.31 | 7105.0 |
| 0.32 | 7135.0 |
| 0.33 | 6652.0 |
| 0.34 | 6607.0 |
| 0.35 | 6347.0 |
| 0.36 | 6084.0 |
| 0.37 | 6214.0 |
| 0.38 | 6228.0 |
| 0.39 | 6024.0 |
| 0.4 | 6244.0 |
| 0.41 | 6112.0 |
| 0.42 | 6196.0 |
| 0.43 | 6116.0 |
| 0.44 | 6129.0 |
| 0.45 | 5958.0 |
| 0.46 | 5937.0 |
| 0.47 | 6117.0 |
| 0.48 | 6292.0 |
| 0.49 | 5997.0 |
| 0.5 | 188.0 |Counts
Counts
F
E
X
### Chart
| Category | Chrs X Count |
|---|---|
| 0 | 42105.0 |
| 0.01 | 12796.0 |
| 0.02 | 9626.0 |
| 0.03 | 7918.0 |
| 0.04 | 8152.0 |
| 0.05 | 7870.0 |
| 0.06 | 7897.0 |
| 0.07 | 7746.0 |
| 0.08 | 7905.0 |
| 0.09 | 7382.0 |
| 0.1 | 6879.0 |
| 0.11 | 6485.0 |
| 0.12 | 6091.0 |
| 0.13 | 5716.0 |
| 0.14 | 5544.0 |
| 0.15 | 5626.0 |
| 0.16 | 5666.0 |
| 0.17 | 5533.0 |
| 0.18 | 5415.0 |
| 0.19 | 5274.0 |
| 0.2 | 5258.0 |
| 0.21 | 5307.0 |
| 0.22 | 5334.0 |
| 0.23 | 5333.0 |
| 0.24 | 5495.0 |
| 0.25 | 5734.0 |
| 0.26 | 5429.0 |
| 0.27 | 5177.0 |
| 0.28 | 5041.0 |
| 0.29 | 4799.0 |
| 0.3 | 4612.0 |
| 0.31 | 4479.0 |
| 0.32 | 4276.0 |
| 0.33 | 4177.0 |
| 0.34 | 4107.0 |
| 0.35 | 4100.0 |
| 0.36 | 4084.0 |
| 0.37 | 4017.0 |
| 0.38 | 3844.0 |
| 0.39 | 3828.0 |
| 0.4 | 3683.0 |
| 0.41 | 3706.0 |
| 0.42 | 3737.0 |
| 0.43 | 3649.0 |
| 0.44 | 3672.0 |
| 0.45 | 3769.0 |
| 0.46 | 3691.0 |
| 0.47 | 3604.0 |
| 0.48 | 3824.0 |
| 0.49 | 3551.0 |
| 0.5 | 148.0 |
### Chart
| Category | Indel Count |
|---|---|
| 0 | 61059.0 |
| 0.01 | 4947.0 |
| 0.02 | 4227.0 |
| 0.03 | 4788.0 |
| 0.04 | 4889.0 |
| 0.05 | 4769.0 |
| 0.06 | 4501.0 |
| 0.07 | 4688.0 |
| 0.08 | 4647.0 |
| 0.09 | 4615.0 |
| 0.1 | 4364.0 |
| 0.11 | 4298.0 |
| 0.12 | 4533.0 |
| 0.13 | 4738.0 |
| 0.14 | 4430.0 |
| 0.15 | 4176.0 |
| 0.16 | 3955.0 |
| 0.17 | 3774.0 |
| 0.18 | 3633.0 |
| 0.19 | 3356.0 |
| 0.2 | 3438.0 |
| 0.21 | 3122.0 |
| 0.22 | 3111.0 |
| 0.23 | 3001.0 |
| 0.24 | 2845.0 |
| 0.25 | 2772.0 |
| 0.26 | 2599.0 |
| 0.27 | 2682.0 |
| 0.28 | 2501.0 |
| 0.29 | 2394.0 |
| 0.3 | 2384.0 |
| 0.31 | 2166.0 |
| 0.32 | 2293.0 |
| 0.33 | 2196.0 |
| 0.34 | 2167.0 |
| 0.35 | 2074.0 |
| 0.36 | 1964.0 |
| 0.37 | 1953.0 |
| 0.38 | 1857.0 |
| 0.39 | 1795.0 |
| 0.4 | 1832.0 |
| 0.41 | 1782.0 |
| 0.42 | 1724.0 |
| 0.43 | 1670.0 |
| 0.44 | 1615.0 |
| 0.45 | 1459.0 |
| 0.46 | 1343.0 |
| 0.47 | 1268.0 |
| 0.48 | 1202.0 |
| 0.49 | 1777.0 |
| 0.5 | 133.0 |Indels
Counts
Counts
Minor allele frequency
Minor allele frequency
