# Supplementary material for: Selection for long and short sleep duration in Drosophila melanogaster reveals the complex genetic network underlying natural variation in sleep
Source: PLoS Genet. 2017 Dec 14;13(12):e1007098. doi: 10.1371/journal.pgen.1007098 (PMC5730107; doi:10.1371/journal.pgen.1007098)
Supplement: S6 Fig — (A), day average bout length; (B), night bout number; (C), waking activity. *, P <0.05; ***, P <0.001; ****, P <0.0001. (PPTX) [file pgen.1007098.s006.pptx]

## Slide 1
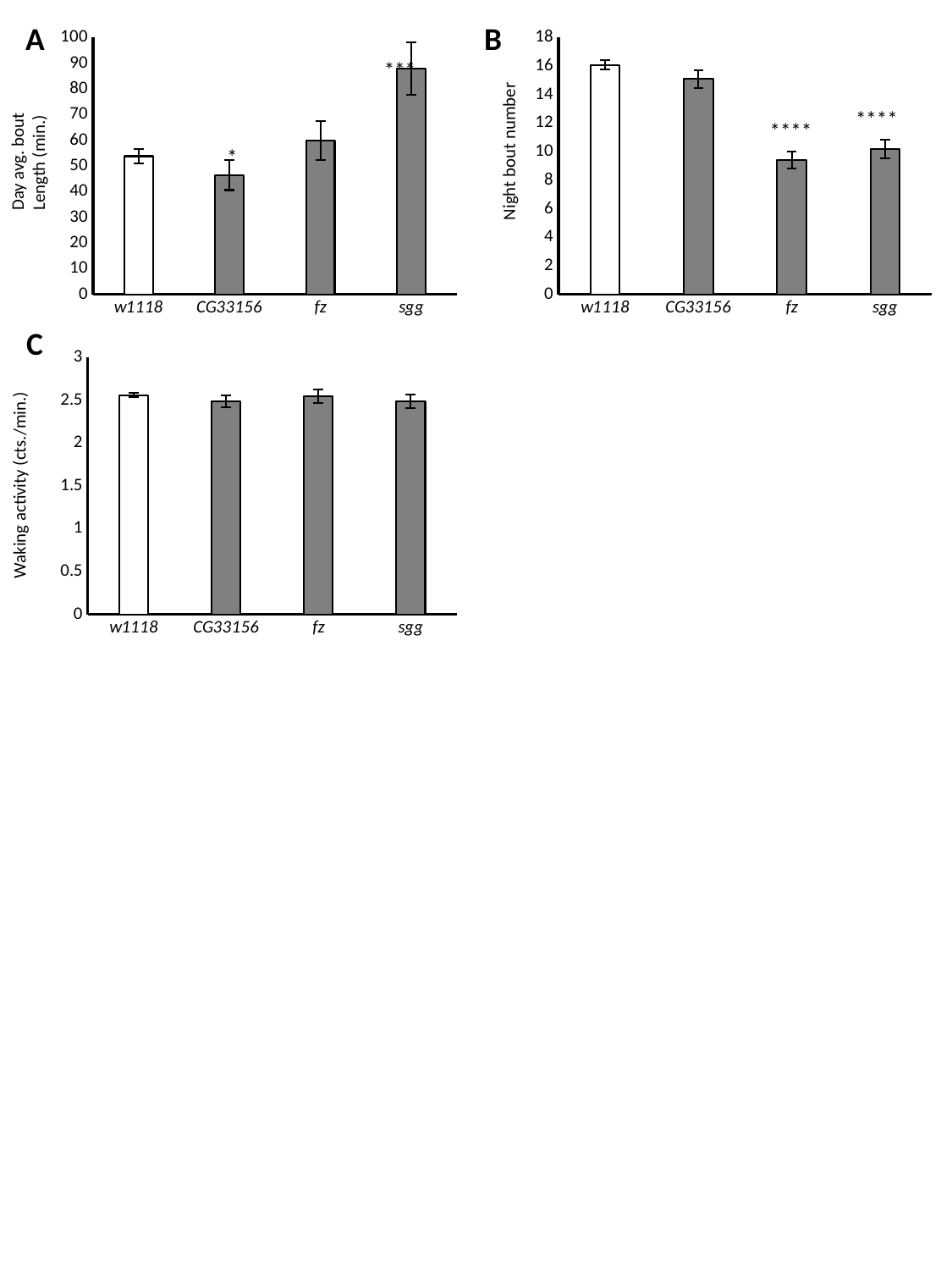

A
B
### Chart
| Category | |
|---|---|
| w1118 | 53.763235585 |
| CG33156 | 46.422335059 |
| fz | 59.892580947 |
| sgg | 87.975203114 |
### Chart
| Category | |
|---|---|
| w1118 | 16.080225989 |
| CG33156 | 15.094927536 |
| fz | 9.3885245902 |
| sgg | 10.173188406 |***
Night bout number
****
Day avg. bout
Length (min.)
****
*
C
### Chart
| Category | |
|---|---|
| w1118 | 2.5587091808 |
| CG33156 | 2.4877027174 |
| fz | 2.5470214262 |
| sgg | 2.4905291957 |Waking activity (cts./min.)
