# Supplementary material for: Selection for long and short sleep duration in Drosophila melanogaster reveals the complex genetic network underlying natural variation in sleep
Source: PLoS Genet. 2017 Dec 14;13(12):e1007098. doi: 10.1371/journal.pgen.1007098 (PMC5730107; doi:10.1371/journal.pgen.1007098)
Supplement: S7 Fig — (A), the difference in 24-hour sleep from baseline (the average of days 1 and 2) sleep is plotted for day 3 (dark blue bars) and for day 4 (light blue bars). (B), the difference in day sleep from baseline for day 4. * P < 0.05; P-values reflect the comparison of deprived and/or recovery day sleep with baseline sleep. (PPTX) [file pgen.1007098.s007.pptx]

## Slide 1
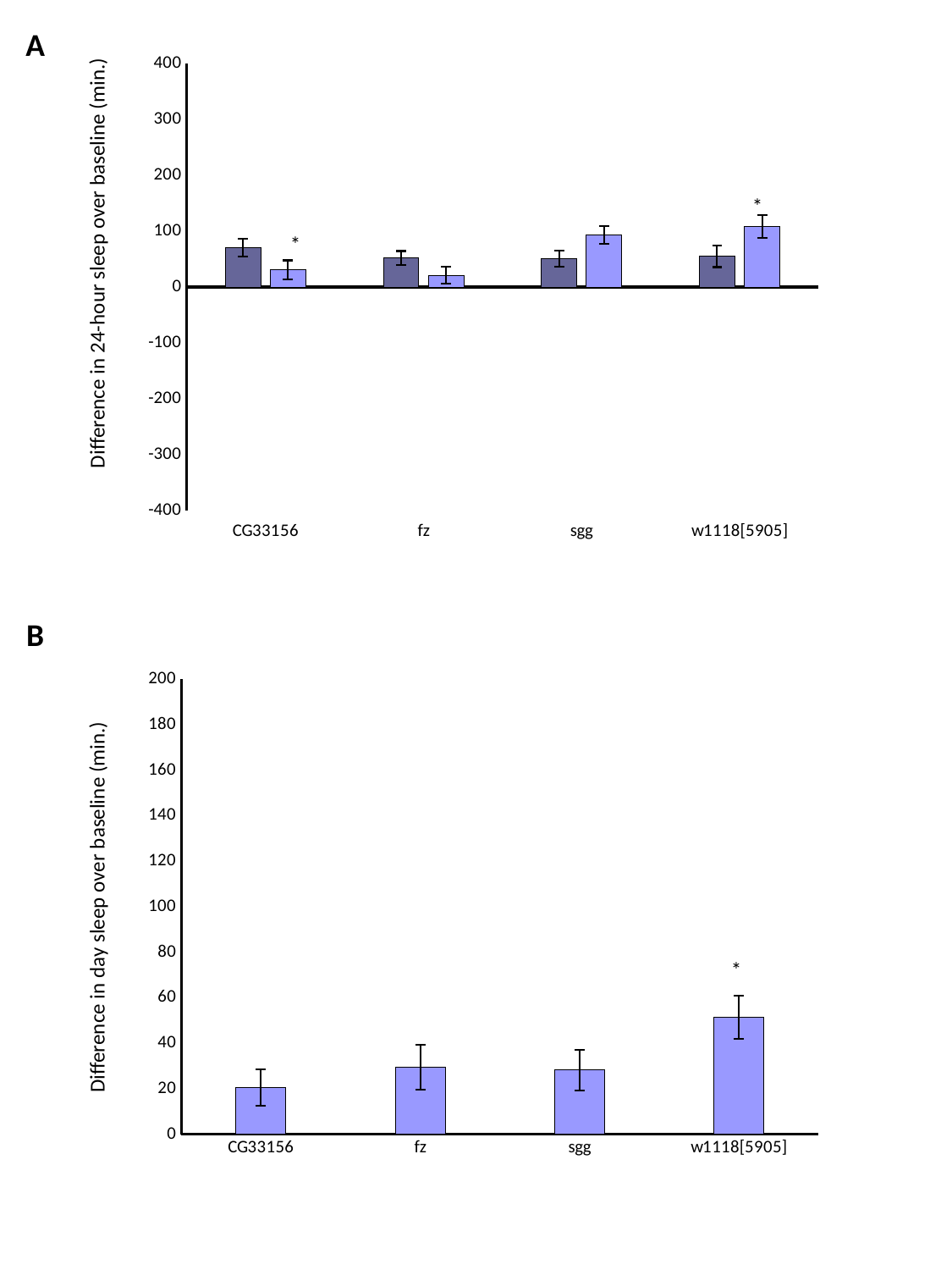

A
### Chart
| Category | Mean(sleep24_diff_dep) | Mean(sleep24_diff_reb) |
|---|---|---|
| CG33156 | 70.095238095 | 30.634920635 |
| fz | 51.76984127 | 20.658730159 |
| sgg | 50.80952381 | 92.857142857 |
| w1118[5905] | 54.6 | 107.85 |*
*
Difference in 24-hour sleep over baseline (min.)
B
### Chart
| Category | Mean(sleepd_diff_reb) |
|---|---|
| CG33156 | 20.611111111 |
| fz | 29.444444444 |
| sgg | 28.182539683 |
| w1118[5905] | 51.4 |Difference in day sleep over baseline (min.)
*
