# Supplementary material for: Selection for long and short sleep duration in Drosophila melanogaster reveals the complex genetic network underlying natural variation in sleep
Source: PLoS Genet. 2017 Dec 14;13(12):e1007098. doi: 10.1371/journal.pgen.1007098 (PMC5730107; doi:10.1371/journal.pgen.1007098)
Supplement: S8 Fig — For each population and generation, the LD is plotted as yellow for r2 values estimated to be below 0.8, and blue for r2 values ≥ 0.8. (PPTX) [file pgen.1007098.s008.pptx]

## Slide 1
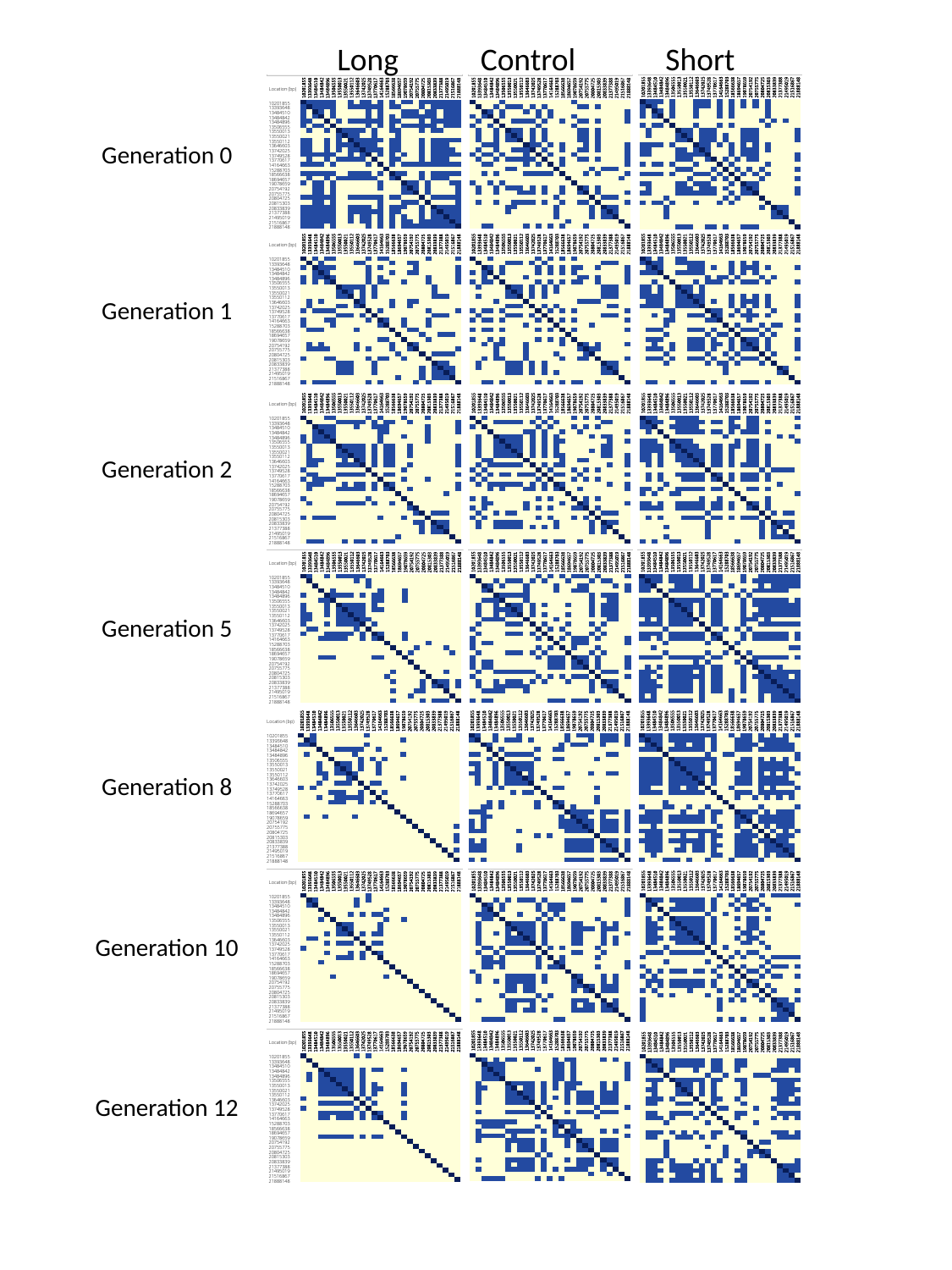

Long
Short
Control
Generation 0
Generation 1
Generation 2
Generation 5
Generation 8
Generation 10
Generation 12
