# Supplementary material for: Selection for long and short sleep duration in Drosophila melanogaster reveals the complex genetic network underlying natural variation in sleep
Source: PLoS Genet. 2017 Dec 14;13(12):e1007098. doi: 10.1371/journal.pgen.1007098 (PMC5730107; doi:10.1371/journal.pgen.1007098)
Supplement: S10 Table — Publications are numbered according to the gene entries in S9 Table. (DOCX) [file pgen.1007098.s018.docx]

**Table S10. List of publications supporting physical and genetic interactions.** Publications are numbered according to the gene entries in Table S9.

1: Cervantes-Sandoval I, Chakraborty M, MacMullen C, Davis RL. Scribble Scaffolds

a Signalosome for Active Forgetting. Neuron. 2016 Jun 15;90(6):1230-42. doi:

10.1016/j.neuron.2016.05.010. PubMed PMID: 27263975; PubMed Central PMCID:

PMC4926877.

2: Peng Q, Wang Y, Li M, Yuan D, Xu M, Li C, Gong Z, Jiao R, Liu L.

cGMP-Dependent Protein Kinase Encoded by foraging Regulates Motor Axon Guidance

in Drosophila by Suppressing Lola Function. J Neurosci. 2016 Apr

20;36(16):4635-46. doi: 10.1523/JNEUROSCI.3726-15.2016. PubMed PMID: 27098704.

3: Hwangbo DS, Biteau B, Rath S, Kim J, Jasper H. Control of apoptosis by

Drosophila DCAF12. Dev Biol. 2016 May 1;413(1):50-9. doi:

10.1016/j.ydbio.2016.03.003. PubMed PMID: 26972874; PubMed Central PMCID:

PMC5106244.

4: Orme MH, Liccardi G, Moderau N, Feltham R, Wicky-John S, Tenev T, Aram L,

Wilson R, Bianchi K, Morris O, Monteiro Domingues C, Robertson D, Tare M, Wepf A,

Williams D, Bergmann A, Gstaiger M, Arama E, Ribeiro PS, Meier P. The

unconventional myosin CRINKLED and its mammalian orthologue MYO7A regulate

caspases in their signalling roles. Nat Commun. 2016 Mar 10;7:10972. doi:

10.1038/ncomms10972. PubMed PMID: 26960254; PubMed Central PMCID: PMC4792956.

5: Sivachenko A, Gordon HB, Kimball SS, Gavin EJ, Bonkowsky JL, Letsou A.

Neurodegeneration in a Drosophila model of adrenoleukodystrophy: the roles of the

Bubblegum and Double bubble acyl-CoA synthetases. Dis Model Mech. 2016

Apr;9(4):377-87. doi: 10.1242/dmm.022244. PubMed PMID: 26893370; PubMed Central

PMCID: PMC4852500.

6: Keder A, Rives-Quinto N, Aerne BL, Franco M, Tapon N, Carmena A. The hippo

pathway core cassette regulates asymmetric cell division. Curr Biol. 2015 Nov

2;25(21):2739-50. doi: 10.1016/j.cub.2015.08.064. PubMed PMID: 26592338.

7: Moncrieff S, Moncan M, Scialpi F, Ditzel M. Regulation of hedgehog Ligand

Expression by the N-End Rule Ubiquitin-Protein Ligase Hyperplastic Discs and the

Drosophila GSK3β Homologue, Shaggy. PLoS One. 2015 Sep 3;10(9):e0136760. doi:

10.1371/journal.pone.0136760. PubMed PMID: 26334301; PubMed Central PMCID:

PMC4559392.

8: Handu M, Kaduskar B, Ravindranathan R, Soory A, Giri R, Elango VB, Gowda H,

Ratnaparkhi GS. SUMO-Enriched Proteome for Drosophila Innate Immune Response. G3

(Bethesda). 2015 Aug 18;5(10):2137-54. doi: 10.1534/g3.115.020958. PubMed PMID:

26290570; PubMed Central PMCID: PMC4592996.

9: Petsakou A, Sapsis TP, Blau J. Circadian Rhythms in Rho1 Activity Regulate

Neuronal Plasticity and Network Hierarchy. Cell. 2015 Aug 13;162(4):823-35. doi:

10.1016/j.cell.2015.07.010. PubMed PMID: 26234154; PubMed Central PMCID:

PMC4537806.

10: Pradhan-Sundd T, Verheyen EM. The Myopic-Ubpy-Hrs nexus enables endosomal

recycling of Frizzled. Mol Biol Cell. 2015 Sep 15;26(18):3329-42. doi:

10.1091/mbc.E15-02-0086. PubMed PMID: 26224310; PubMed Central PMCID: PMC4569321.

11: Gombos R, Migh E, Antal O, Mukherjee A, Jenny A, Mihály J. The Formin DAAM

Functions as Molecular Effector of the Planar Cell Polarity Pathway during Axonal

Development in Drosophila. J Neurosci. 2015 Jul 15;35(28):10154-67. doi:

10.1523/JNEUROSCI.3708-14.2015. PubMed PMID: 26180192; PubMed Central PMCID:

PMC4502256.

12: Andersen DS, Colombani J, Palmerini V, Chakrabandhu K, Boone E, Röthlisberger

M, Toggweiler J, Basler K, Mapelli M, Hueber AO, Léopold P. The Drosophila TNF

receptor Grindelwald couples loss of cell polarity and neoplastic growth. Nature.

2015 Jun 25;522(7557):482-6. doi: 10.1038/nature14298. PubMed PMID: 25874673.

13: Baëza M, Viala S, Heim M, Dard A, Hudry B, Duffraisse M, Rogulja-Ortmann A,

Brun C, Merabet S. Inhibitory activities of short linear motifs underlie Hox

interactome specificity in vivo. Elife. 2015 Apr 14;4. doi: 10.7554/eLife.06034.

PubMed PMID: 25869471; PubMed Central PMCID: PMC4392834.

14: Zhang S, Chen C, Wu C, Yang Y, Li W, Xue L. The canonical Wg signaling

modulates Bsk-mediated cell death in Drosophila. Cell Death Dis. 2015 Apr

9;6:e1713. doi: 10.1038/cddis.2015.85. PubMed PMID: 25855961; PubMed Central

PMCID: PMC4650552.

15: Ghosh S, Lasko P. Loss-of-function analysis reveals distinct requirements of

the translation initiation factors eIF4E, eIF4E-3, eIF4G and eIF4G2 in Drosophila

spermatogenesis. PLoS One. 2015 Apr 7;10(4):e0122519. doi:

10.1371/journal.pone.0122519. PubMed PMID: 25849588; PubMed Central PMCID:

PMC4388691.

16: Laflamme C, Emery G. In vitro and in vivo characterization of the Rab11-GAP

activity of Drosophila Evi5. Methods Mol Biol. 2015;1298:187-94. doi:

10.1007/978-1-4939-2569-8_16. PubMed PMID: 25800843.

17: Kim JH, Ren Y, Ng WP, Li S, Son S, Kee YS, Zhang S, Zhang G, Fletcher DA,

Robinson DN, Chen EH. Mechanical tension drives cell membrane fusion. Dev Cell.

2015 Mar 9;32(5):561-73. doi: 10.1016/j.devcel.2015.01.005. PubMed PMID:

25684354; PubMed Central PMCID: PMC4357538.

18: Wen J, Duan H, Bejarano F, Okamura K, Fabian L, Brill JA, Bortolamiol-Becet

D, Martin R, Ruby JG, Lai EC. Adaptive regulation of testis gene expression and

control of male fertility by the Drosophila hairpin RNA pathway. [Corrected]. Mol

Cell. 2015 Jan 8;57(1):165-78. doi: 10.1016/j.molcel.2014.11.025. Erratum in: Mol

Cell. 2015 Jun 18;58(6):1133. PubMed PMID: 25544562; PubMed Central PMCID:

PMC4289472.

19: Gillingham AK, Sinka R, Torres IL, Lilley KS, Munro S. Toward a comprehensive

map of the effectors of rab GTPases. Dev Cell. 2014 Nov 10;31(3):358-73. doi:

10.1016/j.devcel.2014.10.007. PubMed PMID: 25453831; PubMed Central PMCID:

PMC4232348.

20: Andlauer TF, Scholz-Kornehl S, Tian R, Kirchner M, Babikir HA, Depner H, Loll

B, Quentin C, Gupta VK, Holt MG, Dipt S, Cressy M, Wahl MC, Fiala A, Selbach M,

Schwärzel M, Sigrist SJ. Drep-2 is a novel synaptic protein important for

learning and memory. Elife. 2014 Nov 13;3. doi: 10.7554/eLife.03895. PubMed PMID:

25392983; PubMed Central PMCID: PMC4229683.

21: Glowinski C, Liu RH, Chen X, Darabie A, Godt D. Myosin VIIA regulates

microvillus morphogenesis and interacts with cadherin Cad99C in Drosophila

oogenesis. J Cell Sci. 2014 Nov 15;127(Pt 22):4821-32. doi: 10.1242/jcs.099242.

PubMed PMID: 25236597.

22: Alic N, Giannakou ME, Papatheodorou I, Hoddinott MP, Andrews TD, Bolukbasi E,

Partridge L. Interplay of dFOXO and two ETS-family transcription factors

determines lifespan in Drosophila melanogaster. PLoS Genet. 2014 Sep

18;10(9):e1004619. doi: 10.1371/journal.pgen.1004619. PubMed PMID: 25232726;

PubMed Central PMCID: PMC4169242.

23: Fagan JK, Dollar G, Lu Q, Barnett A, Pechuan Jorge J, Schlosser A, Pfleger C,

Adler P, Jenny A. Combover/CG10732, a novel PCP effector for Drosophila wing hair

formation. PLoS One. 2014 Sep 10;9(9):e107311. doi: 10.1371/journal.pone.0107311.

PubMed PMID: 25207969; PubMed Central PMCID: PMC4160248.

24: Wang Y, Yan J, Lee H, Lu Q, Adler PN. The proteins encoded by the Drosophila

Planar Polarity Effector genes inturned, fuzzy and fritz interact physically and

can re-pattern the accumulation of "upstream" Planar Cell Polarity proteins. Dev

Biol. 2014 Oct 1;394(1):156-69. doi: 10.1016/j.ydbio.2014.07.013. PubMed PMID:

25072625; PubMed Central PMCID: PMC4163512.

25: Linnemannstöns K, Ripp C, Honemann-Capito M, Brechtel-Curth K, Hedderich M,

Wodarz A. The PTK7-related transmembrane proteins off-track and off-track 2 are

co-receptors for Drosophila Wnt2 required for male fertility. PLoS Genet. 2014

Jul 10;10(7):e1004443. doi: 10.1371/journal.pgen.1004443. PubMed PMID: 25010066;

PubMed Central PMCID: PMC4091708.

26: Alekseyenko AA, Gorchakov AA, Zee BM, Fuchs SM, Kharchenko PV, Kuroda MI.

Heterochromatin-associated interactions of Drosophila HP1a with dADD1, HIPP1, and

repetitive RNAs. Genes Dev. 2014 Jul 1;28(13):1445-60. doi:

10.1101/gad.241950.114. PubMed PMID: 24990964; PubMed Central PMCID: PMC4083088.

27: Li L, Tian X, Zhu M, Bulgari D, Böhme MA, Goettfert F, Wichmann C, Sigrist

SJ, Levitan ES, Wu C. Drosophila Syd-1, liprin-α, and protein phosphatase 2A B'

subunit Wrd function in a linear pathway to prevent ectopic accumulation of

synaptic materials in distal axons. J Neurosci. 2014 Jun 18;34(25):8474-87. doi:

10.1523/JNEUROSCI.0409-14.2014. PubMed PMID: 24948803; PubMed Central PMCID:

PMC4061390.

28: Zhang T, Liao Y, Hsu FN, Zhang R, Searle JS, Pei X, Li X, Ryoo HD, Ji JY, Du

W. Hyperactivated Wnt signaling induces synthetic lethal interaction with Rb

inactivation by elevating TORC1 activities. PLoS Genet. 2014 May

8;10(5):e1004357. doi: 10.1371/journal.pgen.1004357. PubMed PMID: 24809668;

PubMed Central PMCID: PMC4014429.

29: Bilak A, Uyetake L, Su TT. Dying cells protect survivors from

radiation-induced cell death in Drosophila. PLoS Genet. 2014 Mar

27;10(3):e1004220. doi: 10.1371/journal.pgen.1004220. PubMed PMID: 24675716;

PubMed Central PMCID: PMC3967929.

30: Tutor AS, Prieto-Sánchez S, Ruiz-Gómez M. Src64B phosphorylates Dumbfounded

and regulates slit diaphragm dynamics: Drosophila as a model to study

nephropathies. Development. 2014 Jan;141(2):367-76. doi: 10.1242/dev.099408.

PubMed PMID: 24335255.

31: Komori H, Xiao Q, McCartney BM, Lee CY. Brain tumor specifies intermediate

progenitor cell identity by attenuating β-catenin/Armadillo activity.

Development. 2014 Jan;141(1):51-62. doi: 10.1242/dev.099382. PubMed PMID:

24257623; PubMed Central PMCID: PMC3865749.

32: Lin C, Katanaev VL. Kermit interacts with Gαo, Vang, and motor proteins in

Drosophila planar cell polarity. PLoS One. 2013 Oct 3;8(10):e76885. doi:

10.1371/journal.pone.0076885. PubMed PMID: 24204696; PubMed Central PMCID:

PMC3805608.

33: Sieglitz F, Matzat T, Yuva-Aydemir Y, Neuert H, Altenhein B, Klämbt C.

Antagonistic feedback loops involving Rau and Sprouty in the Drosophila eye

control neuronal and glial differentiation. Sci Signal. 2013 Nov 5;6(300):ra96.

doi: 10.1126/scisignal.2004651. Erratum in: Sci Signal. 2013 Dec 10;6(305):er8.

Yuva-Adyemir, Yeliz [corrected to Yuva-Aydemir, Yeliz]. PubMed PMID: 24194583.

34: Ma X, Li W, Yu H, Yang Y, Li M, Xue L, Xu T. Bendless modulates JNK-mediated

cell death and migration in Drosophila. Cell Death Differ. 2014 Mar;21(3):407-15.

doi: 10.1038/cdd.2013.154. PubMed PMID: 24162658; PubMed Central PMCID:

PMC3921588.

35: Kwon Y, Vinayagam A, Sun X, Dephoure N, Gygi SP, Hong P, Perrimon N. The

Hippo signaling pathway interactome. Science. 2013 Nov 8;342(6159):737-40. doi:

10.1126/science.1243971. PubMed PMID: 24114784; PubMed Central PMCID: PMC3951131.

36: Shaw JL, Chang KT. Nebula/DSCR1 upregulation delays neurodegeneration and

protects against APP-induced axonal transport defects by restoring calcineurin

and GSK-3β signaling. PLoS Genet. 2013;9(9):e1003792. doi:

10.1371/journal.pgen.1003792. PubMed PMID: 24086147; PubMed Central PMCID:

PMC3784514.

37: Chin ML, Mlodzik M. The Drosophila selectin furrowed mediates intercellular

planar cell polarity interactions via frizzled stabilization. Dev Cell. 2013 Sep

16;26(5):455-68. doi: 10.1016/j.devcel.2013.07.006. PubMed PMID: 23973164; PubMed

Central PMCID: PMC4084690.

38: Laver JD, Li X, Ancevicius K, Westwood JT, Smibert CA, Morris QD, Lipshitz

HD. Genome-wide analysis of Staufen-associated mRNAs identifies secondary

structures that confer target specificity. Nucleic Acids Res. 2013

Nov;41(20):9438-60. doi: 10.1093/nar/gkt702. PubMed PMID: 23945942; PubMed

Central PMCID: PMC3814352.

39: D'Brot A, Chen P, Vaishnav M, Yuan S, Akey CW, Abrams JM. Tango7 directs

cellular remodeling by the Drosophila apoptosome. Genes Dev. 2013 Aug

1;27(15):1650-5. doi: 10.1101/gad.219287.113. PubMed PMID: 23913920; PubMed

Central PMCID: PMC3744723.

40: Özkan E, Carrillo RA, Eastman CL, Weiszmann R, Waghray D, Johnson KG, Zinn K,

Celniker SE, Garcia KC. An extracellular interactome of immunoglobulin and LRR

proteins reveals receptor-ligand networks. Cell. 2013 Jul 3;154(1):228-39. doi:

10.1016/j.cell.2013.06.006. PubMed PMID: 23827685; PubMed Central PMCID:

PMC3756661.

41: Iyer J, Wahlmark CJ, Kuser-Ahnert GA, Kawasaki F. Molecular mechanisms of

COMPLEXIN fusion clamp function in synaptic exocytosis revealed in a new

Drosophila mutant. Mol Cell Neurosci. 2013 Sep;56:244-54. doi:

10.1016/j.mcn.2013.06.002. PubMed PMID: 23769723; PubMed Central PMCID:

PMC3791175.

42: Sen A, Dimlich DN, Guruharsha KG, Kankel MW, Hori K, Yokokura T, Brachat S,

Richardson D, Loureiro J, Sivasankaran R, Curtis D, Davidow LS, Rubin LL, Hart

AC, Van Vactor D, Artavanis-Tsakonas S. Genetic circuitry of Survival motor

neuron, the gene underlying spinal muscular atrophy. Proc Natl Acad Sci U S A.

2013 Jun 25;110(26):E2371-80. doi: 10.1073/pnas.1301738110. PubMed PMID:

23757500; PubMed Central PMCID: PMC3696827.

43: Ma X, Yang L, Yang Y, Li M, Li W, Xue L. dUev1a modulates TNF-JNK mediated

tumor progression and cell death in Drosophila. Dev Biol. 2013 Aug

15;380(2):211-21. doi: 10.1016/j.ydbio.2013.05.013. PubMed PMID: 23726905.

44: Hernández G, Miron M, Han H, Liu N, Magescas J, Tettweiler G, Frank F,

Siddiqui N, Sonenberg N, Lasko P. Mextli is a novel eukaryotic translation

initiation factor 4E-binding protein that promotes translation in Drosophila

melanogaster. Mol Cell Biol. 2013 Aug;33(15):2854-64. doi: 10.1128/MCB.01354-12.

PubMed PMID: 23716590; PubMed Central PMCID: PMC3719689.

45: Khan SJ, Bajpai A, Alam MA, Gupta RP, Harsh S, Pandey RK, Goel-Bhattacharya

S, Nigam A, Mishra A, Sinha P. Epithelial neoplasia in Drosophila entails switch

to primitive cell states. Proc Natl Acad Sci U S A. 2013 Jun 11;110(24):E2163-72.

doi: 10.1073/pnas.1212513110. PubMed PMID: 23708122; PubMed Central PMCID:

PMC3683752.

46: Soldano A, Okray Z, Janovska P, Tmejová K, Reynaud E, Claeys A, Yan J, Atak

ZK, De Strooper B, Dura JM, Bryja V, Hassan BA. The Drosophila homologue of the

amyloid precursor protein is a conserved modulator of Wnt PCP signaling. PLoS

Biol. 2013;11(5):e1001562. doi: 10.1371/journal.pbio.1001562. PubMed PMID:

23690751; PubMed Central PMCID: PMC3653798.

47: Zhang Y, Ling J, Yuan C, Dubruille R, Emery P. A role for Drosophila ATX2 in

activation of PER translation and circadian behavior. Science. 2013 May

17;340(6134):879-82. doi: 10.1126/science.1234746. PubMed PMID: 23687048; PubMed

Central PMCID: PMC4078874.

48: Yu XM, Gutman I, Mosca TJ, Iram T, Ozkan E, Garcia KC, Luo L, Schuldiner O.

Plum, an immunoglobulin superfamily protein, regulates axon pruning by

facilitating TGF-β signaling. Neuron. 2013 May 8;78(3):456-68. doi:

10.1016/j.neuron.2013.03.004. PubMed PMID: 23664613; PubMed Central PMCID:

PMC3706783.

49: Ramel D, Wang X, Laflamme C, Montell DJ, Emery G. Rab11 regulates cell-cell

communication during collective cell movements. Nat Cell Biol. 2013

Mar;15(3):317-24. doi: 10.1038/ncb2681. PubMed PMID: 23376974; PubMed Central

PMCID: PMC4006229.

50: Januschke J, Reina J, Llamazares S, Bertran T, Rossi F, Roig J, Gonzalez C.

Centrobin controls mother-daughter centriole asymmetry in Drosophila neuroblasts.

Nat Cell Biol. 2013 Mar;15(3):241-8. doi: 10.1038/ncb2671. PubMed PMID: 23354166.

51: van de Hoef DL, Bonner JM, Boulianne GL. FKBP14 is an essential gene that

regulates Presenilin protein levels and Notch signaling in Drosophila.

Development. 2013 Feb;140(4):810-9. doi: 10.1242/dev.081356. PubMed PMID:

23318643.

52: Wehr MC, Holder MV, Gailite I, Saunders RE, Maile TM, Ciirdaeva E, Instrell

R, Jiang M, Howell M, Rossner MJ, Tapon N. Salt-inducible kinases regulate growth

through the Hippo signalling pathway in Drosophila. Nat Cell Biol. 2013

Jan;15(1):61-71. PubMed PMID: 23263283; PubMed Central PMCID: PMC3749438.

53: Kaipa BR, Shao H, Schäfer G, Trinkewitz T, Groth V, Liu J, Beck L, Bogdan S,

Abmayr SM, Önel SF. Dock mediates Scar- and WASp-dependent actin polymerization

through interaction with cell adhesion molecules in founder cells and

fusion-competent myoblasts. J Cell Sci. 2013 Jan 1;126(Pt 1):360-72. doi:

10.1242/jcs.113860. PubMed PMID: 22992459; PubMed Central PMCID: PMC3603522.

54: Hernández G, Han H, Gandin V, Fabian L, Ferreira T, Zuberek J, Sonenberg N,

Brill JA, Lasko P. Eukaryotic initiation factor 4E-3 is essential for meiotic

chromosome segregation, cytokinesis and male fertility in Drosophila.

Development. 2012 Sep;139(17):3211-20. doi: 10.1242/dev.073122. PubMed PMID:

22833128; PubMed Central PMCID: PMC3413165.

55: Laflamme C, Assaker G, Ramel D, Dorn JF, She D, Maddox PS, Emery G. Evi5

promotes collective cell migration through its Rab-GAP activity. J Cell Biol.

2012 Jul 9;198(1):57-67. doi: 10.1083/jcb.201112114. PubMed PMID: 22778279;

PubMed Central PMCID: PMC3392932.

56: Lewis PW, Sahoo D, Geng C, Bell M, Lipsick JS, Botchan MR. Drosophila lin-52

acts in opposition to repressive components of the Myb-MuvB/dREAM complex. Mol

Cell Biol. 2012 Aug;32(16):3218-27. doi: 10.1128/MCB.00432-12. PubMed PMID:

22688510; PubMed Central PMCID: PMC3434544.

57: Morris DH, Dubnau J, Park JH, Rawls JM Jr. Divergent functions through

alternative splicing: the Drosophila CRMP gene in pyrimidine metabolism, brain,

and behavior. Genetics. 2012 Aug;191(4):1227-38. doi:

10.1534/genetics.112.141101. PubMed PMID: 22649077; PubMed Central PMCID:

PMC3416003.

58: Kanao T, Sawada T, Davies SA, Ichinose H, Hasegawa K, Takahashi R, Hattori N,

Imai Y. The nitric oxide-cyclic GMP pathway regulates FoxO and alters

dopaminergic neuron survival in Drosophila. PLoS One. 2012;7(2):e30958. doi:

10.1371/journal.pone.0030958. PubMed PMID: 22393355; PubMed Central PMCID:

PMC3290610.

59: Mauser JF, Prehoda KE. Inscuteable regulates the Pins-Mud spindle orientation

pathway. PLoS One. 2012;7(1):e29611. doi: 10.1371/journal.pone.0029611. PubMed

PMID: 22253744; PubMed Central PMCID: PMC3254608.

60: Mulligan KA, Fuerer C, Ching W, Fish M, Willert K, Nusse R. Secreted

Wingless-interacting molecule (Swim) promotes long-range signaling by maintaining

Wingless solubility. Proc Natl Acad Sci U S A. 2012 Jan 10;109(2):370-7. doi:

10.1073/pnas.1119197109. PubMed PMID: 22203956; PubMed Central PMCID: PMC3258625.

61: Neumüller RA, Wirtz-Peitz F, Lee S, Kwon Y, Buckner M, Hoskins RA, Venken KJ,

Bellen HJ, Mohr SE, Perrimon N. Stringent analysis of gene function and

protein-protein interactions using fluorescently tagged genes. Genetics. 2012

Mar;190(3):931-40. doi: 10.1534/genetics.111.136465. PubMed PMID: 22174071;

PubMed Central PMCID: PMC3296255.

62: Culurgioni S, Alfieri A, Pendolino V, Laddomada F, Mapelli M. Inscuteable and

NuMA proteins bind competitively to Leu-Gly-Asn repeat-enriched protein (LGN)

during asymmetric cell divisions. Proc Natl Acad Sci U S A. 2011 Dec

27;108(52):20998-1003. doi: 10.1073/pnas.1113077108. PubMed PMID: 22171003;

PubMed Central PMCID: PMC3248549.

63: Zhu S, Barshow S, Wildonger J, Jan LY, Jan YN. Ets transcription factor

Pointed promotes the generation of intermediate neural progenitors in Drosophila

larval brains. Proc Natl Acad Sci U S A. 2011 Dec 20;108(51):20615-20. doi:

10.1073/pnas.1118595109. PubMed PMID: 22143802; PubMed Central PMCID: PMC3251047.

64: Murillo-Maldonado JM, Zeineddine FB, Stock R, Thackeray J, Riesgo-Escovar JR.

Insulin receptor-mediated signaling via phospholipase C-γ regulates growth and

differentiation in Drosophila. PLoS One. 2011;6(11):e28067. doi:

10.1371/journal.pone.0028067. PubMed PMID: 22132213; PubMed Central PMCID:

PMC3221684.

65: Guruharsha KG, Rual JF, Zhai B, Mintseris J, Vaidya P, Vaidya N, Beekman C,

Wong C, Rhee DY, Cenaj O, McKillip E, Shah S, Stapleton M, Wan KH, Yu C, Parsa B,

Carlson JW, Chen X, Kapadia B, VijayRaghavan K, Gygi SP, Celniker SE, Obar RA,

Artavanis-Tsakonas S. A protein complex network of Drosophila melanogaster. Cell.

2011 Oct 28;147(3):690-703. doi: 10.1016/j.cell.2011.08.047. PubMed PMID:

22036573; PubMed Central PMCID: PMC3319048.

66: Friedman AA, Tucker G, Singh R, Yan D, Vinayagam A, Hu Y, Binari R, Hong P,

Sun X, Porto M, Pacifico S, Murali T, Finley RL Jr, Asara JM, Berger B, Perrimon

N. Proteomic and functional genomic landscape of receptor tyrosine kinase and ras

to extracellular signal-regulated kinase signaling. Sci Signal. 2011 Oct

25;4(196):rs10. doi: 10.1126/scisignal.2002029. PubMed PMID: 22028469; PubMed

Central PMCID: PMC3439136.

67: Andrejka L, Wen H, Ashton J, Grant M, Iori K, Wang A, Manak JR, Lipsick JS.

Animal-specific C-terminal domain links myeloblastosis oncoprotein (Myb) to an

ancient repressor complex. Proc Natl Acad Sci U S A. 2011 Oct

18;108(42):17438-43. doi: 10.1073/pnas.1111855108. PubMed PMID: 21969598; PubMed

Central PMCID: PMC3198365.

68: Shi Q, Li S, Jia J, Jiang J. The Hedgehog-induced Smoothened conformational

switch assembles a signaling complex that activates Fused by promoting its

dimerization and phosphorylation. Development. 2011 Oct;138(19):4219-31. doi:

10.1242/dev.067959. PubMed PMID: 21852395; PubMed Central PMCID: PMC3171222.

69: Araújo SJ, Casanova J. Sequoia establishes tip-cell number in Drosophila

trachea by regulating FGF levels. J Cell Sci. 2011 Jul 15;124(Pt 14):2335-40.

doi: 10.1242/jcs.085613. PubMed PMID: 21693579.

70: Kaplan NA, Colosimo PF, Liu X, Tolwinski NS. Complex interactions between

GSK3 and aPKC in Drosophila embryonic epithelial morphogenesis. PLoS One. 2011

Apr 5;6(4):e18616. doi: 10.1371/journal.pone.0018616. PubMed PMID: 21483653;

PubMed Central PMCID: PMC3071738.

71: Jiang Y, Scott KL, Kwak SJ, Chen R, Mardon G. Sds22/PP1 links epithelial

integrity and tumor suppression via regulation of myosin II and JNK signaling.

Oncogene. 2011 Jul 21;30(29):3248-60. doi: 10.1038/onc.2011.46. PubMed PMID:

21399659; PubMed Central PMCID: PMC3141090.

72: Ohsawa S, Sugimura K, Takino K, Xu T, Miyawaki A, Igaki T. Elimination of

oncogenic neighbors by JNK-mediated engulfment in Drosophila. Dev Cell. 2011 Mar

15;20(3):315-28. doi: 10.1016/j.devcel.2011.02.007. PubMed PMID: 21397843.

73: Yu W, Kawasaki F, Ordway RW. Activity-dependent interactions of NSF and SNAP

at living synapses. Mol Cell Neurosci. 2011 May;47(1):19-27. doi:

10.1016/j.mcn.2011.02.002. PubMed PMID: 21316453; PubMed Central PMCID:

PMC3094158.

74: Szuperák M, Salah S, Meyer EJ, Nagarajan U, Ikmi A, Gibson MC. Feedback

regulation of Drosophila BMP signaling by the novel extracellular protein larval

translucida. Development. 2011 Feb;138(4):715-24. doi: 10.1242/dev.059477. PubMed

PMID: 21266407.

75: Hou Q, Jiang H, Zhang X, Guo C, Huang B, Wang P, Wang T, Wu K, Li J, Gong Z,

Du L, Liu Y, Liu L, Chen C. Nitric oxide metabolism controlled by formaldehyde

dehydrogenase (fdh, homolog of mammalian GSNOR) plays a crucial role in visual

pattern memory in Drosophila. Nitric Oxide. 2011 Jan 1;24(1):17-24. doi:

10.1016/j.niox.2010.09.007. PubMed PMID: 20932929.

76: Georgiev P, Okkenhaug H, Drews A, Wright D, Lambert S, Flick M, Carta V,

Martel C, Oberwinkler J, Raghu P. TRPM channels mediate zinc homeostasis and

cellular growth during Drosophila larval development. Cell Metab. 2010 Oct

6;12(4):386-97. doi: 10.1016/j.cmet.2010.08.012. PubMed PMID: 20889130.

77: Krahn MP, Bückers J, Kastrup L, Wodarz A. Formation of a Bazooka-Stardust

complex is essential for plasma membrane polarity in epithelia. J Cell Biol. 2010

Sep 6;190(5):751-60. doi: 10.1083/jcb.201006029. PubMed PMID: 20819933; PubMed

Central PMCID: PMC2935580.

78: Wang Z, Chapman ER. Rat and Drosophila synaptotagmin 4 have opposite effects

during SNARE-catalyzed membrane fusion. J Biol Chem. 2010 Oct 1;285(40):30759-66.

doi: 10.1074/jbc.M110.137745. PubMed PMID: 20688915; PubMed Central PMCID:

PMC2945570.

79: Bao S, Fischbach KF, Corbin V, Cagan RL. Preferential adhesion maintains

separation of ommatidia in the Drosophila eye. Dev Biol. 2010 Aug

15;344(2):948-56. doi: 10.1016/j.ydbio.2010.06.013. PubMed PMID: 20599904; PubMed

Central PMCID: PMC2921583.

80: Franke JD, Montague RA, Kiehart DP. Nonmuscle myosin II is required for cell

proliferation, cell sheet adhesion and wing hair morphology during wing

morphogenesis. Dev Biol. 2010 Sep 15;345(2):117-32. doi:

10.1016/j.ydbio.2010.06.028. PubMed PMID: 20599890; PubMed Central PMCID:

PMC3712330.

81: Buechling T, Bartscherer K, Ohkawara B, Chaudhary V, Spirohn K, Niehrs C,

Boutros M. Wnt/Frizzled signaling requires dPRR, the Drosophila homolog of the

prorenin receptor. Curr Biol. 2010 Jul 27;20(14):1263-8. doi:

10.1016/j.cub.2010.05.028. PubMed PMID: 20579883.

82: Hermle T, Saltukoglu D, Grünewald J, Walz G, Simons M. Regulation of

Frizzled-dependent planar polarity signaling by a V-ATPase subunit. Curr Biol.

2010 Jul 27;20(14):1269-76. doi: 10.1016/j.cub.2010.05.057. PubMed PMID:

20579879.

83: Rendina R, Strangi A, Avallone B, Giordano E. Bap170, a subunit of the

Drosophila PBAP chromatin remodeling complex, negatively regulates the EGFR

signaling. Genetics. 2010 Sep;186(1):167-81. doi: 10.1534/genetics.110.118695.

PubMed PMID: 20551433; PubMed Central PMCID: PMC2940285.

84: Pataki C, Matusek T, Kurucz E, Andó I, Jenny A, Mihály J. Drosophila Rab23 is

involved in the regulation of the number and planar polarization of the adult

cuticular hairs. Genetics. 2010 Apr;184(4):1051-65. doi:

10.1534/genetics.109.112060. PubMed PMID: 20124028; PubMed Central PMCID:

PMC2865907.

85: Wu M, Pastor-Pareja JC, Xu T. Interaction between Ras(V12) and scribbled

clones induces tumour growth and invasion. Nature. 2010 Jan 28;463(7280):545-8.

doi: 10.1038/nature08702. PubMed PMID: 20072127; PubMed Central PMCID:

PMC2835536.

86: Fetting JL, Spencer SA, Wolff T. The cell adhesion molecules Echinoid and

Friend of Echinoid coordinate cell adhesion and cell signaling to regulate the

fidelity of ommatidial rotation in the Drosophila eye. Development. 2009

Oct;136(19):3323-33. doi: 10.1242/dev.038422. PubMed PMID: 19736327; PubMed

Central PMCID: PMC2739146.

87: Courbard JR, Djiane A, Wu J, Mlodzik M. The apical/basal-polarity determinant

Scribble cooperates with the PCP core factor Stbm/Vang and functions as one of

its effectors. Dev Biol. 2009 Sep 1;333(1):67-77. doi:

10.1016/j.ydbio.2009.06.024. PubMed PMID: 19563796; PubMed Central PMCID:

PMC3011816.

88: Raghu P, Coessens E, Manifava M, Georgiev P, Pettitt T, Wood E,

Garcia-Murillas I, Okkenhaug H, Trivedi D, Zhang Q, Razzaq A, Zaid O, Wakelam M,

O'Kane CJ, Ktistakis N. Rhabdomere biogenesis in Drosophila photoreceptors is

acutely sensitive to phosphatidic acid levels. J Cell Biol. 2009 Apr

6;185(1):129-45. doi: 10.1083/jcb.200807027. PubMed PMID: 19349583; PubMed

Central PMCID: PMC2700502.

89: Igaki T, Pastor-Pareja JC, Aonuma H, Miura M, Xu T. Intrinsic tumor

suppression and epithelial maintenance by endocytic activation of Eiger/TNF

signaling in Drosophila. Dev Cell. 2009 Mar;16(3):458-65. doi:

10.1016/j.devcel.2009.01.002. PubMed PMID: 19289090; PubMed Central PMCID:

PMC2729686.

90: Shelton C, Kocherlakota KS, Zhuang S, Abmayr SM. The immunoglobulin

superfamily member Hbs functions redundantly with Sns in interactions between

founder and fusion-competent myoblasts. Development. 2009 Apr;136(7):1159-68.

doi: 10.1242/dev.026302. PubMed PMID: 19270174; PubMed Central PMCID: PMC2685934.

91: Simons M, Gault WJ, Gotthardt D, Rohatgi R, Klein TJ, Shao Y, Lee HJ, Wu AL,

Fang Y, Satlin LM, Dow JT, Chen J, Zheng J, Boutros M, Mlodzik M. Electrochemical

cues regulate assembly of the Frizzled/Dishevelled complex at the plasma membrane

during planar epithelial polarization. Nat Cell Biol. 2009 Mar;11(3):286-94. doi:

10.1038/ncb1836. Erratum in: Nat Cell Biol. 2009 Apr;11(4):508. PubMed PMID:

19234454; PubMed Central PMCID: PMC2803043.

92: Ida H, Suzusho N, Suyari O, Yoshida H, Ohno K, Hirose F, Itoh M, Yamaguchi M.

Genetic screening for modifiers of the DREF pathway in Drosophila melanogaster:

identification and characterization of HP6 as a novel target of DREF. Nucleic

Acids Res. 2009 Apr;37(5):1423-37. doi: 10.1093/nar/gkn1068. PubMed PMID:

19136464; PubMed Central PMCID: PMC2655671.

93: Tolwinski NS. Membrane bound axin is sufficient for Wingless signaling in

Drosophila embryos. Genetics. 2009 Mar;181(3):1169-73. doi:

10.1534/genetics.108.098236. PubMed PMID: 19124571; PubMed Central PMCID:

PMC2651051.

94: Gervais L, Claret S, Januschke J, Roth S, Guichet A. PIP5K-dependent

production of PIP2 sustains microtubule organization to establish polarized

transport in the Drosophila oocyte. Development. 2008 Dec;135(23):3829-38. doi:

10.1242/dev.029009. Erratum in: Development. 2008 Dec;135(23):3970. PubMed PMID:

18948416.

95: Franciscovich AL, Mortimer AD, Freeman AA, Gu J, Sanyal S. Overexpression

screen in Drosophila identifies neuronal roles of GSK-3 beta/shaggy as a

regulator of AP-1-dependent developmental plasticity. Genetics. 2008

Dec;180(4):2057-71. doi: 10.1534/genetics.107.085555. PubMed PMID: 18832361;

PubMed Central PMCID: PMC2600941.

96: Wu J, Mlodzik M. The frizzled extracellular domain is a ligand for Van

Gogh/Stbm during nonautonomous planar cell polarity signaling. Dev Cell. 2008

Sep;15(3):462-9. doi: 10.1016/j.devcel.2008.08.004. PubMed PMID: 18804440; PubMed

Central PMCID: PMC2814157.

97: Morris EJ, Ji JY, Yang F, Di Stefano L, Herr A, Moon NS, Kwon EJ, Haigis KM,

Näär AM, Dyson NJ. E2F1 represses beta-catenin transcription and is antagonized

by both pRB and CDK8. Nature. 2008 Sep 25;455(7212):552-6. doi:

10.1038/nature07310. PubMed PMID: 18794899; PubMed Central PMCID: PMC3148807.

98: Gregory SL, Shandala T, O'Keefe L, Jones L, Murray MJ, Saint R. A Drosophila

overexpression screen for modifiers of Rho signalling in cytokinesis. Fly

(Austin). 2007 Jan-Feb;1(1):13-22. PubMed PMID: 18690061.

99: Mitchell N, Cranna N, Richardson H, Quinn L. The Ecdysone-inducible

zinc-finger transcription factor Crol regulates Wg transcription and cell cycle

progression in Drosophila. Development. 2008 Aug;135(16):2707-16. doi:

10.1242/dev.021766. PubMed PMID: 18614577.

100: Chen WS, Antic D, Matis M, Logan CY, Povelones M, Anderson GA, Nusse R,

Axelrod JD. Asymmetric homotypic interactions of the atypical cadherin flamingo

mediate intercellular polarity signaling. Cell. 2008 Jun 13;133(6):1093-105. doi:

10.1016/j.cell.2008.04.048. PubMed PMID: 18555784; PubMed Central PMCID:

PMC2446404.

101: Serpe M, Umulis D, Ralston A, Chen J, Olson DJ, Avanesov A, Othmer H,

O'Connor MB, Blair SS. The BMP-binding protein Crossveinless 2 is a short-range,

concentration-dependent, biphasic modulator of BMP signaling in Drosophila. Dev

Cell. 2008 Jun;14(6):940-53. doi: 10.1016/j.devcel.2008.03.023. PubMed PMID:

18539121; PubMed Central PMCID: PMC2488203.

102: Weber U, Pataki C, Mihaly J, Mlodzik M. Combinatorial signaling by the

Frizzled/PCP and Egfr pathways during planar cell polarity establishment in the

Drosophila eye. Dev Biol. 2008 Apr 1;316(1):110-23. doi:

10.1016/j.ydbio.2008.01.016. PubMed PMID: 18291359; PubMed Central PMCID:

PMC2579749.

103: Bejarano F, Luque CM, Herranz H, Sorrosal G, Rafel N, Pham TT, Milán M. A

gain-of-function suppressor screen for genes involved in dorsal-ventral boundary

formation in the Drosophila wing. Genetics. 2008 Jan;178(1):307-23. doi:

10.1534/genetics.107.081869. PubMed PMID: 18202376; PubMed Central PMCID:

PMC2206080.

104: Wu J, Jenny A, Mirkovic I, Mlodzik M. Frizzled-Dishevelled signaling

specificity outcome can be modulated by Diego in Drosophila. Mech Dev. 2008

Jan-Feb;125(1-2):30-42. PubMed PMID: 18065209; PubMed Central PMCID: PMC2800357.

105: Rawls AS, Schultz SA, Mitra RD, Wolff T. Bedraggled, a putative transporter,

influences the tissue polarity complex during the R3/R4 fate decision in the

Drosophila eye. Genetics. 2007 Sep;177(1):313-28. PubMed PMID: 17890365; PubMed

Central PMCID: PMC2013731.

106: Bastock R, Strutt D. The planar polarity pathway promotes coordinated cell

migration during Drosophila oogenesis. Development. 2007 Sep;134(17):3055-64.

PubMed PMID: 17652348; PubMed Central PMCID: PMC1991286.

107: Dietzl G, Chen D, Schnorrer F, Su KC, Barinova Y, Fellner M, Gasser B,

Kinsey K, Oppel S, Scheiblauer S, Couto A, Marra V, Keleman K, Dickson BJ. A

genome-wide transgenic RNAi library for conditional gene inactivation in

Drosophila. Nature. 2007 Jul 12;448(7150):151-6. PubMed PMID: 17625558.

108: Harris KE, Beckendorf SK. Different Wnt signals act through the Frizzled and

RYK receptors during Drosophila salivary gland migration. Development. 2007

Jun;134(11):2017-25. PubMed PMID: 17507403.

109: Gawliński P, Nikolay R, Goursot C, Lawo S, Chaurasia B, Herz HM,

Kussler-Schneider Y, Ruppert T, Mayer M, Grosshans J. The Drosophila mitotic

inhibitor Frühstart specifically binds to the hydrophobic patch of cyclins. EMBO

Rep. 2007 May;8(5):490-6. PubMed PMID: 17431409; PubMed Central PMCID:

PMC1866202.

110: Stoleru D, Nawathean P, Fernández MP, Menet JS, Ceriani MF, Rosbash M. The

Drosophila circadian network is a seasonal timer. Cell. 2007 Apr 6;129(1):207-19.

PubMed PMID: 17418796.

111: Chung S, Kim S, Yoon J, Adler PN, Yim J. The balance between the novel

protein target of wingless and the Drosophila Rho-associated kinase pathway

regulates planar cell polarity in the Drosophila wing. Genetics. 2007

Jun;176(2):891-903. PubMed PMID: 17409077; PubMed Central PMCID: PMC1894616.

112: Kalamegham R, Sturgill D, Siegfried E, Oliver B. Drosophila mojoless, a

retroposed GSK-3, has functionally diverged to acquire an essential role in male

fertility. Mol Biol Evol. 2007 Mar;24(3):732-42. PubMed PMID: 17179138; PubMed

Central PMCID: PMC2292417.

113: Singh A, Shi X, Choi KW. Lobe and Serrate are required for cell survival

during early eye development in Drosophila. Development. 2006

Dec;133(23):4771-81. PubMed PMID: 17090721.

114: Uhlirova M, Bohmann D. JNK- and Fos-regulated Mmp1 expression cooperates

with Ras to induce invasive tumors in Drosophila. EMBO J. 2006 Nov

15;25(22):5294-304. PubMed PMID: 17082773; PubMed Central PMCID: PMC1636619.

115: Srahna M, Leyssen M, Choi CM, Fradkin LG, Noordermeer JN, Hassan BA. A

signaling network for patterning of neuronal connectivity in the Drosophila

brain. PLoS Biol. 2006 Oct;4(11):e348. Erratum in: PLoS Biol. 2006

Dec;4(12):e432. PubMed PMID: 17032066; PubMed Central PMCID: PMC1592317.

116: Klein TJ, Jenny A, Djiane A, Mlodzik M. CKIepsilon/discs overgrown promotes

both Wnt-Fz/beta-catenin and Fz/PCP signaling in Drosophila. Curr Biol. 2006 Jul

11;16(13):1337-43. PubMed PMID: 16824922.

117: Strutt H, Price MA, Strutt D. Planar polarity is positively regulated by

casein kinase Iepsilon in Drosophila. Curr Biol. 2006 Jul 11;16(13):1329-36.

PubMed PMID: 16824921.

118: Kreisköther N, Reichert N, Buttgereit D, Hertenstein A, Fischbach KF,

Renkawitz-Pohl R. Drosophila rolling pebbles colocalises and putatively interacts

with alpha-Actinin and the Sls isoform Zormin in the Z-discs of the sarcomere and

with Dumbfounded/Kirre, alpha-Actinin and Zormin in the terminal Z-discs. J

Muscle Res Cell Motil. 2006;27(1):93-106. PubMed PMID: 16699917.

119: Katanaev VL, Tomlinson A. Dual roles for the trimeric G protein Go in

asymmetric cell division in Drosophila. Proc Natl Acad Sci U S A. 2006 Apr

25;103(17):6524-9. PubMed PMID: 16617104; PubMed Central PMCID: PMC1436022.

120: Seto ES, Bellen HJ. Internalization is required for proper Wingless

signaling in Drosophila melanogaster. J Cell Biol. 2006 Apr 10;173(1):95-106.

PubMed PMID: 16606693; PubMed Central PMCID: PMC2063794.

121: Blanke S, Jäckle H. Novel guanine nucleotide exchange factor GEFmeso of

Drosophila melanogaster interacts with Ral and Rho GTPase Cdc42. FASEB J. 2006

Apr;20(6):683-91. PubMed PMID: 16581976.

122: Wang X, Bo J, Bridges T, Dugan KD, Pan TC, Chodosh LA, Montell DJ. Analysis

of cell migration using whole-genome expression profiling of migratory cells in

the Drosophila ovary. Dev Cell. 2006 Apr;10(4):483-95. PubMed PMID: 16580993.

123: Garcia-Murillas I, Pettitt T, Macdonald E, Okkenhaug H, Georgiev P, Trivedi

D, Hassan B, Wakelam M, Raghu P. lazaro encodes a lipid phosphate

phosphohydrolase that regulates phosphatidylinositol turnover during Drosophila

phototransduction. Neuron. 2006 Feb 16;49(4):533-46. PubMed PMID: 16476663.

124: Charroux B, Freeman M, Kerridge S, Baonza A. Atrophin contributes to the

negative regulation of epidermal growth factor receptor signaling in Drosophila.

Dev Biol. 2006 Mar 15;291(2):278-90. PubMed PMID: 16445904.

125: Mohit P, Makhijani K, Madhavi MB, Bharathi V, Lal A, Sirdesai G, Reddy VR,

Ramesh P, Kannan R, Dhawan J, Shashidhara LS. Modulation of AP and DV signaling

pathways by the homeotic gene Ultrabithorax during haltere development in

Drosophila. Dev Biol. 2006 Mar 15;291(2):356-67. PubMed PMID: 16414040.

126: Oishi K, Gaengel K, Krishnamoorthy S, Kamiya K, Kim IK, Ying H, Weber U,

Perkins LA, Tartaglia M, Mlodzik M, Pick L, Gelb BD. Transgenic Drosophila models

of Noonan syndrome causing PTPN11 gain-of-function mutations. Hum Mol Genet. 2006

Feb 15;15(4):543-53. PubMed PMID: 16399795.

127: Mukherjee T, Schäfer U, Zeidler MP. Identification of Drosophila genes

modulating Janus kinase/signal transducer and activator of transcription signal

transduction. Genetics. 2006 Mar;172(3):1683-97. PubMed PMID: 16387886; PubMed

Central PMCID: PMC1456271.

128: Vishnu S, Hertenstein A, Betschinger J, Knoblich JA, Gert de Couet H,

Fischbach KF. The adaptor protein X11Lalpha/Dmint1 interacts with the PDZ-binding

domain of the cell recognition protein Rst in Drosophila. Dev Biol. 2006 Jan

15;289(2):296-307. PubMed PMID: 16380111.

129: Ren N, He B, Stone D, Kirakodu S, Adler PN. The shavenoid gene of Drosophila

encodes a novel actin cytoskeleton interacting protein that promotes wing hair

morphogenesis. Genetics. 2006 Mar;172(3):1643-53. PubMed PMID: 16322503; PubMed

Central PMCID: PMC1456309.

130: Kanuka H, Kuranaga E, Takemoto K, Hiratou T, Okano H, Miura M. Drosophila

caspase transduces Shaggy/GSK-3beta kinase activity in neural precursor

development. EMBO J. 2005 Nov 2;24(21):3793-806. PubMed PMID: 16222340; PubMed

Central PMCID: PMC1276714.

131: Uhlirova M, Jasper H, Bohmann D. Non-cell-autonomous induction of tissue

overgrowth by JNK/Ras cooperation in a Drosophila tumor model. Proc Natl Acad Sci

U S A. 2005 Sep 13;102(37):13123-8. PubMed PMID: 16150723; PubMed Central PMCID:

PMC1201591.

132: Yuan Q, Lin F, Zheng X, Sehgal A. Serotonin modulates circadian entrainment

in Drosophila. Neuron. 2005 Jul 7;47(1):115-27. PubMed PMID: 15996552.

133: Lim J, Norga KK, Chen Z, Choi KW. Control of planar cell polarity by

interaction of DWnt4 and four-jointed. Genesis. 2005 Jul;42(3):150-61. PubMed

PMID: 15986451.

134: Singh A, Chan J, Chern JJ, Choi KW. Genetic interaction of Lobe with its

modifiers in dorsoventral patterning and growth of the Drosophila eye. Genetics.

2005 Sep;171(1):169-83. PubMed PMID: 15976174; PubMed Central PMCID: PMC1456509.

135: Read RD, Goodfellow PJ, Mardis ER, Novak N, Armstrong JR, Cagan RL. A

Drosophila model of multiple endocrine neoplasia type 2. Genetics. 2005

Nov;171(3):1057-81. PubMed PMID: 15965261; PubMed Central PMCID: PMC1456812.

136: Jenny A, Reynolds-Kenneally J, Das G, Burnett M, Mlodzik M. Diego and

Prickle regulate Frizzled planar cell polarity signalling by competing for

Dishevelled binding. Nat Cell Biol. 2005 Jul;7(7):691-7. PubMed PMID: 15937478.

137: Djiane A, Yogev S, Mlodzik M. The apical determinants aPKC and dPatj

regulate Frizzled-dependent planar cell polarity in the Drosophila eye. Cell.

2005 May 20;121(4):621-31. PubMed PMID: 15907474.

138: Lai EC, Tam B, Rubin GM. Pervasive regulation of Drosophila Notch target

genes by GY-box-, Brd-box-, and K-box-class microRNAs. Genes Dev. 2005 May

1;19(9):1067-80. PubMed PMID: 15833912; PubMed Central PMCID: PMC1091741.

139: Ralston A, Blair SS. Long-range Dpp signaling is regulated to restrict BMP

signaling to a crossvein competent zone. Dev Biol. 2005 Apr 1;280(1):187-200.

PubMed PMID: 15766758.

140: Collier S, Lee H, Burgess R, Adler P. The WD40 repeat protein fritz links

cytoskeletal planar polarity to frizzled subcellular localization in the

Drosophila epidermis. Genetics. 2005 Apr;169(4):2035-45. PubMed PMID: 15654087;

PubMed Central PMCID: PMC1449578.

141: Katanaev VL, Ponzielli R, Sémériva M, Tomlinson A. Trimeric G

protein-dependent frizzled signaling in Drosophila. Cell. 2005 Jan

14;120(1):111-22. PubMed PMID: 15652486.

142: Mace K, Tugores A. The product of the split ends gene is required for the

maintenance of positional information during Drosophila development. BMC Dev

Biol. 2004 Dec 13;4:15. PubMed PMID: 15596016; PubMed Central PMCID: PMC544560.

143: Ng J, Luo L. Rho GTPases regulate axon growth through convergent and

divergent signaling pathways. Neuron. 2004 Dec 2;44(5):779-93. PubMed PMID:

15572110.

144: Hughes JR, Bullock SL, Ish-Horowicz D. Inscuteable mRNA localization is

dynein-dependent and regulates apicobasal polarity and spindle length in

Drosophila neuroblasts. Curr Biol. 2004 Nov 9;14(21):1950-6. PubMed PMID:

15530398.

145: Vivekanand P, Tootle TL, Rebay I. MAE, a dual regulator of the EGFR

signaling pathway, is a target of the Ets transcription factors PNT and YAN. Mech

Dev. 2004 Dec;121(12):1469-79. PubMed PMID: 15511639.

146: Galletta BJ, Chakravarti M, Banerjee R, Abmayr SM. SNS: Adhesive properties,

localization requirements and ectodomain dependence in S2 cells and embryonic

myoblasts. Mech Dev. 2004 Dec;121(12):1455-68. PubMed PMID: 15511638.

147: Schlesinger A, Kiger A, Perrimon N, Shilo BZ. Small wing PLCgamma is

required for ER retention of cleaved Spitz during eye development in Drosophila.

Dev Cell. 2004 Oct;7(4):535-45. PubMed PMID: 15469842.

148: Wilk R, Pickup AT, Hamilton JK, Reed BH, Lipshitz HD. Dose-sensitive

autosomal modifiers identify candidate genes for tissue autonomous and tissue

nonautonomous regulation by the Drosophila nuclear zinc-finger protein,

hindsight. Genetics. 2004 Sep;168(1):281-300. PubMed PMID: 15454543; PubMed

Central PMCID: PMC1448082.

149: Brumby A, Secombe J, Horsfield J, Coombe M, Amin N, Coates D, Saint R,

Richardson H. A genetic screen for dominant modifiers of a cyclin E hypomorphic

mutation identifies novel regulators of S-phase entry in Drosophila. Genetics.

2004 Sep;168(1):227-51. PubMed PMID: 15454540; PubMed Central PMCID: PMC1448096.

150: Lawrence PA, Casal J, Struhl G. Cell interactions and planar polarity in the

abdominal epidermis of Drosophila. Development. 2004 Oct;131(19):4651-64. PubMed

PMID: 15329345.

151: Cheng MK, Shearn A. The direct interaction between ASH2, a Drosophila

trithorax group protein, and SKTL, a nuclear phosphatidylinositol 4-phosphate

5-kinase, implies a role for phosphatidylinositol 4,5-bisphosphate in maintaining

transcriptionally active chromatin. Genetics. 2004 Jul;167(3):1213-23. PubMed

PMID: 15280236; PubMed Central PMCID: PMC1470965.

152: Franco B, Bogdanik L, Bobinnec Y, Debec A, Bockaert J, Parmentier ML, Grau

Y. Shaggy, the homolog of glycogen synthase kinase 3, controls neuromuscular

junction growth in Drosophila. J Neurosci. 2004 Jul 21;24(29):6573-7. PubMed

PMID: 15269269.

153: Wu J, Klein TJ, Mlodzik M. Subcellular localization of frizzled receptors,

mediated by their cytoplasmic tails, regulates signaling pathway specificity.

PLoS Biol. 2004 Jul;2(7):E158. PubMed PMID: 15252441; PubMed Central PMCID:

PMC449784.

154: Morel V, Arias AM. Armadillo/beta-catenin-dependent Wnt signalling is

required for the polarisation of epidermal cells during dorsal closure in

Drosophila. Development. 2004 Jul;131(14):3273-83. PubMed PMID: 15226252.

155: Zeng YA, Verheyen EM. Nemo is an inducible antagonist of Wingless signaling

during Drosophila wing development. Development. 2004 Jun;131(12):2911-20. PubMed

PMID: 15169756.

156: Bhat KM, Apsel N. Upregulation of Mitimere and Nubbin acts through cyclin E

to confer self-renewing asymmetric division potential to neural precursor cells.

Development. 2004 Mar;131(5):1123-34. PubMed PMID: 14973280.

157: Bajpai R, Makhijani K, Rao PR, Shashidhara LS. Drosophila Twins regulates

Armadillo levels in response to Wg/Wnt signal. Development. 2004

Mar;131(5):1007-16. PubMed PMID: 14973271.

158: Matsubayashi H, Sese S, Lee JS, Shirakawa T, Iwatsubo T, Tomita T, Yanagawa

S. Biochemical characterization of the Drosophila wingless signaling pathway

based on RNA interference. Mol Cell Biol. 2004 Mar;24(5):2012-24. PubMed PMID:

14966281; PubMed Central PMCID: PMC350544.

159: Irion U, Leptin M, Siller K, Fuerstenberg S, Cai Y, Doe CQ, Chia W, Yang X.

Abstrakt, a DEAD box protein, regulates Insc levels and asymmetric division of

neural and mesodermal progenitors. Curr Biol. 2004 Jan 20;14(2):138-44. PubMed

PMID: 14738736.

160: Lee H, Adler PN. The grainy head transcription factor is essential for the

function of the frizzled pathway in the Drosophila wing. Mech Dev. 2004

Jan;121(1):37-49. PubMed PMID: 14706698.

161: Wong HC, Bourdelas A, Krauss A, Lee HJ, Shao Y, Wu D, Mlodzik M, Shi DL,

Zheng J. Direct binding of the PDZ domain of Dishevelled to a conserved internal

sequence in the C-terminal region of Frizzled. Mol Cell. 2003 Nov;12(5):1251-60.

PubMed PMID: 14636582; PubMed Central PMCID: PMC4381837.

162: Brumby AM, Richardson HE. scribble mutants cooperate with oncogenic Ras or

Notch to cause neoplastic overgrowth in Drosophila. EMBO J. 2003 Nov

3;22(21):5769-79. PubMed PMID: 14592975; PubMed Central PMCID: PMC275405.

163: Pagliarini RA, Xu T. A genetic screen in Drosophila for metastatic behavior.

Science. 2003 Nov 14;302(5648):1227-31. PubMed PMID: 14551319.

164: Gaengel K, Mlodzik M. Egfr signaling regulates ommatidial rotation and cell

motility in the Drosophila eye via MAPK/Pnt signaling and the Ras effector

Canoe/AF6. Development. 2003 Nov;130(22):5413-23. Erratum in: Development. 2003

Dec;130(23):5861. PubMed PMID: 14507782.

165: Woodhouse EC, Fisher A, Bandle RW, Bryant-Greenwood B, Charboneau L,

Petricoin EF 3rd, Liotta LA. Drosophila screening model for metastasis:

Semaphorin 5c is required for l(2)gl cancer phenotype. Proc Natl Acad Sci U S A.

2003 Sep 30;100(20):11463-8. PubMed PMID: 14500904; PubMed Central PMCID:

PMC208780.

166: Jenny A, Darken RS, Wilson PA, Mlodzik M. Prickle and Strabismus form a

functional complex to generate a correct axis during planar cell polarity

signaling. EMBO J. 2003 Sep 1;22(17):4409-20. PubMed PMID: 12941693; PubMed

Central PMCID: PMC202366.

167: Strutt H, Strutt D. EGF signaling and ommatidial rotation in the Drosophila

eye. Curr Biol. 2003 Aug 19;13(16):1451-7. PubMed PMID: 12932331.

168: Yu F, Cai Y, Kaushik R, Yang X, Chia W. Distinct roles of Galphai and

Gbeta13F subunits of the heterotrimeric G protein complex in the mediation of

Drosophila neuroblast asymmetric divisions. J Cell Biol. 2003 Aug

18;162(4):623-33. PubMed PMID: 12925708; PubMed Central PMCID: PMC2173805.

169: Yamada T, Okabe M, Hiromi Y. EDL/MAE regulates EGF-mediated induction by

antagonizing Ets transcription factor Pointed. Development. 2003

Sep;130(17):4085-96. PubMed PMID: 12874129.

170: Schweizer L, Nellen D, Basler K. Requirement for Pangolin/dTCF in Drosophila

Wingless signaling. Proc Natl Acad Sci U S A. 2003 May 13;100(10):5846-51. PubMed

PMID: 12730381; PubMed Central PMCID: PMC156289.

171: Lai EC. Drosophila tufted is a gain-of-function allele of the proneural gene

amos. Genetics. 2003 Apr;163(4):1413-25. PubMed PMID: 12702685; PubMed Central

PMCID: PMC1462509.

172: Chandra S, Ahmed A, Vaessin H. The Drosophila IgC2 domain protein

Friend-of-Echinoid, a paralogue of Echinoid, limits the number of sensory organ

precursors in the wing disc and interacts with the Notch signaling pathway. Dev

Biol. 2003 Apr 15;256(2):302-16. PubMed PMID: 12679104.

173: Kim S, Shi H, Lee DK, Lis JT. Specific SR protein-dependent splicing

substrates identified through genomic SELEX. Nucleic Acids Res. 2003 Apr

1;31(7):1955-61. PubMed PMID: 12655012; PubMed Central PMCID: PMC152802.

174: Tolwinski NS, Wehrli M, Rives A, Erdeniz N, DiNardo S, Wieschaus E. Wg/Wnt

signal can be transmitted through arrow/LRP5,6 and Axin independently of

Zw3/Gsk3beta activity. Dev Cell. 2003 Mar;4(3):407-18. PubMed PMID: 12636921.

175: Yang L, Baker NE. Cell cycle withdrawal, progression, and cell survival

regulation by EGFR and its effectors in the differentiating Drosophila eye. Dev

Cell. 2003 Mar;4(3):359-69. PubMed PMID: 12636917.

176: Marygold SJ, Vincent JP. Armadillo levels are reduced during mitosis in

Drosophila. Mech Dev. 2003 Feb;120(2):157-65. PubMed PMID: 12559488.

177: Ma D, Yang CH, McNeill H, Simon MA, Axelrod JD. Fidelity in planar cell

polarity signalling. Nature. 2003 Jan 30;421(6922):543-7. PubMed PMID: 12540853.

178: Cai Y, Yu F, Lin S, Chia W, Yang X. Apical complex genes control mitotic

spindle geometry and relative size of daughter cells in Drosophila neuroblast and

pI asymmetric divisions. Cell. 2003 Jan 10;112(1):51-62. PubMed PMID: 12526793.

179: Bilder D, Schober M, Perrimon N. Integrated activity of PDZ protein

complexes regulates epithelial polarity. Nat Cell Biol. 2003 Jan;5(1):53-8.

PubMed PMID: 12510194.

180: Tanentzapf G, Tepass U. Interactions between the crumbs, lethal giant larvae

and bazooka pathways in epithelial polarization. Nat Cell Biol. 2003

Jan;5(1):46-52. PubMed PMID: 12510193.

181: Tan C, Stronach B, Perrimon N. Roles of myosin phosphatase during Drosophila

development. Development. 2003 Feb;130(4):671-81. PubMed PMID: 12505998.

182: Baonza A, Murawsky CM, Travers AA, Freeman M. Pointed and Tramtrack69

establish an EGFR-dependent transcriptional switch to regulate mitosis. Nat Cell

Biol. 2002 Dec;4(12):976-80. PubMed PMID: 12447387.

183: Vilinsky I, Stewart BA, Drummond J, Robinson I, Deitcher DL. A Drosophila

SNAP-25 null mutant reveals context-dependent redundancy with SNAP-24 in

neurotransmission. Genetics. 2002 Sep;162(1):259-71. PubMed PMID: 12242238;

PubMed Central PMCID: PMC1462260.

184: Wu CH, Nusse R. Ligand receptor interactions in the Wnt signaling pathway in

Drosophila. J Biol Chem. 2002 Nov 1;277(44):41762-9. Erratum in: J Biol Chem.

2005 Sep 2;280(35):31340. PubMed PMID: 12205098.

185: Amorós M, Corominas M, Deák P, Serras F. The ash2 gene is involved in

Drosophila wing development. Int J Dev Biol. 2002 May;46(3):321-4. PubMed PMID:

12068954.

186: Sullivan KM, Rubin GM. The Ca(2+)-calmodulin-activated protein phosphatase

calcineurin negatively regulates EGF receptor signaling in Drosophila

development. Genetics. 2002 May;161(1):183-93. PubMed PMID: 12019233; PubMed

Central PMCID: PMC1462097.

187: Tree DR, Shulman JM, Rousset R, Scott MP, Gubb D, Axelrod JD. Prickle

mediates feedback amplification to generate asymmetric planar cell polarity

signaling. Cell. 2002 May 3;109(3):371-81. PubMed PMID: 12015986.

188: Parker DS, Jemison J, Cadigan KM. Pygopus, a nuclear PHD-finger protein

required for Wingless signaling in Drosophila. Development. 2002

Jun;129(11):2565-76. PubMed PMID: 12015286.

189: Lee H, Adler PN. The function of the frizzled pathway in the Drosophila wing

is dependent on inturned and fuzzy. Genetics. 2002 Apr;160(4):1535-47. PubMed

PMID: 11973308; PubMed Central PMCID: PMC1462037.

190: Rohrbaugh M, Ramos E, Nguyen D, Price M, Wen Y, Lai ZC. Notch activation of

yan expression is antagonized by RTK/pointed signaling in the Drosophila eye.

Curr Biol. 2002 Apr 2;12(7):576-81. PubMed PMID: 11937027.

191: Jia J, Amanai K, Wang G, Tang J, Wang B, Jiang J. Shaggy/GSK3 antagonizes

Hedgehog signalling by regulating Cubitus interruptus. Nature. 2002 Apr

4;416(6880):548-52. PubMed PMID: 11912487.

192: Peña-Rangel MT, Rodriguez I, Riesgo-Escovar JR. A misexpression study

examining dorsal thorax formation in Drosophila melanogaster. Genetics. 2002

Mar;160(3):1035-50. PubMed PMID: 11901120; PubMed Central PMCID: PMC1462010.

193: Yang CH, Axelrod JD, Simon MA. Regulation of Frizzled by fat-like cadherins

during planar polarity signaling in the Drosophila compound eye. Cell. 2002 Mar

8;108(5):675-88. PubMed PMID: 11893338.

194: Strapps WR, Tomlinson A. Transducing properties of Drosophila Frizzled

proteins. Development. 2001 Dec;128(23):4829-35. PubMed PMID: 11731462.

195: Ramain P, Khechumian K, Seugnet L, Arbogast N, Ackermann C, Heitzler P.

Novel Notch alleles reveal a Deltex-dependent pathway repressing neural fate.

Curr Biol. 2001 Nov 13;11(22):1729-38. PubMed PMID: 11719214.

196: Chen EH, Olson EN. Antisocial, an intracellular adaptor protein, is required

for myoblast fusion in Drosophila. Dev Cell. 2001 Nov;1(5):705-15. PubMed PMID:

11709190.

197: Bulgheresi S, Kleiner E, Knoblich JA. Inscuteable-dependent apical

localization of the microtubule-binding protein Cornetto suggests a role in

asymmetric cell division. J Cell Sci. 2001 Oct;114(Pt 20):3655-62. PubMed PMID:

11707517.

198: Dworak HA, Charles MA, Pellerano LB, Sink H. Characterization of Drosophila

hibris, a gene related to human nephrin. Development. 2001 Nov;128(21):4265-76.

PubMed PMID: 11684662.

199: Schaefer M, Petronczki M, Dorner D, Forte M, Knoblich JA. Heterotrimeric G

proteins direct two modes of asymmetric cell division in the Drosophila nervous

system. Cell. 2001 Oct 19;107(2):183-94. PubMed PMID: 11672526.

200: Schnorr JD, Holdcraft R, Chevalier B, Berg CA. Ras1 interacts with multiple

new signaling and cytoskeletal loci in Drosophila eggshell patterning and

morphogenesis. Genetics. 2001 Oct;159(2):609-22. PubMed PMID: 11606538; PubMed

Central PMCID: PMC1461825.

201: Kammerer M, Giangrande A. Glide2, a second glial promoting factor in

Drosophila melanogaster. EMBO J. 2001 Sep 3;20(17):4664-73. PubMed PMID:

11532931; PubMed Central PMCID: PMC125586.

202: Ciapponi L, Jackson DB, Mlodzik M, Bohmann D. Drosophila Fos mediates ERK

and JNK signals via distinct phosphorylation sites. Genes Dev. 2001 Jun

15;15(12):1540-53. PubMed PMID: 11410534; PubMed Central PMCID: PMC312716.

203: Winter CG, Wang B, Ballew A, Royou A, Karess R, Axelrod JD, Luo L.

Drosophila Rho-associated kinase (Drok) links Frizzled-mediated planar cell

polarity signaling to the actin cytoskeleton. Cell. 2001 Apr 6;105(1):81-91.

PubMed PMID: 11301004.

204: Rousset R, Mack JA, Wharton KA Jr, Axelrod JD, Cadigan KM, Fish MP, Nusse R,

Scott MP. Naked cuticle targets dishevelled to antagonize Wnt signal

transduction. Genes Dev. 2001 Mar 15;15(6):658-71. PubMed PMID: 11274052; PubMed

Central PMCID: PMC312650.

205: Bai J, Chiu W, Wang J, Tzeng T, Perrimon N, Hsu J. The cell adhesion

molecule Echinoid defines a new pathway that antagonizes the Drosophila EGF

receptor signaling pathway. Development. 2001 Feb;128(4):591-601. PubMed PMID:

11171342.

206: Schaefer M, Shevchenko A, Shevchenko A, Knoblich JA. A protein complex

containing Inscuteable and the Galpha-binding protein Pins orients asymmetric

cell divisions in Drosophila. Curr Biol. 2000 Apr 6;10(7):353-62. PubMed PMID:

10753746.

207: Bellaïche Y, Gho M, Kaltschmidt JA, Brand AH, Schweisguth F. Frizzled

regulates localization of cell-fate determinants and mitotic spindle rotation

during asymmetric cell division. Nat Cell Biol. 2001 Jan;3(1):50-7. PubMed PMID:

11146626.

208: Collins RT, Treisman JE. Osa-containing Brahma chromatin remodeling

complexes are required for the repression of wingless target genes. Genes Dev.

2000 Dec 15;14(24):3140-52. PubMed PMID: 11124806; PubMed Central PMCID:

PMC317146.

209: Therrien M, Morrison DK, Wong AM, Rubin GM. A genetic screen for modifiers

of a kinase suppressor of Ras-dependent rough eye phenotype in Drosophila.

Genetics. 2000 Nov;156(3):1231-42. PubMed PMID: 11063697; PubMed Central PMCID:

PMC1461306.

210: Huang AM, Rubin GM. A misexpression screen identifies genes that can

modulate RAS1 pathway signaling in Drosophila melanogaster. Genetics. 2000

Nov;156(3):1219-30. PubMed PMID: 11063696; PubMed Central PMCID: PMC1461302.

211: Firth L, Manchester J, Lorenzen JA, Baron M, Perkins LA. Identification of

genomic regions that interact with a viable allele of the Drosophila protein

tyrosine phosphatase corkscrew. Genetics. 2000 Oct;156(2):733-48. PubMed PMID:

11014820; PubMed Central PMCID: PMC1461264.

212: Moline MM, Dierick HA, Southern C, Bejsovec A. Non-equivalent roles of

Drosophila Frizzled and Dfrizzled2 in embryonic wingless signal transduction.

Curr Biol. 2000 Sep 21;10(18):1127-30. PubMed PMID: 10996794.

213: Sone M, Suzuki E, Hoshino M, Hou D, Kuromi H, Fukata M, Kuroda S, Kaibuchi

K, Nabeshima Y, Hama C. Synaptic development is controlled in the periactive

zones of Drosophila synapses. Development. 2000 Oct;127(19):4157-68. PubMed PMID:

10976048.

214: Adler PN, Taylor J, Charlton J. The domineering non-autonomy of frizzled and

van Gogh clones in the Drosophila wing is a consequence of a disruption in local

signaling. Mech Dev. 2000 Sep;96(2):197-207. PubMed PMID: 10960784.

215: Chen F, Rebay I. split ends, a new component of the Drosophila EGF receptor

pathway, regulates development of midline glial cells. Curr Biol. 2000 Jul 27-Aug

10;10(15):943-6. PubMed PMID: 10959845.

216: Conley CA, Silburn R, Singer MA, Ralston A, Rohwer-Nutter D, Olson DJ,

Gelbart W, Blair SS. Crossveinless 2 contains cysteine-rich domains and is

required for high levels of BMP-like activity during the formation of the cross

veins in Drosophila. Development. 2000 Sep;127(18):3947-59. PubMed PMID:

10952893.

217: Rulifson EJ, Wu CH, Nusse R. Pathway specificity by the bifunctional

receptor frizzled is determined by affinity for wingless. Mol Cell. 2000

Jul;6(1):117-26. PubMed PMID: 10949033.

218: Cox RT, McEwen DG, Myster DL, Duronio RJ, Loureiro J, Peifer M. A screen for

mutations that suppress the phenotype of Drosophila armadillo, the beta-catenin

homolog. Genetics. 2000 Aug;155(4):1725-40. PubMed PMID: 10924470; PubMed Central

PMCID: PMC1461219.

219: Weber U, Paricio N, Mlodzik M. Jun mediates Frizzled-induced R3/R4 cell fate

distinction and planar polarity determination in the Drosophila eye. Development.

2000 Aug;127(16):3619-29. PubMed PMID: 10903185.

220: Bilder D, Li M, Perrimon N. Cooperative regulation of cell polarity and

growth by Drosophila tumor suppressors. Science. 2000 Jul 7;289(5476):113-6.

PubMed PMID: 10884224.

221: Abdelilah-Seyfried S, Chan YM, Zeng C, Justice NJ, Younger-Shepherd S, Sharp

LE, Barbel S, Meadows SA, Jan LY, Jan YN. A gain-of-function screen for genes

that affect the development of the Drosophila adult external sensory organ.

Genetics. 2000 Jun;155(2):733-52. Erratum in: Genetics 2001 Jan;157(1):457.

PubMed PMID: 10835395; PubMed Central PMCID: PMC1461115.

222: Zeng W, Wharton KA Jr, Mack JA, Wang K, Gadbaw M, Suyama K, Klein PS, Scott

MP. naked cuticle encodes an inducible antagonist of Wnt signalling. Nature. 2000

Feb 17;403(6771):789-95. PubMed PMID: 10693810.

223: Yu F, Morin X, Cai Y, Yang X, Chia W. Analysis of partner of inscuteable, a

novel player of Drosophila asymmetric divisions, reveals two distinct steps in

inscuteable apical localization. Cell. 2000 Feb 18;100(4):399-409. PubMed PMID:

10693757.

224: Rebay I, Chen F, Hsiao F, Kolodziej PA, Kuang BH, Laverty T, Suh C, Voas M,

Williams A, Rubin GM. A genetic screen for novel components of the

Ras/Mitogen-activated protein kinase signaling pathway that interact with the yan

gene of Drosophila identifies split ends, a new RNA recognition motif-containing

protein. Genetics. 2000 Feb;154(2):695-712. PubMed PMID: 10655223; PubMed Central

PMCID: PMC1460949.

225: Schober M, Schaefer M, Knoblich JA. Bazooka recruits Inscuteable to orient

asymmetric cell divisions in Drosophila neuroblasts. Nature. 1999 Dec

2;402(6761):548-51. PubMed PMID: 10591217.

226: Wodarz A, Ramrath A, Kuchinke U, Knust E. Bazooka provides an apical cue for

Inscuteable localization in Drosophila neuroblasts. Nature. 1999 Dec

2;402(6761):544-7. PubMed PMID: 10591216.

227: Brennan K, Tateson R, Lieber T, Couso JP, Zecchini V, Arias AM. The abruptex

mutations of notch disrupt the establishment of proneural clusters in Drosophila.

Dev Biol. 1999 Dec 1;216(1):230-42. PubMed PMID: 10588874.

228: Greaves S, Sanson B, White P, Vincent JP. A screen for identifying genes

interacting with armadillo, the Drosophila homolog of beta-catenin. Genetics.

1999 Dec;153(4):1753-66. PubMed PMID: 10581282; PubMed Central PMCID: PMC1460857.

229: Chen CM, Struhl G. Wingless transduction by the Frizzled and Frizzled2

proteins of Drosophila. Development. 1999 Dec;126(23):5441-52. PubMed PMID:

10556068.

230: Chae J, Kim MJ, Goo JH, Collier S, Gubb D, Charlton J, Adler PN, Park WJ.

The Drosophila tissue polarity gene starry night encodes a member of the

protocadherin family. Development. 1999 Dec;126(23):5421-9. PubMed PMID:

10556066.

231: Helms W, Lee H, Ammerman M, Parks AL, Muskavitch MA, Yedvobnick B.

Engineered truncations in the Drosophila mastermind protein disrupt Notch pathway

function. Dev Biol. 1999 Nov 15;215(2):358-74. PubMed PMID: 10545243.

232: Fanto M, Mlodzik M. Asymmetric Notch activation specifies photoreceptors R3

and R4 and planar polarity in the Drosophila eye. Nature. 1999 Feb

11;397(6719):523-6. PubMed PMID: 10028968.

233: Knoblich JA, Jan LY, Jan YN. Deletion analysis of the Drosophila Inscuteable

protein reveals domains for cortical localization and asymmetric localization.

Curr Biol. 1999 Feb 11;9(3):155-8. PubMed PMID: 10021388.

234: Hummel T, Schimmelpfeng K, Klämbt C. Commissure formation in the embryonic

CNS of Drosophila. Development. 1999 Feb;126(4):771-9. PubMed PMID: 9895324.

235: Kurada P, White K. Ras promotes cell survival in Drosophila by

downregulating hid expression. Cell. 1998 Oct 30;95(3):319-29. PubMed PMID:

9814703.

236: Thackeray JR, Gaines PC, Ebert P, Carlson JR. small wing encodes a

phospholipase C-(gamma) that acts as a negative regulator of R7 development in

Drosophila. Development. 1998 Dec;125(24):5033-42. PubMed PMID: 9811587.

237: Waltzer L, Bienz M. Drosophila CBP represses the transcription factor TCF to

antagonize Wingless signalling. Nature. 1998 Oct 1;395(6701):521-5. PubMed PMID:

9774110.

238: Taylor J, Abramova N, Charlton J, Adler PN. Van Gogh: a new Drosophila

tissue polarity gene. Genetics. 1998 Sep;150(1):199-210. PubMed PMID: 9725839;

PubMed Central PMCID: PMC1460309.

239: Hazelett DJ, Bourouis M, Walldorf U, Treisman JE. decapentaplegic and

wingless are regulated by eyes absent and eyegone and interact to direct the

pattern of retinal differentiation in the eye disc. Development. 1998

Sep;125(18):3741-51. PubMed PMID: 9716539.

240: Axelrod JD, Miller JR, Shulman JM, Moon RT, Perrimon N. Differential

recruitment of Dishevelled provides signaling specificity in the planar cell

polarity and Wingless signaling pathways. Genes Dev. 1998 Aug 15;12(16):2610-22.

PubMed PMID: 9716412; PubMed Central PMCID: PMC317102.

241: Hayashi T, Kojima T, Saigo K. Specification of primary pigment cell and

outer photoreceptor fates by BarH1 homeobox gene in the developing Drosophila

eye. Dev Biol. 1998 Aug 15;200(2):131-45. PubMed PMID: 9705222.

242: Zhang J, Carthew RW. Interactions between Wingless and DFz2 during

Drosophila wing development. Development. 1998 Aug;125(16):3075-85. PubMed PMID:

9671581.

243: Ahmed Y, Hayashi S, Levine A, Wieschaus E. Regulation of armadillo by a

Drosophila APC inhibits neuronal apoptosis during retinal development. Cell. 1998

Jun 26;93(7):1171-82. PubMed PMID: 9657150.

244: Shen CP, Knoblich JA, Chan YM, Jiang MM, Jan LY, Jan YN. Miranda as a

multidomain adapter linking apically localized Inscuteable and basally localized

Staufen and Prospero during asymmetric cell division in Drosophila. Genes Dev.

1998 Jun 15;12(12):1837-46. PubMed PMID: 9637685; PubMed Central PMCID:

PMC316910.

245: Adler PN, Charlton J, Liu J. Mutations in the cadherin superfamily member

gene dachsous cause a tissue polarity phenotype by altering frizzled signaling.

Development. 1998 Mar;125(5):959-68. PubMed PMID: 9449678.

246: Fedorowicz GM, Fry JD, Anholt RR, Mackay TF. Epistatic interactions between

smell-impaired loci in Drosophila melanogaster. Genetics. 1998

Apr;148(4):1885-91. PubMed PMID: 9560402; PubMed Central PMCID: PMC1460070.

247: Isaksson A, Peverali FA, Kockel L, Mlodzik M, Bohmann D. The

deubiquitination enzyme fat facets negatively regulates RTK/Ras/MAPK signalling

during Drosophila eye development. Mech Dev. 1997 Nov;68(1-2):59-67. PubMed PMID:

9431804.

248: Li P, Yang X, Wasser M, Cai Y, Chia W. Inscuteable and Staufen mediate

asymmetric localization and segregation of prospero RNA during Drosophila

neuroblast cell divisions. Cell. 1997 Aug 8;90(3):437-47. PubMed PMID: 9267024.

249: Strutt DI, Weber U, Mlodzik M. The role of RhoA in tissue polarity and

Frizzled signalling. Nature. 1997 May 15;387(6630):292-5. PubMed PMID: 9153394.

250: Verheyen EM, Purcell KJ, Fortini ME, Artavanis-Tsakonas S. Analysis of

dominant enhancers and suppressors of activated Notch in Drosophila. Genetics.

1996 Nov;144(3):1127-41. PubMed PMID: 8913755; PubMed Central PMCID: PMC1207606.

251: Kauffmann RC, Li S, Gallagher PA, Zhang J, Carthew RW. Ras1 signaling and

transcriptional competence in the R7 cell of Drosophila. Genes Dev. 1996 Sep

1;10(17):2167-78. PubMed PMID: 8804311.

252: Cadigan KM, Nusse R. wingless signaling in the Drosophila eye and embryonic

epidermis. Development. 1996 Sep;122(9):2801-12. PubMed PMID: 8787754.

253: Bhanot P, Brink M, Samos CH, Hsieh JC, Wang Y, Macke JP, Andrew D, Nathans

J, Nusse R. A new member of the frizzled family from Drosophila functions as a

Wingless receptor. Nature. 1996 Jul 18;382(6588):225-30. PubMed PMID: 8717036.

254: Leviten MW, Posakony JW. Gain-of-function alleles of Bearded interfere with

alternative cell fate decisions in Drosophila adult sensory organ development.

Dev Biol. 1996 Jun 15;176(2):264-83. PubMed PMID: 8660866.

255: Treier M, Bohmann D, Mlodzik M. JUN cooperates with the ETS domain protein

pointed to induce photoreceptor R7 fate in the Drosophila eye. Cell. 1995 Dec

1;83(5):753-60. PubMed PMID: 8521492.

256: O'Neill EM, Rebay I, Tjian R, Rubin GM. The activities of two Ets-related

transcription factors required for Drosophila eye development are modulated by

the Ras/MAPK pathway. Cell. 1994 Jul 15;78(1):137-47. PubMed PMID: 8033205.

257: Bang AG, Hartenstein V, Posakony JW. Hairless is required for the

development of adult sensory organ precursor cells in Drosophila. Development.

1991 Jan;111(1):89-104. PubMed PMID: 2015800.

258: Perrone RD, Steinman TI, Beck GJ, Skibinski CI, Royal HD, Lawlor M,

Hunsicker LG. Utility of radioisotopic filtration markers in chronic renal

insufficiency: simultaneous comparison of 125I-iothalamate, 169Yb-DTPA,

99mTc-DTPA, and inulin. The Modification of Diet in Renal Disease Study. Am J

Kidney Dis. 1990 Sep;16(3):224-35. PubMed PMID: 2205098.

259: Bilioni A, Sánchez-Hernández D, Callejo A, Gradilla AC, Ibáñez C, Mollica E,

Carmen Rodríguez-Navas M, Simon E, Guerrero I. Balancing Hedgehog, a retention

and release equilibrium given by Dally, Ihog, Boi and shifted/DmWif. Dev Biol.

2013 Apr 15;376(2):198-212. doi: 10.1016/j.ydbio.2012.12.013. PubMed PMID:

23276604.

260: Hayashi Y, Sexton TR, Dejima K, Perry DW, Takemura M, Kobayashi S, Nakato H,

Harrison DA. Glypicans regulate JAK/STAT signaling and distribution of the

Unpaired morphogen. Development. 2012 Nov;139(22):4162-71. doi:

10.1242/dev.078055. PubMed PMID: 23093424; PubMed Central PMCID: PMC3478685.

261: Cho JY, Chak K, Andreone BJ, Wooley JR, Kolodkin AL. The extracellular matrix

proteoglycan perlecan facilitates transmembrane semaphorin-mediated repulsive

guidance. Genes Dev. 2012 Oct 1;26(19):2222-35. doi: 10.1101/gad.193136.112.

PubMed PMID: 23028146; PubMed Central PMCID: PMC3465742.

262: You J, Belenkaya T, Lin X. Sulfated is a negative feedback regulator of

wingless in Drosophila. Dev Dyn. 2011 Mar;240(3):640-8. doi: 10.1002/dvdy.22562.

PubMed PMID: 21305649; PubMed Central PMCID: PMC3071797.

263: Liu M, Lim TM, Cai Y. The Drosophila female germline stem cell lineage acts to

spatially restrict DPP function within the niche. Sci Signal. 2010 Jul

27;3(132):ra57. doi: 10.1126/scisignal.2000740. PubMed PMID: 20664066.

264: Kleinschmit A, Koyama T, Dejima K, Hayashi Y, Kamimura K, Nakato H. Drosophila

heparan sulfate 6-O endosulfatase regulates Wingless morphogen gradient

formation. Dev Biol. 2010 Sep 15;345(2):204-14. doi: 10.1016/j.ydbio.2010.07.006.

PubMed PMID: 20637191; PubMed Central PMCID: PMC2963650.

265: Guo Z, Wang Z. The glypican Dally is required in the niche for the maintenance

of germline stem cells and short-range BMP signaling in the Drosophila ovary.

Development. 2009 Nov;136(21):3627-35. doi: 10.1242/dev.036939. PubMed PMID:

19793889.

266: Baron MJ, Wong SL, Nybakken K, Carey VJ, Madoff LC. Host glycosaminoglycan

confers susceptibility to bacterial infection in Drosophila melanogaster. Infect

Immun. 2009 Feb;77(2):860-6. doi: 10.1128/IAI.00995-08. PubMed PMID: 19047407;

PubMed Central PMCID: PMC2632041.

267: Eugster C, Panáková D, Mahmoud A, Eaton S. Lipoprotein-heparan sulfate

interactions in the Hh pathway. Dev Cell. 2007 Jul;13(1):57-71. PubMed PMID:

17609110.

268: Kirkpatrick CA, Knox SM, Staatz WD, Fox B, Lercher DM, Selleck SB. The

function of a Drosophila glypican does not depend entirely on heparan sulfate

modification. Dev Biol. 2006 Dec 15;300(2):570-82. PubMed PMID: 17055473.

269: Takeo S, Akiyama T, Firkus C, Aigaki T, Nakato H. Expression of a secreted

form of Dally, a Drosophila glypican, induces overgrowth phenotype by affecting

action range of Hedgehog. Dev Biol. 2005 Aug 1;284(1):204-18. PubMed PMID:

15963974.

270: Franch-Marro X, Marchand O, Piddini E, Ricardo S, Alexandre C, Vincent JP.

Glypicans shunt the Wingless signal between local signalling and further

transport. Development. 2005 Feb;132(4):659-66. PubMed PMID: 15647318.

271: Lüders F, Segawa H, Stein D, Selva EM, Perrimon N, Turco SJ, Häcker U. Slalom

encodes an adenosine 3'-phosphate 5'-phosphosulfate transporter essential for

development in Drosophila. EMBO J. 2003 Jul 15;22(14):3635-44. PubMed PMID:

12853478; PubMed Central PMCID: PMC165615.

272: Nakato H, Fox B, Selleck SB. dally, a Drosophila member of the glypican

family of integral membrane proteoglycans, affects cell cycle progression and

morphogenesis via a Cyclin A-mediated process. J Cell Sci. 2002 Jan 1;115(Pt

1):123-30. PubMed PMID: 11801730.

273: Fujise M, Izumi S, Selleck SB, Nakato H. Regulation of dally, an integral

membrane proteoglycan, and its function during adult sensory organ formation of

Drosophila. Dev Biol. 2001 Jul 15;235(2):433-48. PubMed PMID: 11437449.

274: Baeg GH, Lin X, Khare N, Baumgartner S, Perrimon N. Heparan sulfate

proteoglycans are critical for the organization of the extracellular distribution

of Wingless. Development. 2001 Jan;128(1):87-94. PubMed PMID: 11092814.

275: Lin X, Perrimon N. Dally cooperates with Drosophila Frizzled 2 to transduce

Wingless signalling. Nature. 1999 Jul 15;400(6741):281-4. PubMed PMID: 10421372.

276: Tsuda M, Kamimura K, Nakato H, Archer M, Staatz W, Fox B, Humphrey M, Olson

S, Futch T, Kaluza V, Siegfried E, Stam L, Selleck SB. The cell-surface

proteoglycan Dally regulates Wingless signalling in Drosophila. Nature. 1999 Jul

15;400(6741):276-80. PubMed PMID: 10421371.

277: Jackson SM, Nakato H, Sugiura M, Jannuzi A, Oakes R, Kaluza V, Golden C,

Selleck SB. dally, a Drosophila glypican, controls cellular responses to the

TGF-beta-related morphogen, Dpp. Development. 1997 Oct;124(20):4113-20. PubMed

PMID: 9374407.
